# Utility of G protein-coupled oestrogen receptor 1 as a biomarker for pan-cancer diagnosis, prognosis and immune infiltration: a comprehensive bioinformatics analysis

**DOI:** 10.18632/aging.205162

**Published:** 2023-11-02

**Authors:** Yu-Chao Fan, Wen Wu, Xue-Feng Leng, Hong-Wei Zhang

**Affiliations:** 1 Department of Anesthesiology, Sichuan Cancer Center, Sichuan Cancer Hospital and Institute, School of Medicine, University of Electronic Science and Technology of China, Chengdu, Sichuan Province, China; 2 Division of Thoracic Surgery, Sichuan Cancer Center, Sichuan Cancer Hospital and Institute, School of Medicine, University of Electronic Science and Technology of China, Chengdu, Sichuan Province, China; 3 Department of Anesthesiology, Xichang People’s Hospital, Xichang, Sichuan, China

**Keywords:** GPER1, pan-cancer analysis, diagnosis, prognosis, tumor immunity

## Abstract

Background: The G protein-coupled oestrogen receptor (*GPER*) 1 mediates non-genomic oestrogen-related signalling and plays an important role in the regulation of cell growth and programmed cell death through multiple downstream pathways. Despite the increasing interest in the role of *GPER1* in cancer development, no pan-cancer analysis has been available for *GPER1*.

Methods: In this study we performed a comprehensive analysis of the role of *GPER1* in pan-cancer via Human Protein Atlas (HPA), The Cancer Genome Atlas (TCGA), University of California, Santa Cruz Xena (UCSC XENA), Genotype-Tissue Expression (GTEx), MethSurv, The University of Alabama at Birmingham CANcer data analysis Portal (UALCAN), cBioPortal, STRING and TISIDB detabases, followed by enrichment analysis using R software.

Results: *GPER1* was widely expressed in tissues and organs and differed in expression from normal tissue in a variety of cancers. In diagnostic assessment, it’s Area Under the Curve (AUC) surpassed 0.9 in nine cancer types. Survival analysis showed that *GPER1* was correlated with the prognosis of 11 cancer types. Moreover, *GPER1* expression was associated with immune infiltration in multiple cancers.

Conclusions: In summary, *GPER1* has good diagnostic or prognostic value across various malignancies. Together with its extensive correlation with immune components, the aforementioned results suggests that *GPER1* shows promise in tumour diagnosis and prognosis, providing new ideas for precise and personalised anti-tumour strategies.

## INTRODUCTION

Cancer, which is currently the leading cause of death in most of the world’s population, has been considered an important factor hindering the increase of human life expectancy [1]. With an ageing and rapidly growing population, as well as accelerating socioeconomic development, the burden of cancer continues to increase in both developed and developing countries [2]. The most recent data estimates that approximately 160 cancer cases will be diagnosed for every 1,889,000 Americans [3]. By 2021, 608,570 Americans will die from cancer, which is equivalent to more than 1,600 deaths per day [3]. Data for China, the world’s most populous country with an estimated 1.42 billion people, suggested that approximately 4.51 million cancer cases and 3.04 million cancer-related deaths had occurred in 2020 [4]. Despite the differing levels of social or economic development, an increase in cancer incidence or mortality represents a great threat to individual health and a significant economic burden for any country and society.

Therefore, a better approach to cancer prevention and detection is clearly needed. Tumour biomarkers have a wide range of promising clinical applications. They can be used for cancer risk assessment, screening, surveillance, diagnosis, predicting treatment response and monitoring disease progression and recurrence [5, 6], while having the potential to become an important component of precision cancer management [7].

Apart from its critical role in female sexual development and reproductive processes, estrogen is also extensively involved in physiological and pathophysiological processes across different tissues in both sexes [8]. It exerts significant influence in carcinogenesis by regulating cell apoptosis, proliferation, and the cell cycle [9–11]. Additionally, it interacts with various cell types within the tumor microenvironment, including fibroblasts, immune cells, and adipocytes [11]. Among the recognized estrogen receptors (ERs), namely ERα, ERβ, and G-protein-coupled estrogen receptor 1 (*GPER1*), the first two are classical estrogen receptors. *GPER1* serves as a receptor for mediating rapid estrogen effects. Encoded by the *GPER1* gene, *GPER1* is widely expressed in the human body across multiple systems such as reproductive, digestive, cardiovascular, respiratory, nervous, and hematopoietic systems [12, 13]. This receptor binds to estrogen and activates multiple downstream signaling pathways, mediating rapid non-genomic estrogen signaling events. It exerts diverse biological effects in tumor cell proliferation, apoptosis, migration, tumor initiation, and metastasis across various cancers [14]. Recent reports highlight a significant correlation between *GPER1* and the progression of diverse cancers. Furthermore, *GPER1* is considered a potential therapeutic target for cancer treatment [13, 15]. Despite comprehensive pan-cancer analyses exploring ERs as prognostic markers and therapeutic targets across different cancers [16], a comprehensive pan-cancer analysis of *GPER1* is lacking to date. Consequently, this study aims to investigate the diagnostic and prognostic significance of *GPER1* expression in the context of pan-cancer.

The expression of *GPER1* mRNA and protein in various tissues and organs throughout the body was explored using the Human Protein Atlas (HPA) database. Subsequently, *GPER1* expression in tumour tissues was assessed and compared to than in normal and paracancerous tissues using three databases, The Cancer Genome Atlas (TCGA), University of California, Santa Cruz Xena (UCSC XENA) and Genotype-Tissue Expression (GTEx) (https://gtexportal.org/). Promoter methylation of *GPER1* was explored using the MethSurv and The University of Alabama at Birmingham CANcer data analysis Portal (UALCAN) databases. Genetic alterations and their associated survival analysis were evaluated via the cBioPortal. Differentially expressed genes (DEGs) related to *GPER1* expression, Protein–Protein Interaction (PPI), functional enrichment and Gene Set Enrichment Analysis (GSEA) of DEGs were also explored. We further investigated the relationship between *GPER1* and tumour-infiltrating lymphocytes (TILs), immunoinhibitors, immunostimulators, major histocompatibility complex (MHC) molecules, chemokines and chemokine receptors via the TISDB.

## RESULTS

### Expression landscape and pan-cancer expression of *GPER1*

**Figure 1:**
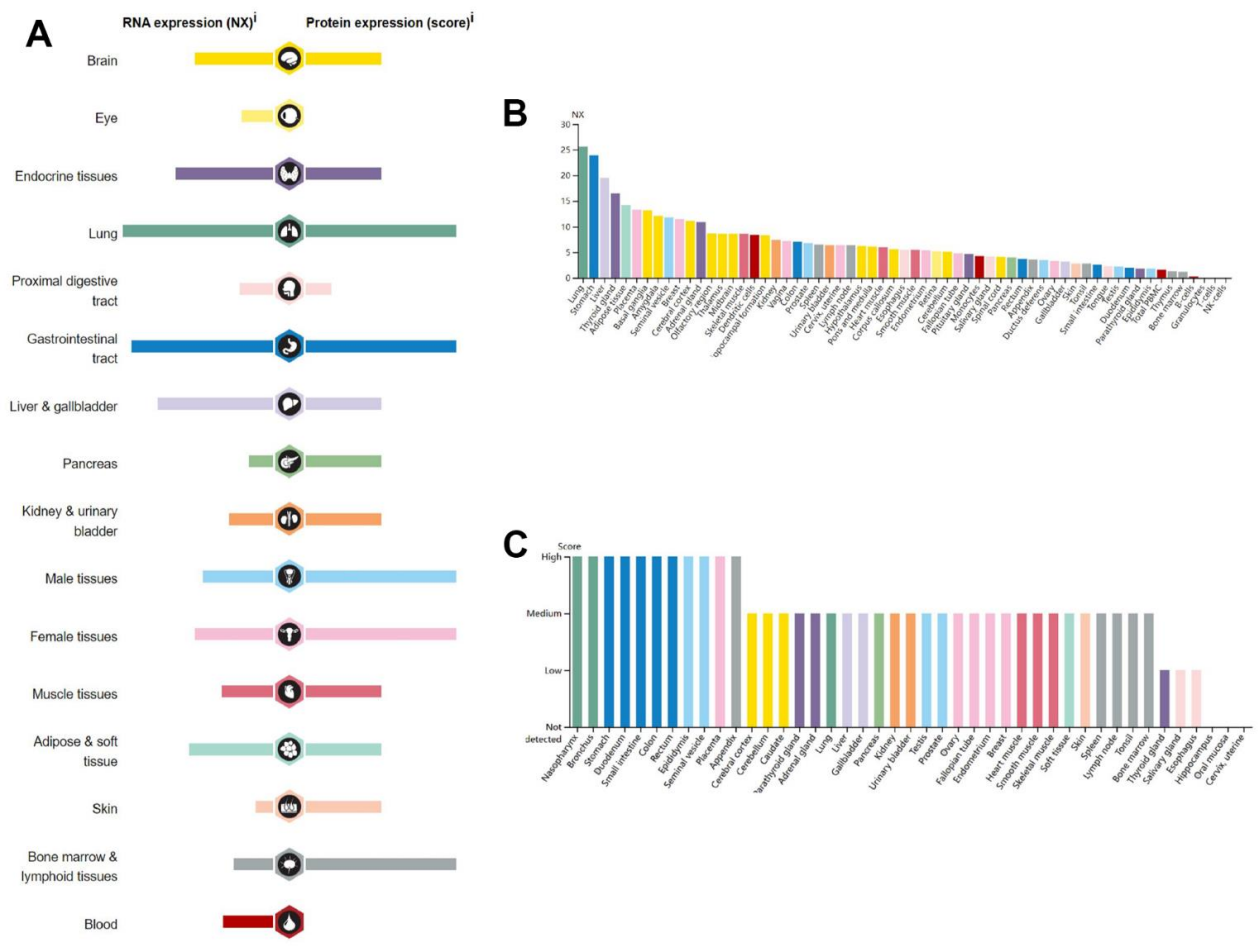
**RNA and protein expression profile for *GPER1* in different human organs and tissues present by HPA.** (**A**) *GPER1* RNA and protein expression summary in different human organs and tissues; Summary of RNA and protein expression information produced within the Human Protein Atlas initiative. Examined tissues are categorized into groups with color-coded distinctions based on shared functional attributes. (**B**) *GPER1* RNA expression summary in different human organs and tissues based on consensus dataset; The unified dataset comprises normalized expression (nTPM) levels for 55 distinct tissue types, achieved through the integration of HPA and GTEx transcriptomics datasets via an internal normalization process. The utilization of color codes corresponds to tissue groupings, with each group comprising tissues sharing common functional attributes. (**C**) *GPER1* protein expression summary in different human organs and tissues. For every one of the 44 tissues, protein expression information is displayed. Color classification is rooted in tissue groups, where each group is composed of tissues that share common functional characteristics.

According to the results obtained from the HPA database, *GPER1* mRNA and protein are widely expressed in various organs and tissues throughout the body (Figure 1A). *GPER1* mRNA is expressed primarily in the lungs, stomach, liver, thyroid, adipose tissue, placenta, basal ganglia, amygdala, seminal vesicles, breast, cerebral cortex and adrenal glands (Figure 1B, 1C).

**Figure 2:**
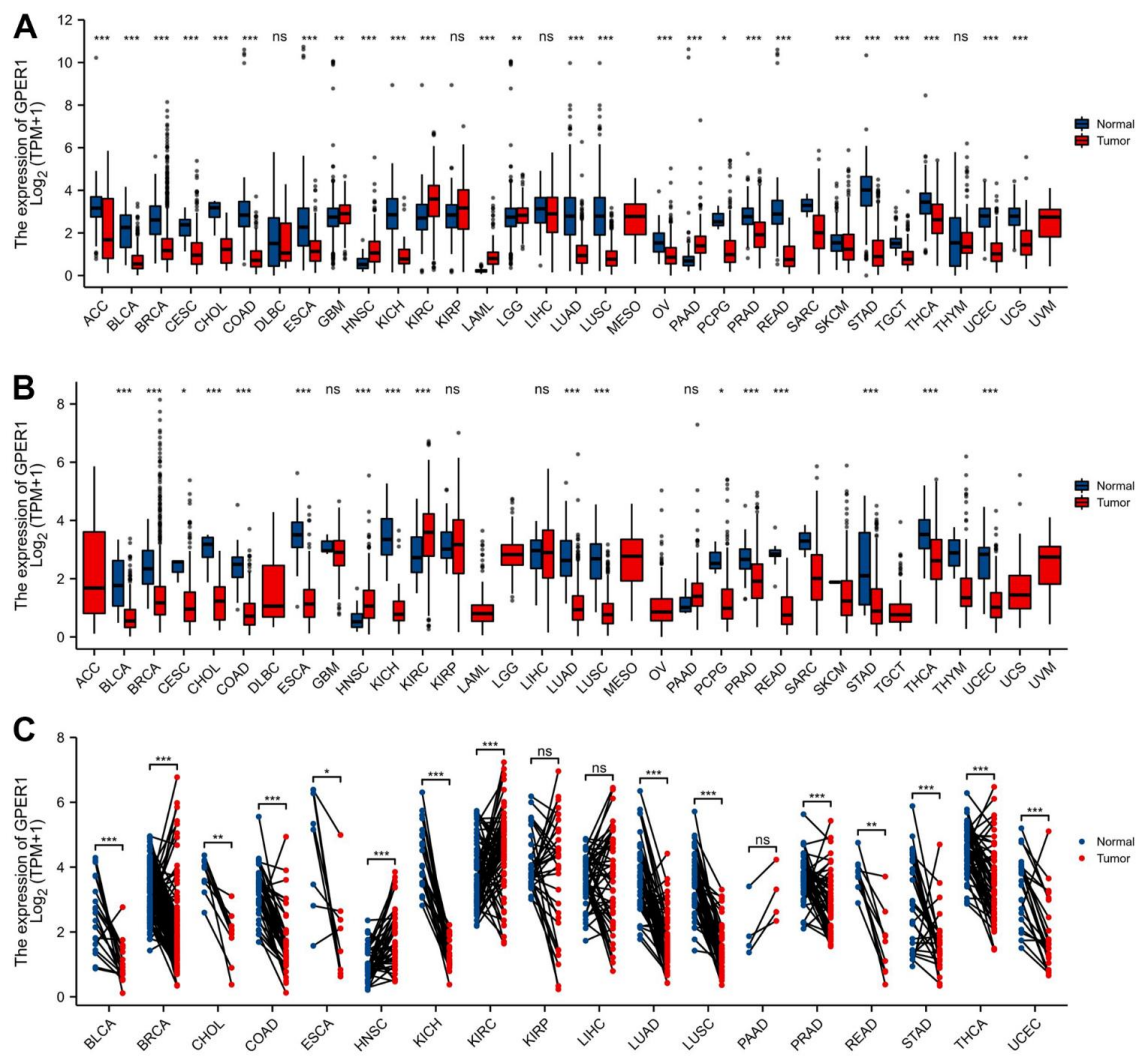
**The expression of *GPER1* mRNA in pan-cancer.** (**A**) Pan-cancer expression of *GPER1* between tumor and normal tissues in unpaired sample analysis; (**B**) Pan-cancer expression of *GPER1* between tumor and paracancerous tissue in unpaired sample analysis; Based on publicly available data, molecular distinctions across diverse pan-cancer datasets are directly analyzed to perform comparative analysis between the tumor group and the normal (adjacent) group. (**C**) Paired sample analysis of *GPER1* between tumor and normal tissues in BLCA, BRCA, CHOL, COAD, ESCA, HNSC, KICH, KIRC, KIRP, LIHC, LUAD, LUSC, PAAD, PRAD, READ, STAD, THCA and UCEC. Each line represents a paired sample, namely the normal (adjacent) versus tumor samples selected from the available public data. The more consistent and inclined the trend direction of the lines, the more pronounced the differences between the two groups. Wilcoxon rank sum test * p < 0:05, ** p < 0:01, *** p < 0:001.

*GPER1* mRNA expression was evaluated in pan-cancer including Adrenocortical carcinoma (ACC), Bladder Urothelial Carcinoma (BLCA), Breast invasive carcinoma (BRCA), Cervical squamous cell carcinoma and endocervical adenocarcinoma (CESC), Cholangiocarcinoma (CHOL), Colon adenocarcinoma (COAD), Lymphoid Neoplasm Diffuse Large B-cell Lymphoma (DLBC), Esophageal carcinoma (ESCA), Glioblastoma multiforme (GBM), Head and Neck squamous cell carcinoma (HNSC), Kidney Chromophobe (KICH), Kidney renal clear cell carcinoma (KIRC), Kidney renal papillary cell carcinoma (KIRP), Acute Myeloid Leukemia (LAML), Brain Lower Grade Glioma (LGG), Liver hepatocellular carcinoma (LIHC), Lung adenocarcinoma (LUAD), Lung squamous cell carcinoma (LUSC), Mesothelioma (MESO), Ovarian serous cystadenocarcinoma (OV), Pancreatic adenocarcinoma (PAAD), Pheochromocytoma and Paraganglioma (PCPG), Prostate adenocarcinoma (PRAD), Rectum adenocarcinoma (READ), Sarcoma (SARC), Skin Cutaneous Melanoma (SKCM), Stomach adenocarcinoma (STAD), Testicular Germ Cell Tumors (TGCT), Thyroid carcinoma (THCA), Thymoma (THYM), Uterine Corpus Endometrial Carcinoma (UCEC), Uterine Carcinosarcoma (UCS), and Uveal Melanoma (UVM). As shown in Figure 2A, unpaired sample analysis found that compared to normal samples, *GPER1* mRNA expression was higher in GBM (P = 0.009), LGG (P = 0.002), HNSC, KIRC, LAML and PAAD (all P < 0.001) and lower in ACC, BLCA, BRCA, CESC, CHOL, COAD, ESCA, KICH, LUAD, LUSC, OV, PRAD, READ, PAN-CNACER, STAD, TGCT, THCA, UCEC, UCS (all P < 0.001) and PCPG (P = 0.015). MESO and UVM could not be analysed due to insufficient normal samples. Compared to paracancerous tissue, *GPER1* mRNA expressed was significant higher in HNSC and KIRC (both P < 0.001) and significant lower in BLCA, BRCA, CHOL, COAD, ESCA, KICH, LUAD, LUSC, PRAD, READ, STAD, THCA, UCEC (all P < 0.001), CESC (P = 0.023) and PCPG (P = 0.015) (Figure 2B). ACC, DLBC, LAML, LGG, OV, TGCT, UCS, MESO and UVM could not be analysed due to insufficient paracancerous samples. Among the paired sample analyses that can be performed, *GPER1* mRNA expression was increased in HNSC and KIRC (both P < 0.001) but was decreased in BLCA, BRCA, COAD, KICH, LUAD, LUSC, PRAD, STAD, THCA, UCEC (all P < 0.001), CHOL (P = 0.004), ESCA (P = 0.023) and READ (P = 0.004) (Figure 2C).

### The diagnostic value of *GPER1* in pan-cancer

**Figure 3:**
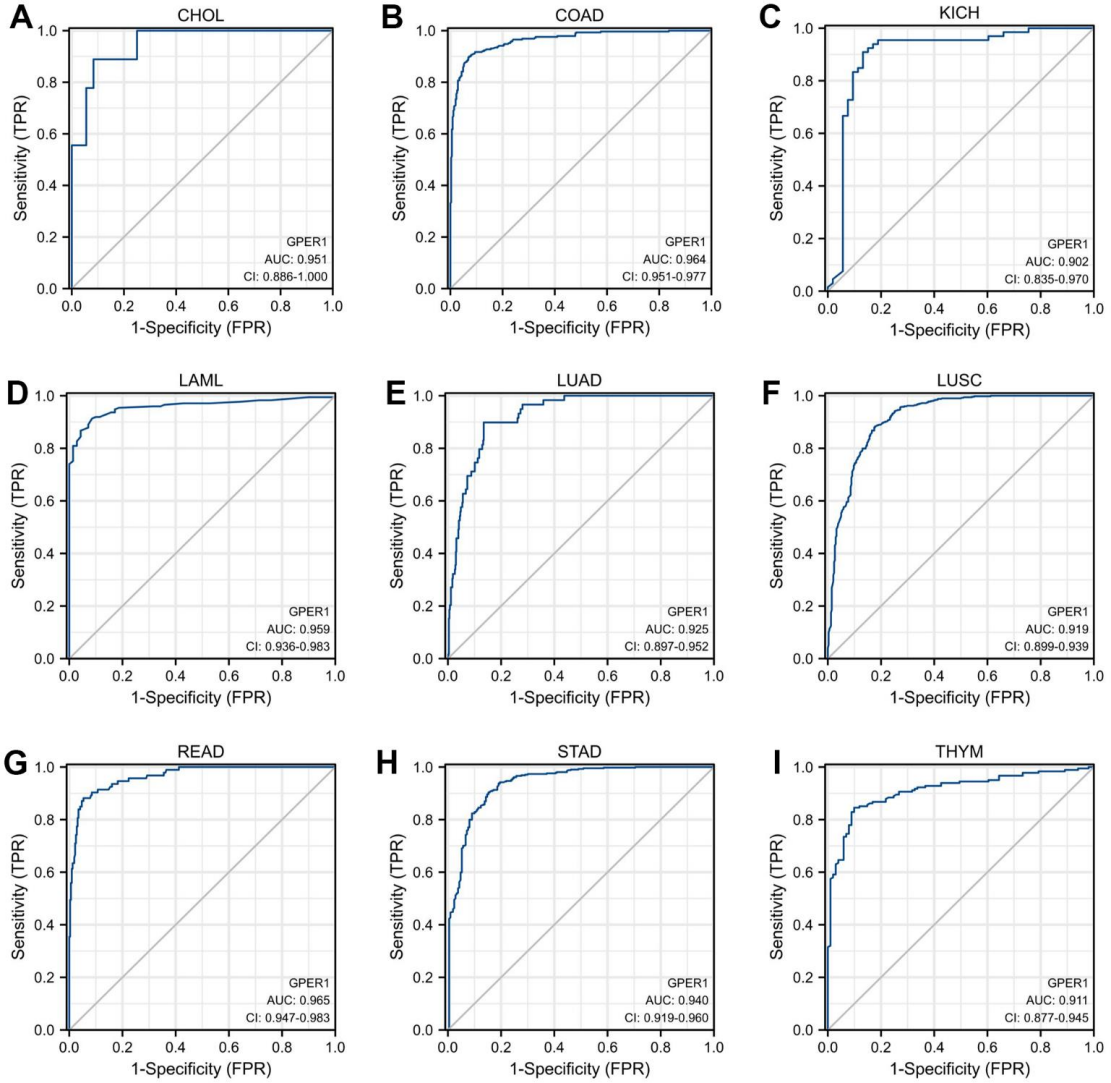
**Receiver operator characteristic (ROC) curve of *GPER1* in cancers.** Cancers with AUC > 0.9 for *GPER1*: (**A**) CHOL; (**B**) COAD; (**C**) KICH; (**D**) LAML; (**E**) LUAD; (**F**) LUSC; (**G**) READ; (**H**) STAD; (**I**) THYM.

As shown in Figure 3, *GPER1* had good diagnostic value in various cancers. Its Area under Curve (AUC) was greater than 0.7 in 21 cancers and even exceeded 0.9 in 9 cancers, including CHOL, COAD, KICH, LAML, LUAD, LUSC, READ, STAD and THYM (Supplementary Table 1), which had high diagnostic value.

### Survival analysis of *GPER1* in pan-cancer

**Figure 4:**
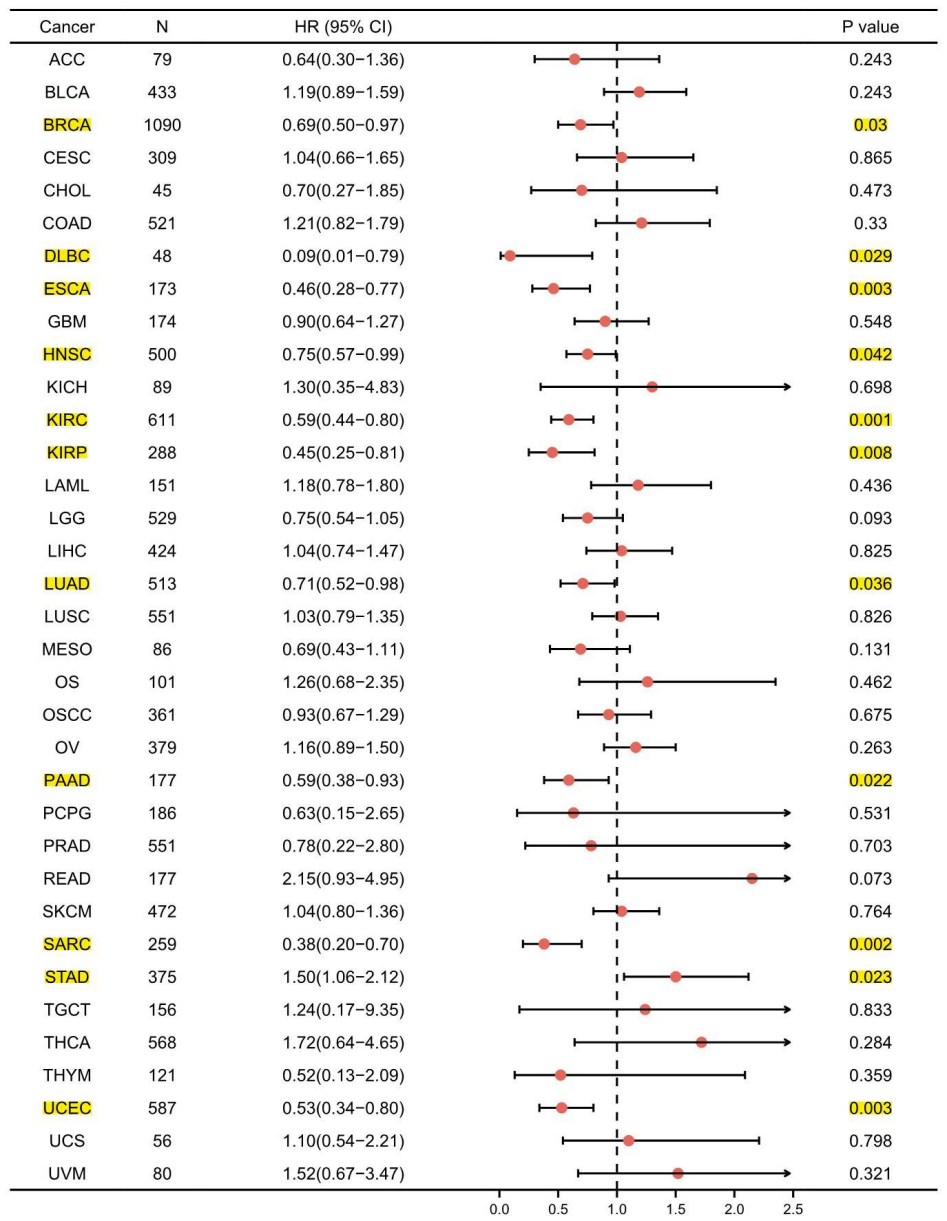
**Forest plot of *GPER1* OS in 35 cancer types.** The marked yellow cancer species indicated that the p-value of prognostic K-M analysis for high- and low- *GPER1* gene expression in the cancer species (BRCA, DLBC, ESCA, HNSC, KIRC, KIRP, LUAD, PAAD, SARC, STAD, UCEC) were less than 0.05.

**Figure 5:**
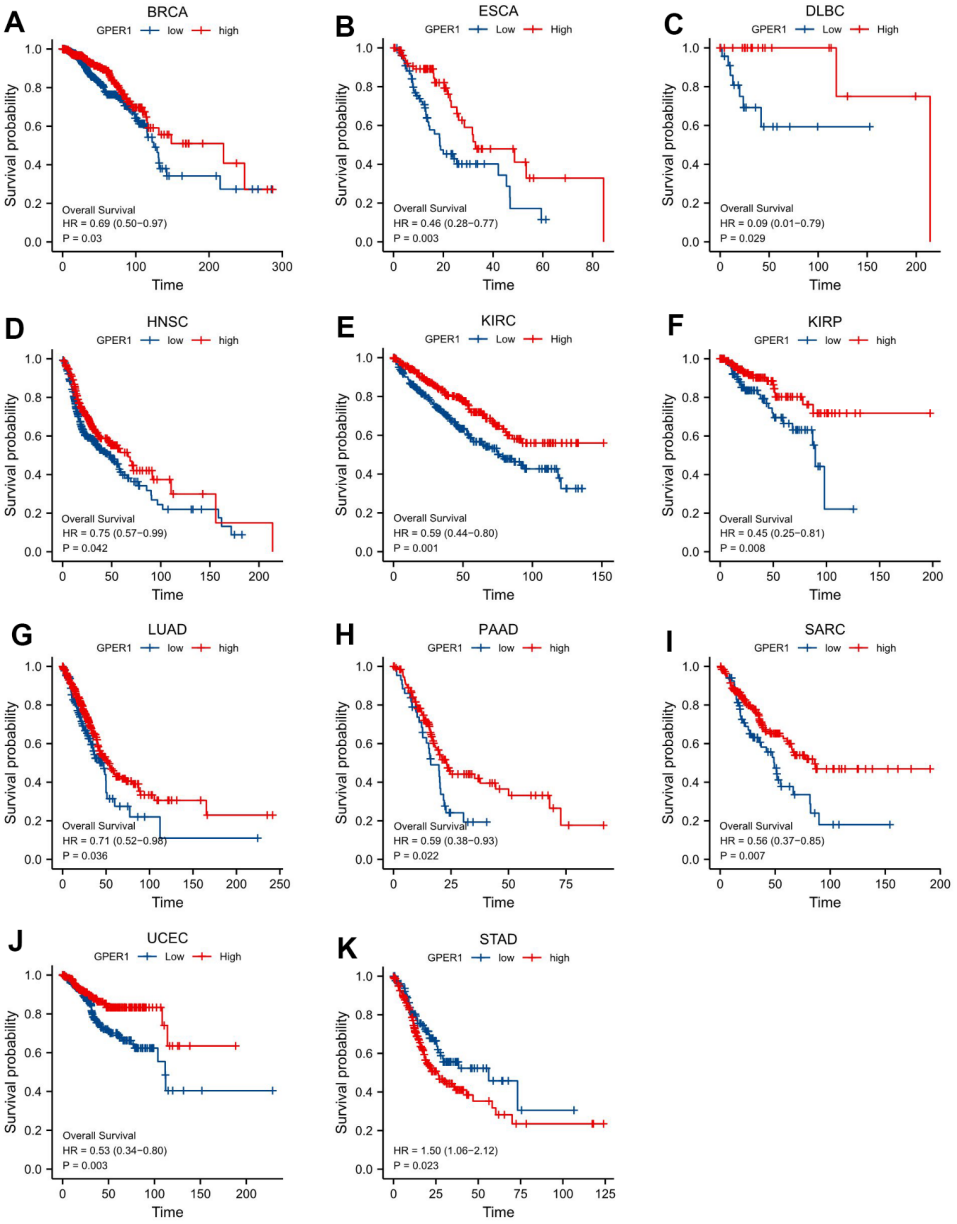
**Correlations between *GPER1* and prognosis in 11 cancer types.** OS K-M curve for *GPER1* 11 cancer types. The unit of X-axis is month. (**A**) BRCA, (**B**) ESCA, (**C**) DLBC, (**D**) HNSC, (**E**) KIRC, (**F**) KIRP, (**G**) LUAD, (**H**) PAAD, (**I**) SARC, (**J**) UCEC, (**K**) STAD.

For the purpose of evaluating the prognostic value of *GPER1* in pan-cancer, Kaplan–Meier (K-M) analysis was conducted. Cox regression analysis of 35 cancers showed that *GPER1* expression in 11 cancers was significantly associated with OS (Supplementary Table 2). Our results found that the high *GPER1* group had significantly better overall survival (OS) than the low *GPER1* group in BRCA (Hazard ratio (HR) 0.69, 95% Confidence Interval (CI): 0.50–0.97; p = 0.03), DLBC (HR 0.09, 95% CI: 0.01–0.79; p = 0.029), ESCA (HR 0.46, 95% CI: 0.28–0.77; p = 0.003), HNSC (HR 0.75, 95% CI: 0.57–0.99; p = 0.042), KIRC (HR 0.59, 95% CI: 0.44–0.80; p = 0.001), KIRP (HR 0.45, 95% CI: 0.25–0.81; p = 0.008), LUAD (HR 0.71, 95% CI: 0.52–0.98; p = 0.036), PAAD (HR 0.59, 95% CI: 0.38–0.93; p = 0.022), SARC (HR 0.38, 95% CI: 0.20–0.70; p = 0.002) and UCEC (HR 0.53, 95% CI: 0.34–0.80; p = 0.003) (Figures 4, 5A–5J). However, the low *GPER1* group showed significantly better OS than the high *GPER1* group in STAD (HR 1.50, 95% CI: 1.06–2.12; p = 0.023) (Figures 4, 5K).

### Genetic alteration of *GPER1* in pan-cancer

**Figure 6:**
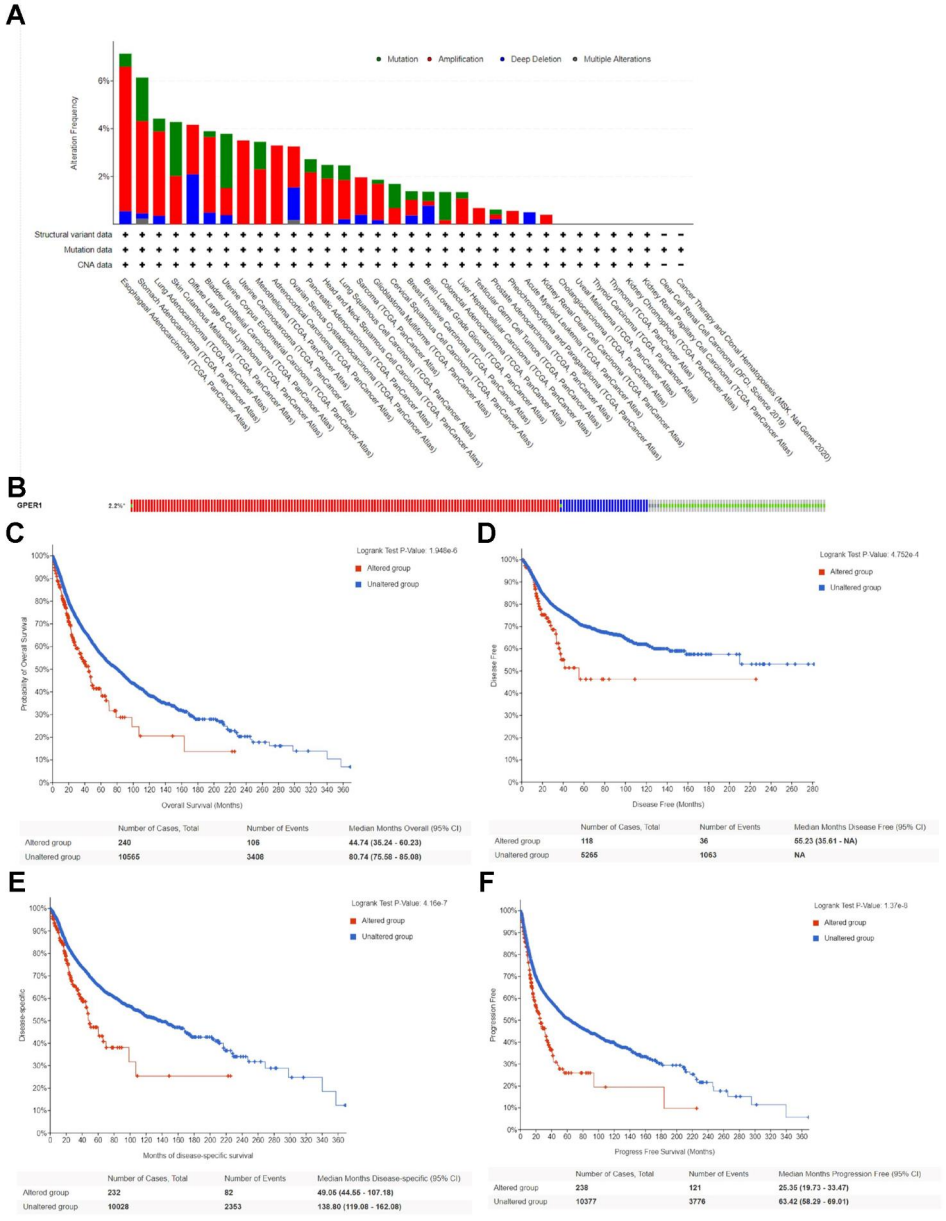
**Genetic alteration of *GPER1* in pan-cancer.** (**A**) Bar chart of *GPER1* mutation in pan-cancer based on TCGA database. (**B**) The alteration frequency with different types of *GPER1* gene mutations in pan-cancer. Kaplan-Meier curve of (**C**) OS, (**D**) DSS, (**E**) DFS, (**F**) PFS in pan-cancer patients with altered (red) and unaltered (blue) mRNA expression of the *GPER1* gene.

This study analysed genetic mutations of *GPER1* in pan-cancer using the cBioPortal online tool. Based on TCGA, *GPER1* mutations were most commonly seen in ESCA, STAD, LUAD, SKCM, DLBC, BLCA and UCEC (Figure 6A). The mutation rate of *GPER1* genes was 2.2%, with the most predominant mutation types being Amplification, Deep Deletion and Missense mutation (Figure 6B). The correlation between genetic mutations and prognosis of pan-cancer patients was further explored. Accordingly, *GPER1* genetic mutations promoted a significant decrease in OS (Figure 6C), disease-free survival (DFS) (Figure 6D), disease-specific survival (DSS) (Figure 6E) and progression-free survival (PFS) (Figure 6F) (all p < 0.001) in pan-cancer.

### DEGs, PPI, functional enrichment and gene set enrichment of *GPER1* in cancers

Earlier, we showed that *GPER1* expression affected the OS from 11 cancers. To evaluate the biological function of *GPER1* in specific cancers, we then analysed differential genes for high and low expression of *GPER1* in these cancers and constructed PPI networks with the top 30 up- or downregulated DEGs, as well as performing functional enrichment and gene set enrichment analyses. By analyzing the standardized DEGs in each specific cancer type, the counts of DEGs identified were as follows: BRCA (1117), DLBC (739), ESCA (671), HNSC (837), KIRC (4876), KIRP (2380), LUAD (3960), PAAD (470), SARC (1309), STAD (2228), UCEC (1020). All the DEGs were list in Supplementary File 1.

**Figure 7:**
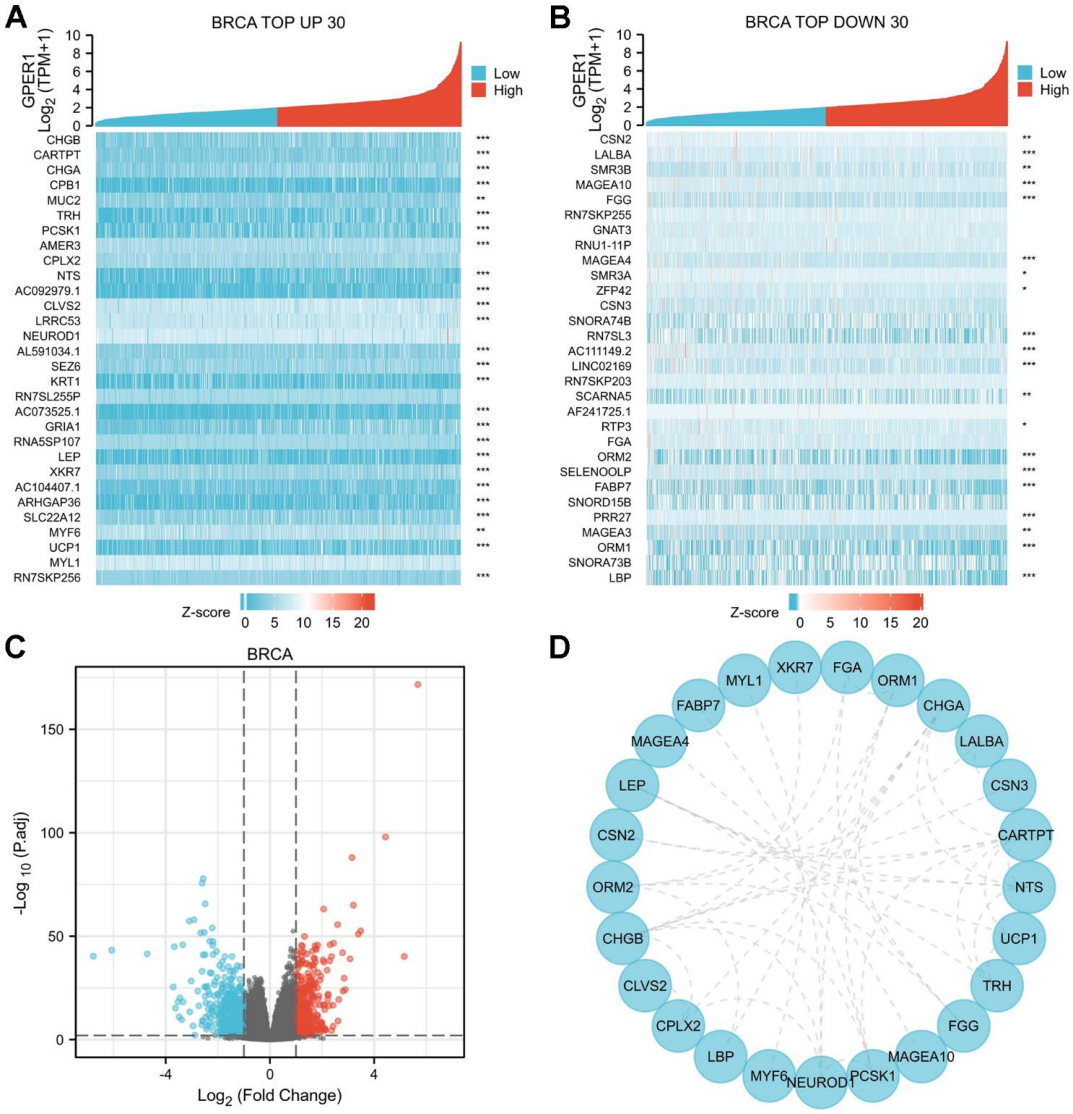
**DEGs of high and low *GPER1* expression in BRCA and PPI network of DEGs.** (**A**) The heatmap of top 30 up-regulated DEGs, (**B**) The heatmap of top 30 down-regulated DEGs. Each square represents the expression value of other molecules after undergoing Z-score transformation across various samples (Z-score involves subtracting the mean expression value of each molecule in individual samples from its mean expression value across all samples and then dividing by the standard deviation), with color intensity indicating the absolute value of the expression level. (**C**) The volcano plots of DEGs between high and low *GPER1* expression groups, (**D**) PPI network of DEGs of high and low *GPER1* expression in BRCA. * *p* < 0.05, ** *p* < 0.01, *** *p* < 0.001. The “*p*” value represents the *p*-value obtained from the Spearman test conducted to calculate the correlation coefficient between *GPER1* and the top 30 up- and downregulated genes.

**Figure 8:**
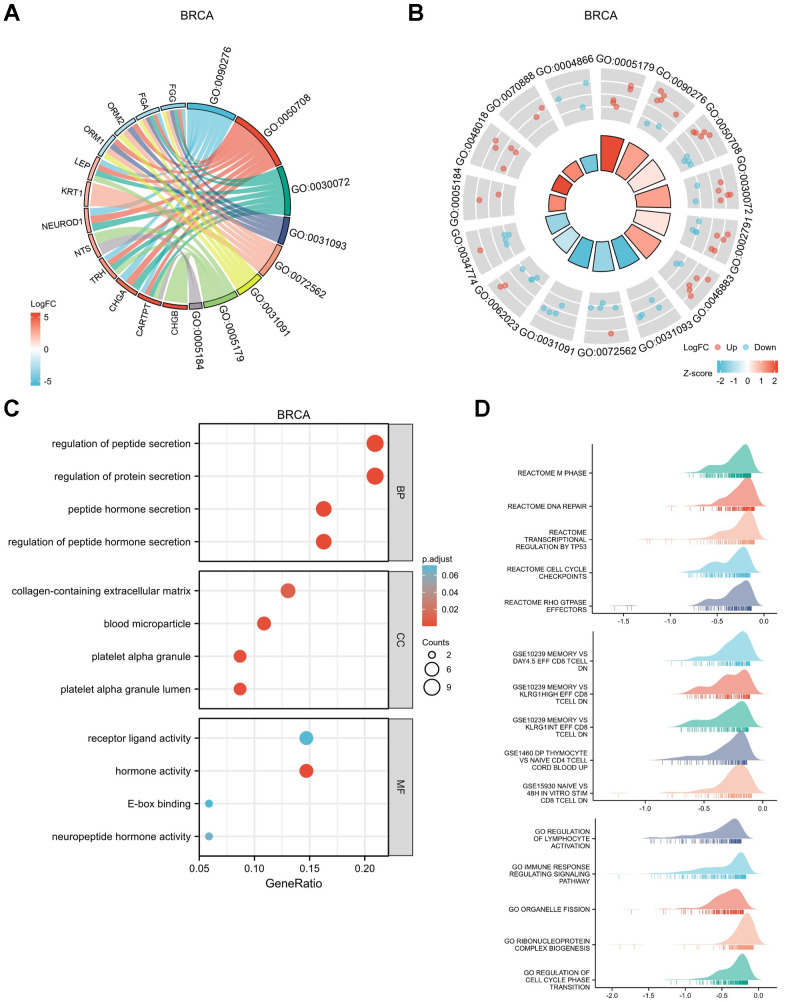
**Functional enrichment analysis for DEGs between High and Low expression of *GPER1* expression in BRCA.** (**A**) GO/KEGG pathway enrichment joint logFC for DEGs between High and -Low expression of *GPER1* expression in BRCA presented as string graph. The left half of the figure shows the gene blocks, and the different colors of the blocks represent the corresponding logFC values. The right half of the graph shows the entry blocks, the size of the blocks represents the corresponding Counts, and the lines (strings) between the blocks on the left and right half of the graph represent the molecules contained in the entry, the presence of the lines means that the entry contains the corresponding molecules. (**B**) GO/KEGG joint logFC results presented as circle graph. The circle diagram can be divided into two parts: the inner circle and the outer circle. Each bar in the inner circle corresponds to an entry, and the height is the relative size of the p.adj. The higher the bar, the smaller the p.adj of the ID. The color of the corresponding filled column represents the Zscore value of the entry. (**C**) GO/KEGG pathway enrichment presented as bubble chart, (**D**) GSEA of the signaling pathways associated with DEGs of *GPER1* expression in BRCA.

Our results were presented in a form similar to that in BRCA (Figures 7, 8). The top 30 up- or downregulated DEGs of *GPER1* in BRCA are summarised in Figure 7A, 7B, respectively (Supplementary Table 3). The DEGs of *GPER1* in BRCA were presented as volcano plots (Figure 7C). Figure 7D presents the PPI networks of the top 30 up- or downregulated DEGs (Supplementary Table 4).

The top 30 up- or downregulated DEGs were used to perform Gene Ontology (GO) / Kyoto Encyclopedia of Genes and Genomes (KEGG) joint logFC analyses (Supplementary Table 5). The results were presented as string (Figure 8A) and circle graphs (Figure 8B). The different RNA functions of DEGs can be divided into three categories: biological process (BP), molecular function (MF) and cellular component (CC). The top three GO terms for the BP in BRCA included regulation of peptide hormone secretion, regulation of protein secretion and peptide hormone secretion; those for CC included platelet alpha granule lumen, blood microparticle and platelet alpha granule; and those for MF included hormone activity and neuropeptide hormone activity. As shown in the bubble chart demonstrating the results for GO/KEGG analyses (Figure 8C and Supplementary Table 6), the top BP terms in BRCA included regulation of peptide hormone secretion, peptide hormone secretion, regulation of hormone secretion and signal release; those for CC included mast cell granule, axon terminus and neuron projection terminus; and those for MF included hormone activity and neuropeptide hormone activity.

Figure 8D shows the GSEA results for DEGs in BRCA. The top five enrichments in biological pathways were REACTOME M PHASE, REACTOME DNA REPAIR, REACTOME TRANSCRIPTIONAL REGULATION BY TP53, REACTOME CELL CYCLE CHECKPOINTS and REACTOME RHO GTPASE EFFECTORS. The top five enrichments in GO were REGULATION OF LYMPHOCYTE ACTIVATION, IMMUNE RESPONSE REGULATING SIGNALLING PATHWAY, ORGANELLE FISSION, RIBONUCLEOPROTEIN COMPLEX BIOGENESIS and REGULATION OF CELL CYCLE PHASE TRANSITION. The top five enrichments in immunologic signatures were GSE10239 MEMORY VS DAY4.5 EFF, CD8 TCELL DN, GSE10239 MEMORY VS KLRG1HIGH EFF CD8 TCELL DN, GSE10239 MEMORY VS KLRG1INT EFF CD8 TCELL DN, GSE1460 DP THYMOCYTE VS NAIVE CD4 TCELL CORD BLOOD UP and GSE15930 NAIVE VS 48H *IN VITRO* STIM CD8 TCELL DN.

The results for other cancers, including DLBC (Supplementary Figures 1, 2), ESCA (Supplementary Figures 3, 4), HNSC (Supplementary Figures 5, 6), KIRC (Supplementary Figures 7, 8), KIRP (Supplementary Figures 9, 10), LUAD (Supplementary Figures 11, 12), PAAD (Supplementary Figures 13, 14), SARC (Supplementary Figures 15, 16), STAD (Supplementary Figures 17, 18) and UCEC (Supplementary Figures 19, 20), were presented in a form similar to that for BRCA.

### Methylation level of *GPER1* in cancers

Gene methylation is closely associated with the development and progression of several cancers. This study obtained the methylation data of *GPER1*, which have been considered significant in the survival analysis with corresponding normal tissues, for 10 cancers using the MethSurv database. The database does not include information on the methylation of *GPER1* in DLBC. The methylation information of *GPER1* for 10 cancers were presented as heatmaps in Supplementary Figure 21A–21J.

**Figure 9:**
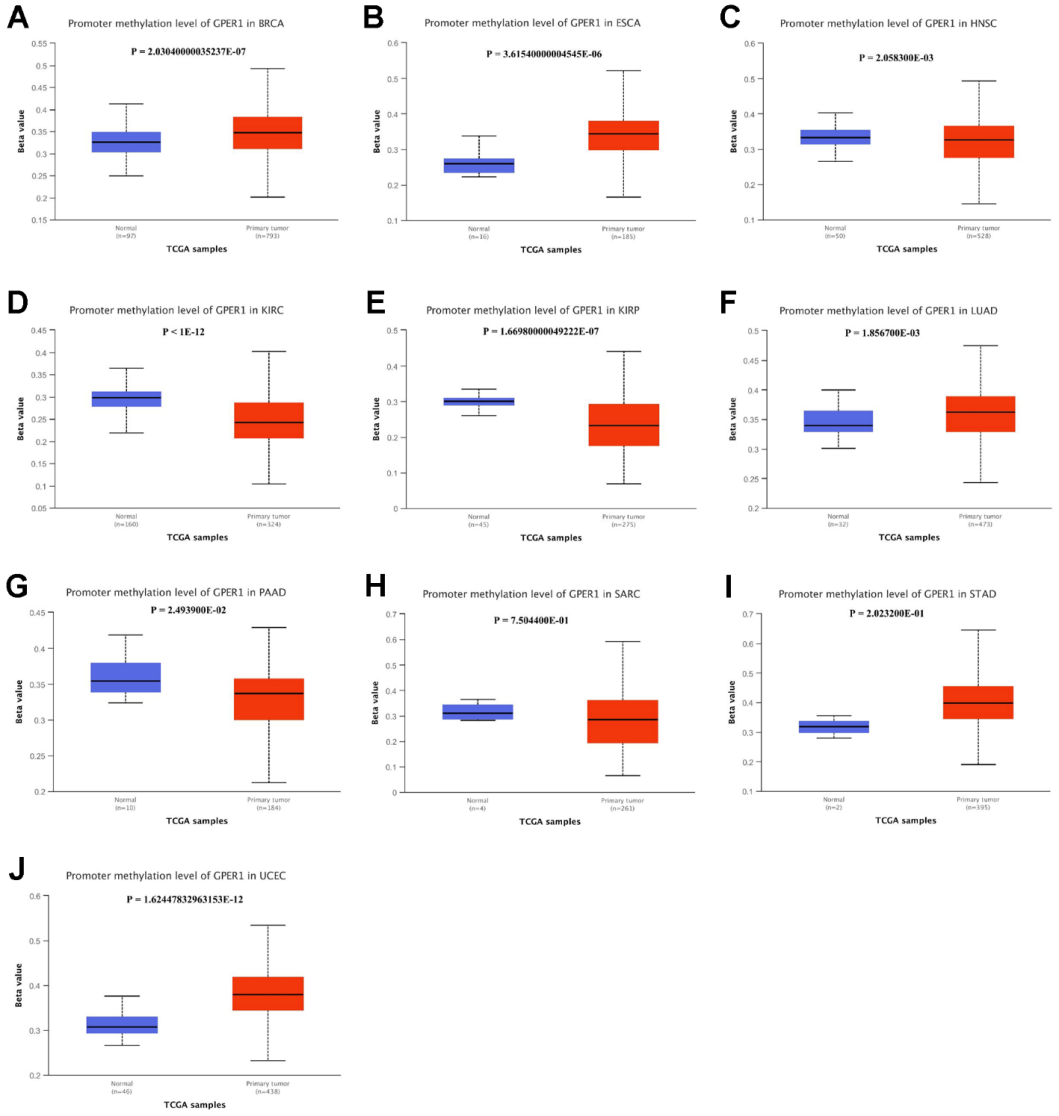
**Promoter methylation level of *GPER1* between 10 types of cancer and normal tissue.** (**A**) BRCA, (**B**) ESCA, (**C**) HNSC, (**D**) KIRC, (**E**) KIRP, (**F**) LUAD, (**G**) PAAD, (**H**) SARC, (**I**) STAD, (**J**) UCEC.

We further compared the *GPER1* methylation levels among the identified cancers. Except for DLBC, methylation data of *GPER1* for 10 cancers and normal tissues were obtained. Our findings showed that the methylation level of *GPER1* was significantly higher in BRCA (p < 0.001), ESCA (p < 0.001), HNSC (p < 0.05), LUAD (p < 0.005) and UCEC (p < 0.001) compared to that in normal tissues. In contrast, the methylation level of *GPER1* was significantly lower in KIRC (p < 0.001), KIRP (p < 0.001) and PAAD (p < 0.05) compared to that in normal tissues. No differences were found between SARC and STAD tissues and corresponding normal tissues (Figure 9A–9J).

### Pan-cancer immunogenomic analyses of *GPER1*

**Figure 10:**
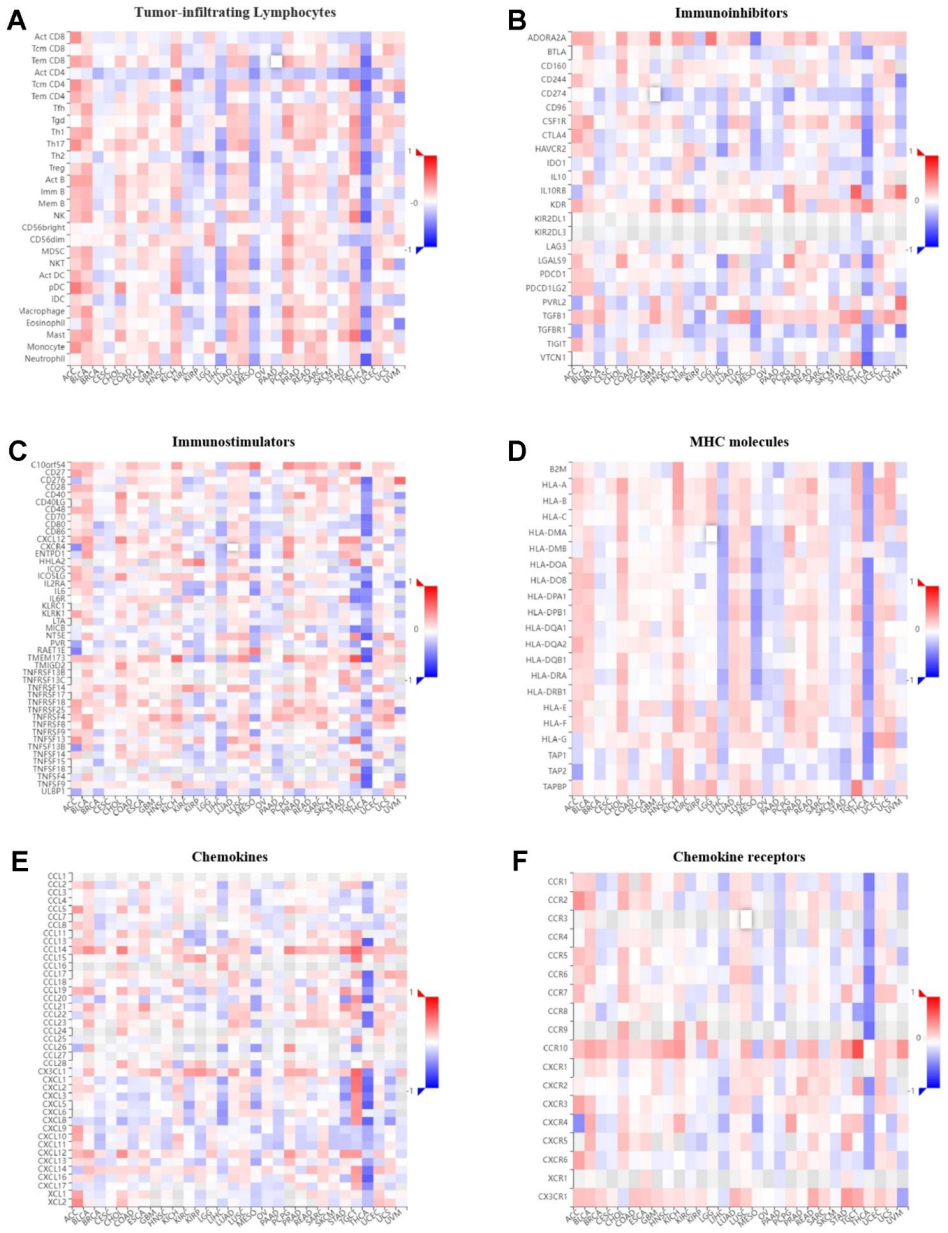
**Correlation of *GPER1* with TILs and immunoregulation-related genes in pan-cancers.** Correlations between *GPER1* and (**A**) TILs, (**B**) immunoinhibitors, (**C**) immunostimulators, (**D**) MHC molecules, (**E**) Chemokines, (**F**) Chemokine receptors.

**Figure 11:**
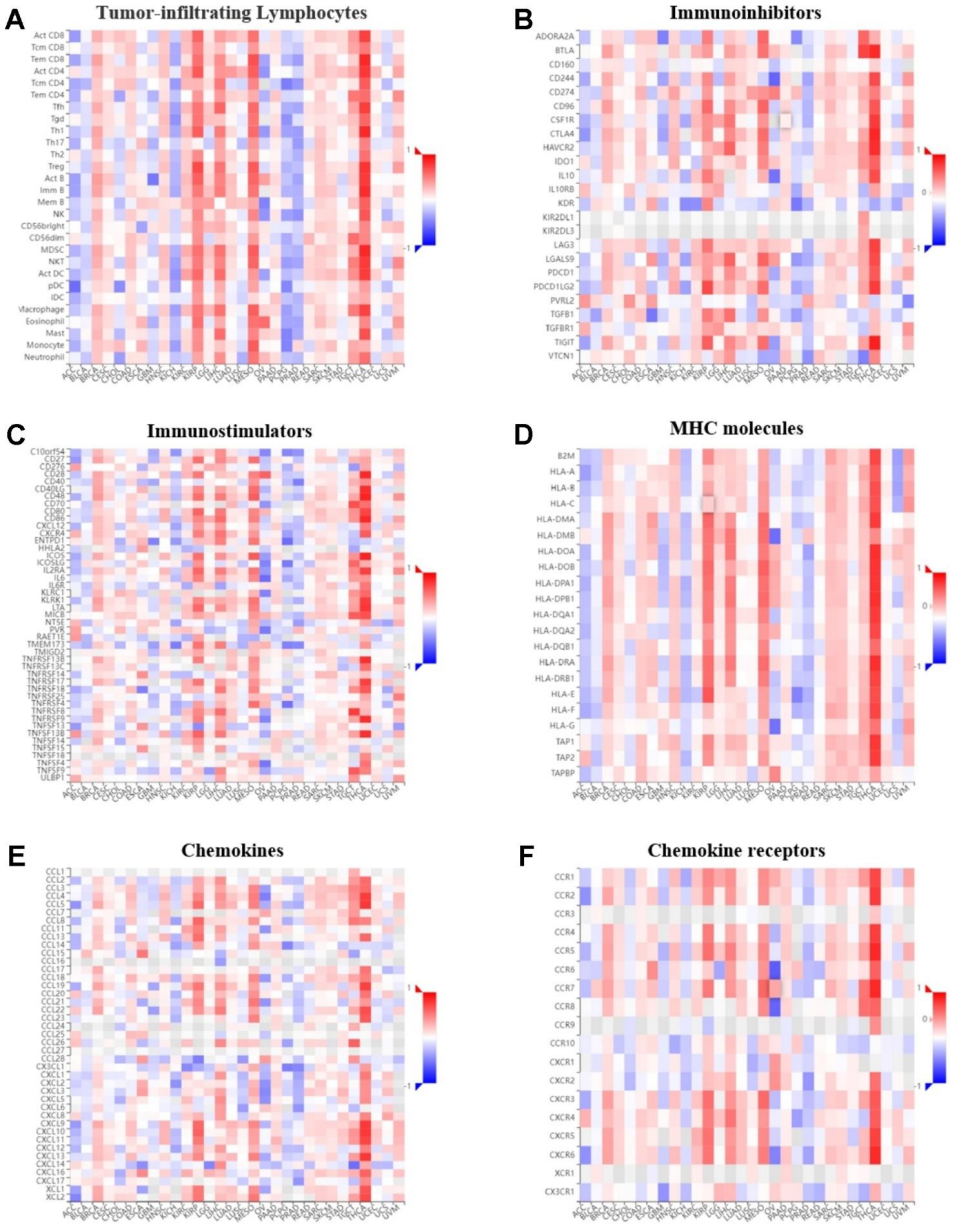
**Correlation of *GPER1* DNA methylation with TILs and immunoregulation-related genes in pan-cancers.** Correlations between *GPER1* DNA methylation and (**A**) TILs, (**B**) immunoinhibitors, (**C**) immunostimulators, (**D**) MHC molecules, (**E**) Chemokines, (**F**) Chemokine receptors.

The correlation between *GPER1* expression level and immune components in pan-cancer were inferred via the TISIDB database. The relationship between *GPER1* and TILs (Figure 10A), immunoinhibitors (Figure 10B), immunostimulators (Figure 10C), MHC molecules (Figure 10D), chemokines (Figure 10E) and chemokine receptors (Figure 10F), as well as that between methylation level of *GPER1* and immune components (Figure 11A–11F), were presented as heatmaps.

Our findings showed that *GPER1* expression was positively correlated with TILs in majority cancers. However, we found that *GPER1* expression was negatively correlated with TILs in KIRC, KIRP, LIHC, MESO, PAAD, SKCM and THCA. Of interest was the negative correlation between *GPER1* and act CD4 in most cancers. Our results also found that *GPER1* expression was negatively correlated with TILs, immunoinhibitors, immunostimulators, MHC molecules, chemokines and chemokine receptors in LIHC, MESO, PAAD and THCA. In particular, the negative correlation in THCA was greater than that in other cancers. The correlation between *GPER1* methylation and immune components differed from that between *GPER1* and immune components. The positive correlation in ACC, BLCA, KICH, PCPG and PRAD turned out to be a negative correlation, whereas the negative correlation in KIRP, LIHC, MESO and THCA turned out to be a positive correlation.

## DISCUSSION

Cancer, a leading cause of global mortality, demands improved prevention and treatment. Tumor biomarkers offer versatile clinical applications. Estrogen’s roles extend beyond reproduction, impacting physiology and carcinogenesis through regulating apoptosis, proliferation, and tumor microenvironment interactions. *GPER1*, alongside classical estrogen receptors, is expressed widely, mediating rapid estrogen effects across various systems. It affects tumor processes, highlighting its therapeutic potential in cancer. However, comprehensive pan-cancer analysis of *GPER1* remains lacking. This study comprehensively investigated the multifaceted role of *GPER1* across various cancers. The study delved into *GPER1*’s expression, diagnostic potential, survival implications, epigenetic regulation, genetic alterations, functional significance, and immunogenomic interactions. *GPER1*’s mRNA and protein were found to be widely expressed in diverse tissues, forming the basis for pan-cancer analyses. These analyses unveiled its diagnostic potential, as elevated expression was observed in specific cancers such as GBM, LGG, and HNSC, while decreased expression characterized others. Interestingly, *GPER1* expression correlated with diverse overall survival outcomes in distinct cancer types. Furthermore, the study explored *GPER1*’s epigenetic landscape, highlighting methylation patterns that exhibited heterogeneity across cancers, contributing to its regulatory complexity. Genetic mutations, encompassing amplifications and deletions, were associated with distinct survival profiles. Functional analyses provided insights into *GPER1*’s potential roles in various pathways. The investigation extended to *GPER1*’s intricate relationship with the immune microenvironment, revealing both positive and negative correlations with immune components in different cancers.

Timely diagnosis holds paramount importance in the realm of cancer prevention and treatment. Furthermore, the correlation between early diagnosis and prompt therapeutic intervention has been well-established, demonstrating a substantial enhancement in the survival rates across various malignancies [6]. Therefore, identifying tumour markers with high diagnostic value is imperative. Our results showed that *GPER1* is widely expressed in various organs and tissues, both in mRNA and protein. Furthermore, the expression of *GPER1* differs significantly between normal or paracancerous tissues in numerous cancers, providing easy access to samples for clinical diagnosis. Our findings also showed that the AUC of *GPER1* Receiver operator characteristic (ROC) was more than 0.7 in 21 cancers, indicating that *GPER1* had a wide diagnostic efficacy in cancer. Notably, its AUC exceeded 0.9 in nine cancers, suggesting that *GPER1* has great detective ability and reliable efficiency, allowing its use as a diagnostic biomarker in these cancers.

*GPER1* is involved in regulating various tumours, such as breast, pancreatic, oesophageal, endometrial, ovarian, cervical, prostate and testicular cancers, as well as lung, liver, thyroid, colorectal and kidney cancers [13]. Although an increasing number of studies have focused on the role of *GPER1* in different types of cancers, it remains controversial whether *GPER1* plays a pro- or anti-cancer role in tumours. Several studies have shown that activation of *GPER1* can promote carcinogenesis, whereas others have shown that its activation can suppress tumours (reviewed in [14]). Neither *ex vivo* nor *in vivo* experiments have so far led to definitive conclusions. Our results showed that low *GPER1* expression was associated with poor prognosis in BRCA, DLBC, ESCA, HNSC, KIRC, KIRP, LUAD, PAAD, SARC and UCEC, whereas high *GPER1* expression was associated with poor prognosis in STAD. Evidence has suggested that *GPER1* may be a prognostic predictive marker for these cancers. Although our results had been derived from the aggregation of multiple studies’ samples in the TCGA database, the limited number of individual study samples may explain the inconsistency within our results derived from individual studies. Moreover, the results of some experiments had been derived from cell lines that may differ from the results of the primary tumour.

*GPER1* plays an important biological role in regulating oestrogenic responses in breast malignancies and has been associated with increased tumour size, increased risk of recurrence and metastasis, decreased survival and therapy resistance in breast cancer patients [17]. However, additional studies have reported that *GPER1* inhibits breast cancer proliferation, progression and tumour angiogenesis [18–20]. Study concerning endometrial cancer had found that *GPER1* expression was reduced in endometrial cancer cell lines, which is consistent with our results [21]. The *GPER1* agonist G1 dose-dependently inhibited the growth of *GPER1*-positive cell lines RL95-2 and HEC-1A, whereas the *GPER1*-negative cell line HEC-1B was not affected [21]. This indicates that G1 requires only a moderate amount of *GPER1* to exert growth inhibitory effects. This also suggests that the effects of *GPER1* on tumours may not depend on the amount of expression but rather in the activation. In gastric cancer, *GPER1* promotes gastric cancer proliferation, migration and invasion through PI3K/akt-mediated EMT [22]. This is consistent with our results showing that high *GPER1* levels are associated with poor prognosis in STAD. *GPER1* agonist G1 increased the number of tumour nodules, tumour grade and tumour index in urethane-induced lung adenocarcinoma models [23]. However, another study reported that G1 can mediate anti-proliferative and pro-apoptotic effects of oxidants and antioxidant molecules on A549 cells [24] and that *GPER1* activation can also inhibit migration of human NSCLC cells by suppressing IKK-β/NF-κB signalling [25]. Furthermore, the activation of *GPER1* had also been found to inhibit the migration and invasion of osteosarcoma cells through FBXL5-mediated post-translational downregulation of Snail [26]. In pancreatic cancer, high *GPER1* expression has been associated with improved survival [27], and *GPER1* activation leads to peritumoral mesenchymal remodelling in PDAC, reducing fibrous tissue proliferation, inflammation and immunosuppression [28].

Methylation of gene promoter regions can lead to gene transcriptional repression, and aberrant gene methylation may contribute to oncogenic transformation [29]. Our study findings demonstrate that across the 10 studied cancer types, *GPER1* exhibits high methylation in 5 cancers (BRCA, ESCA, HNSC, LUAD, and UCEC) and low methylation in 3 cancers (KIRC, KIRP, and PAAD). We observe decreased *GPER1* expression in BRCA, ESCA, LUAD, and UCEC compared to normal or adjacent tissues, while it is elevated in KIRC, KIRP, and PAAD, consistent with our methylation analysis results. In HNSC, *GPER1* expression results do not align with methylation outcomes. Methylation analysis reveals minimal differences in median BETA values between HNSC and LUAD, suggesting uncertain biological significance despite potential statistical disparities. This might elucidate the discrepancy between elevated *GPER1* expression and unexpectedly “higher” DNA methylation in HNSC compared to normal tissue. *GPER1* shows no significant differences in methylation compared to normal tissue in two cancers (SARC and STAD). *GPER1* exhibits lower expression in STAD, contradictory to survival analysis indicating better OS in the low *GPER1* subgroup of STAD. This complexity might suggest intricate epigenetic regulation of *GPER1*, with DNA methylation potentially holding a dominant role while histone modifications might also play a crucial role. This also implies the intricate involvement of *GPER1* in cancer development. Whether *GPER1* promotes or suppresses cancer lacks a definite conclusion, as *GPER1* methylation involves a series of subsequent changes, encompassing downstream signaling pathways and immune modulation [13].

Gene mutations affect not only cancer development but also cancer progression. Loss of homozygous ancestral genotype GG is more common in two polymorphisms (rs3808350 and rs3808351) in the *GPER1* promoter region of spermatocytomas but not in non-seminomas [30]. The T allele of the *GPER1* gene SNP rs11544331 triggers the expression of the P16L variant, which promotes the migration of breast cancer cells [31]. Our study found that *GPER1* gene mutations were associated with poor prognosis in patients with tumours. In all samples from patients with tumour, *GPER1* gene mutations decreased OS, DFS, DSS and PFS, suggesting that *GPER1* gene alterations play an important role in cancer progression and that the associated changes in *GPER1* expression levels could provide prognostic value for patients with tumour. However, this also implies that *GPER1* itself plays a key role in the development and progression of several cancers and that mutations can cause failure or alteration of this role.

Genes and proteins associated with differential expression of target genes may be associated with specific biological functions or pathways. Analysis of the associated genes or proteins can help us better determine the mechanism of action of the target genes in the disease. Among the 11 cancers selected for analysis, high or low *GPER1* expression showed a significant effect on OS. Unlike other pan-cancer analyses, our study analysed the target genes in each specific cancer species. Thus, the biological pathways obtained during subsequent enrichment analysis were closely associated with that specific cancer species.

Immune components, including TILs, immune activators, immunosuppressors, MHC, chemokines and chemokine receptors, are important components of tumour immunity. Our results found that *GPER1* was associated with gene expression of these immune components various cancers. In some cancers, *GPER1* was correlated with immune components with some consistency. For instance, *GPER1* was negatively correlated with immune components in LIHC, MESO and THCA, suggesting its involvement in the immune infiltration of these tumours and the composition of the tumour microenvironment. However, this also indicates the complexity of the role played by *GPER1*. The negative or positive correlation between *GPER1* and both immunosuppressive and immunostimulatory factors may explain why no conclusive conclusion can be reached on whether *GPER1* is cancer-promoting or -suppressing despite the numerous studies. This complexity is also compounded by differences in the genetic correlation between *GPER1* and the same immune component among various tumours, suggesting that *GPER1* has different effects on tumour immunity in different cancers. Our results also found that the correlation between *GPER1* and immune component-related genes was altered after methylation. For instance, the negative correlation in LIHC, MESO and THCA changed to a positive correlation, further suggesting that *GPER1* is closely associated with the tumour immune microenvironment and ligand-receptor interactions between lymphocytes and malignant tumour cells, potentially influencing tumour progression and prognosis.

Recent years of research accumulation have progressively unveiled the multifaceted association between *GPER1* and various aspects of cancer pathogenesis, further accentuating its potential as a therapeutic target for cancer. Consequently, the development of cancer treatment strategies targeting *GPER1* has garnered significant attention [32]. The study by Wegnera et al. [33] revealed that *GPER1* overexpression reduces proliferation and mitochondrial activity in MCF-7 breast cancer cells, concurrently inducing autophagy. However, this also diminishes MCF-7 cell sensitivity to doxorubicin while augmenting the cytotoxic effects of cyclophosphamide. Additionally, the application of fumaric acid ester further enhances the cytotoxic impact of these substances on *GPER1*-overexpressing cells. On a different note, research by Sathya et al. [19] indicated that under low oxygen conditions, estrogen suppresses breast cancer growth via the *GPER1*/ROS/p38 MAPK/p21 signaling pathway. Weißenborn’s findings [34, 35] demonstrated that the *GPER1*-specific agonist G-1 activates *GPER1* in a concentration-dependent manner, effectively inhibiting breast cancer cell growth. This suggests that cell surface-expressed *GPER1* holds promise as a potential therapeutic target for non-triple-negative and triple-negative breast cancers. Recent meta-analysis results [36] correlate elevated *GPER1* mRNA expression with improved survival rates in breast cancer patients. Furthermore, studies have indicated that *GPER1* can inhibit tumor formation and metastasis in cervical cancer cells; reducing *GPER1* expression may strengthen cervical cancer cell stemness and migration/invasion capabilities [37]. Moreover, Xu et al.’s research [22] demonstrated that silencing the *GPER1* gene can inhibit gastric cancer cell proliferation, migration, and invasion by suppressing the PI3K/AKT-mediated EMT process. These research outcomes align with our findings of elevated *GPER1* expression being correlated with adverse prognosis in STAD, indicating its potential as a therapeutic target for gastric cancer treatment [22].

## CONCLUSIONS

In summary, pan-cancer analysis of *GPER1* in our study showed that it was widely expressed in human tissues and organs and that its expression differs from normal tissue in various cancers. The methylation, mutation and mutation-related prognosis of *GPER1* in cancers, the associated pathways in specific cancers and its extensive correlation with immune components suggest that *GPER1* may have a bright future in the diagnosis, and prognosis of multiple tumours, providing new concepts for precise and personalised anti-tumour strategies.

## MATERIALS AND METHODS

### *GPER1* expression and datasets obtained

A summary on *GPER1* RNA and protein expression in humans was obtained from HPA (https://www.proteinatlas.org/). *GPER1* RNA expression was presented as consensus datasets created by combining data from the three transcriptomics datasets (HPA, GTEx and FANTOM5) using the internal normalisation pipeline.

*GPER1* mRNA expression of tumour samples and corresponding paracancer samples were determined using TCGA (https://cancergenome.nih.gov), UCSC XENA (https://xenabrowser.net/datapages/) and GTEx (https://gtexportal.org/). Samples with ‘0’ values for gene expression were excluded. The analysis involves the direct examination of molecular distinctions in various pan-cancer datasets, enabling a comparative analysis between tumor and normal (adjacent) groups. The Wilcoxon rank sum test is employed for statistical analysis. In cases where a group consists of fewer than three observations or exhibits a standard deviation greater than zero, said groups will be excluded from the statistical analysis.

Paired samples were retained for paired sample analysis. The analysis is conducted by directly assessing molecular variations across diverse pan-cancer datasets, specifically targeting the differential analysis between tumor samples and adjacent or normal tissue groups within samples exhibiting paired relationships. Statistically, it is stipulated that each group of samples must comprise a minimum of three observations and possess a non-zero variance; failure to meet these conditions will result in the exclusion of said groups from the statistical analysis.

RNA sequencing data in Fragments Per Kilobase per million format were converted and normalised as transcripts per million reads using the Toil process and log2 transformed for further analysis [38]. The statistical analysis employs the Wilcoxon rank sum test. R software was used to perform statistical analyses in this study (version 3.6.3). The ‘ggplot2’ package was used to present *GPER1* gene expression as bar graphs in patients with pan-cancer.

### ROC curve of *GPER1* in pan-cancer

ROC curves were used to estimate the diagnostic value of *GPER1* in pan-cancer. ROC curves were calculated using the package ‘pROC’ (version 1.17.0.1) of R software and plotted by package ‘ggplot2’ (v3.3.3). The AUC, cutoff, sensitivity, specificity, positive predictive value, negative predictive value and Youden’s index (YI) were also calculated [39]. An AUC closer to 1 indicates better diagnostic value. Accordingly, an AUC of 0.5 to 0.7, 0.7 to 0.9 and 0.9 or more indicates low, good and high accuracy, respectively [40]. YI indicates the total ability of the screening method to detect real patients from non-patients, with a larger index indicating a more valid and true screening method [41, 42].

### Survival analysis of *GPER1* in pan-cancer

The ‘survival’ package was used to conduct K-M analysis. The patients with corresponding cancers in the TCGA database were divided into “high” and “low” expression level groups based on the median expression level of *GPER1*. The OS rates in the high and low *GPER1* gene expression groups were compared across 35 cancer types. The p value was determined using Cox regression analysis. The forest plots plotted the HR and 95% CI, and the p values of the survival curves were calculated and visualised using ‘survminer’ and ‘ggplot2’ (version 3.3.3) package.

### Genetic alteration analysis of *GPER1*

Genetic Alteration Analysis of *GPER1* was performed using cBioPortal (https://www.cbioportal.org/) [43]. The ‘OncoPrint’ module was used to explore genetic alterations of *GPER1*. The somatic mutation frequency and genomic information of *GPER1* mutations in pan-cancer were explored using the ‘cancer types summary and mutations’ module. The prognostic value of *GPER1* for pan-cancer was investigated using the ‘Comparison/Survival’ module.

### DEGs analysis between high and low *GPER1* expression

According to the expression value of *GPER1*, patients were divided into high and low *GPER1* expression groups, and the DEGs of the two groups were analyzed. These DEGs will be utilized for subsequent analyses involving functional enrichment and gene set enrichment.

During the detection of DEGs, it is necessary to perform differential statistical tests individually on thousands of genes within a single cancer type. This process involves multiple comparisons, which can potentially lead to false positive results, necessitating the implementation of a multiple hypothesis testing correction. We utilize the Benjamini-Hochberg (BH) method for False Discovery Rate (FDR) correction [44]. FDR is a widely employed technique for correcting multiple hypothesis testing, designed to control the proportion of erroneous rejections of null hypotheses. Compared to the conventional Bonferroni correction, the FDR method demonstrates increased applicability, particularly when facing a substantial number of hypothesis tests.

‘DESeq2’ analysis was performed in R to identify DEGs between pan-cancer patients related to *GPER1* expression using unpaired Student’s t-test, with the thresholds set at an adjusted P < 0.01 and absolute log-fold change >1. Identified genes were analysed and presented as volcano plots. The top 30 up- and downregulated genes were presented as heat maps. The correlation between *GPER1* and the top 30 up- and downregulated genes was assessed using the Spearman non-parametric correlation test. This method evaluates the presence of correlation between two sets by analyzing their rank orders. Each square represents the expression value of other molecules after undergoing z-score transformation among various samples, with the color intensity reflecting the absolute magnitude of the values. Z-score transformation is a commonly employed data conversion method in generating heatmap visualizations, utilized to mitigate expression value discrepancies across diverse molecules within a dataset. This approach involves subtracting the mean value of each molecule’s expression in individual samples from its global mean across all samples, followed by division by the standard deviation. Consequently, the data is endowed with similar scales and distributions across different molecules. This aids in diminishing the impact of extreme expression values on heatmap visualization while retaining the depiction of molecular differences among samples. Visualisation of all data was achieved using the ‘ggplot2’ package in R.

### PPI network analyses of *GPER1*

To collect and integrate potential protein interactions with *GPER1* in cancer patients whose OS was significantly associated with *GPER1* expression, the top 30 up- and downregulated DEGs for individual types of cancers were used to search the STRING database (https://string-db.org/) [45] and conducted PPI network analysis. Through this apo reach, the PPI network was strongly associated with specific cancers. A confidence score >0.7 was set as the significance threshold.

### Functional enrichment analysis of DEGs related to *GPER1* expression

The ‘clusterProfiler’ and ‘org.Hs.eg.db’ packages of R were used to conduct GO function and KEGG enrichment analyses for statistically significant DEGs. The p-value cutoff threshold for DEGs included in the GO function and KEGG enrichment analyses is set at < 0.01. The results were presented as a bubble chart via the ‘ggplot2’ (v.3.3.3). The bubble chart displays the top 4 results from each category, ranked based on the magnitude of adjusted p-values of GO/KEGG analyses’ result in ascending order, with smaller adjusted p-values corresponding to higher rankings. If the number of results is fewer than four, then all entries will be displayed. GO/KEGG joint logFC results are presented as a string and circle graph via ‘Goplot’ (version 1.0.2) and ‘ggplot2’ (version 3.3.3) package in R. The threshold employed for the categorization of data used in generating the circle graph is defined as adjusted p-values < 0.05 for the GO/KEGG joint logFC results. Results with too few enrichment entries (count < 5) cannot be displayed as a circle graph.

### Gene set enrichment analysis

GSEA was performed via the ‘clusterProfiler’ package to determine the biological pathway, GO and immunologic signature differences between the high and low *GPER1* groups. Pathways with a false discovery rate <0.25 and adjusted p value <0.05 were considered to have remarkably changed. Gene set permutation was performed 1,000 times for each analysis. The top five entries of the enrichment analysis were presented as a mountain map. DEGs not enriched to the relevant entry were not presented. GSEA results were presented using the ‘ggplot2’ package in R.

### Promoter methylation level of *GPER1* in cancers

Heatmaps of the DNA methylation of *GPER1* in cancers were obtained from the MethSurv database (https://biit.cs.ut.ee/methsurv/) [46]. Promoter methylation level of *GPER1* in cancers whose OS was significantly associated with *GPER1* expression was explored. *GPER1* methylation levels in cancers and corresponding adjacent tissues were determined from TCGA and presented via the UALCAN database [47] (http://ualcan.path.uab.edu/analysis.html). Student’s t-test was used to determine whether differences were significant. A p value of <0.05 indicated statistical significance.

### Pan-cancer immunogenomic analyses of *GPER1*

Pan-cancer immunogenomic analyses of *GPER1* was performed via the TISIDB online tool (http://cis.hku.hk/TISIDB/index.php) [48]. Correlations between expression and methylation level of *GPER1* and immune components, such as TILs, immunoinhibitors, immunostimulators, MHC molecules, chemokines and chemokine receptors in pan-cancer, were presented as heatmaps. A p value <0.05 indicated statistical significance.

### Data availability

The datasets for this study can be found in the HPA(https://www.proteinatlas.org/), TCGA Research Network (https://www.cancer.gov/tcga), GTEx (http://commonfund.nih.gov/GTEx/), UCSC Xena (http://xena.ucsc.edu/), MethSurv (https://biit.cs.ut.ee/methsurv/), UALCAN (http://ualcan.path.uab.edu/), cBioPortal (http://www.cbioportal.org/), STRING (https://string-db.org/) and TISIDB (http://cis.hku.hk/TISIDB/) databases.

## Supplementary Material

Supplementary Figures

Supplementary Tables 1 and 2

Supplementary Table 3

Supplementary Table 4

Supplementary Table 5

Supplementary Table 6

Supplementary File 1
