# Supplementary material for: Utility of G protein-coupled oestrogen receptor 1 as a biomarker for pan-cancer diagnosis, prognosis and immune infiltration: a comprehensive bioinformatics analysis
Source: Aging (Albany NY). 2023 Nov 2;15(21):12021–67. doi: 10.18632/aging.205162 (PMC10683611; doi:10.18632/aging.205162)
Supplement: Supplementary Figures [file aging-15-205162-s001.pdf]

SUPPLEMENTARY FIGURES

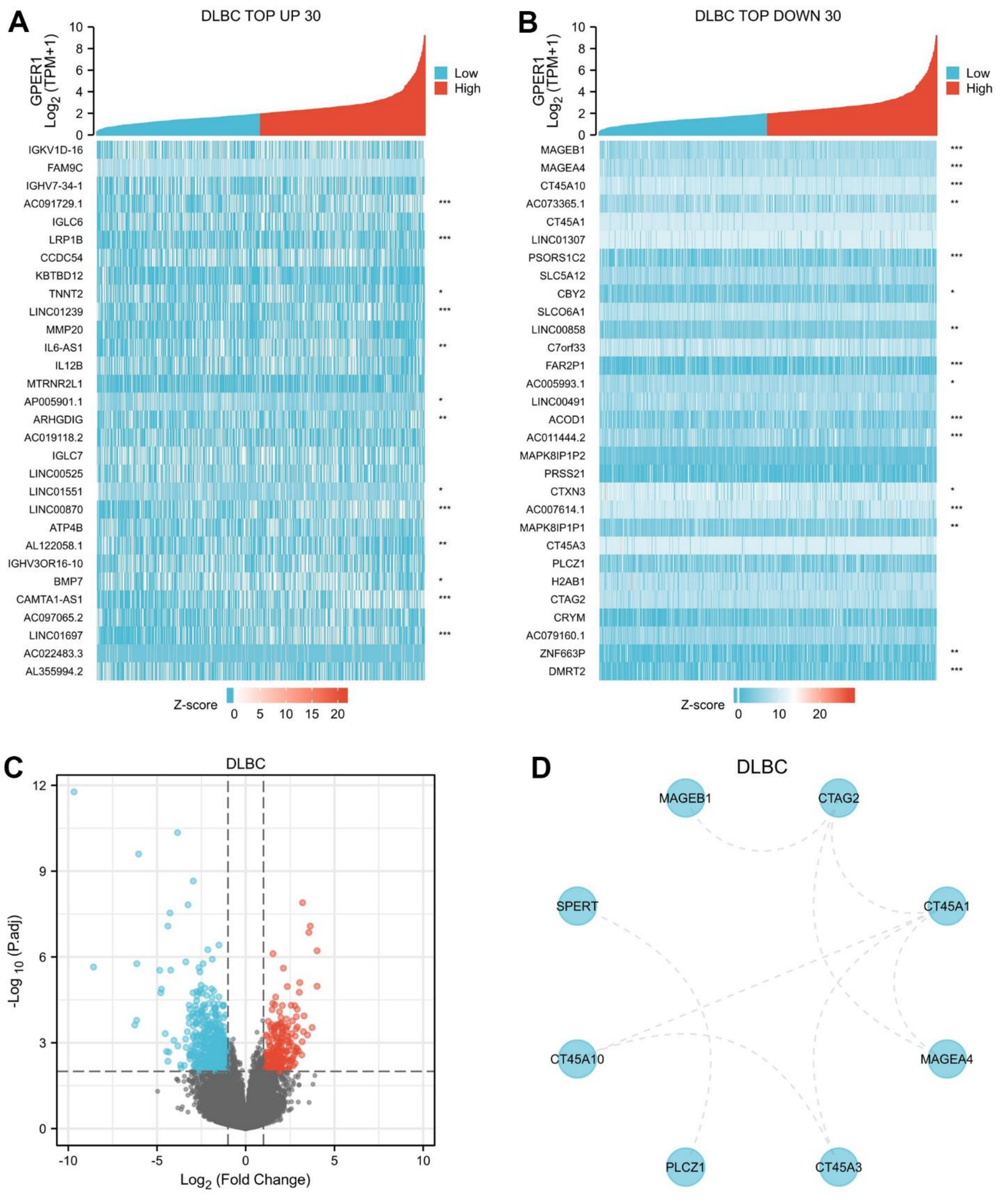

Supplementary Figure 1. DEGs of high and low *GPER1* expression in DLBC and PPI network of DEGs. (**A**) The heatmap of top 30 up-regulated DEGs, (**B**) The heatmap of top 30 down-regulated DEGs, (**C**) The volcano plots of DEGs between high and low *GPER1* expression groups, (**D**) PPI network of DEGs of high and low *GPER1* expression in DLBC.

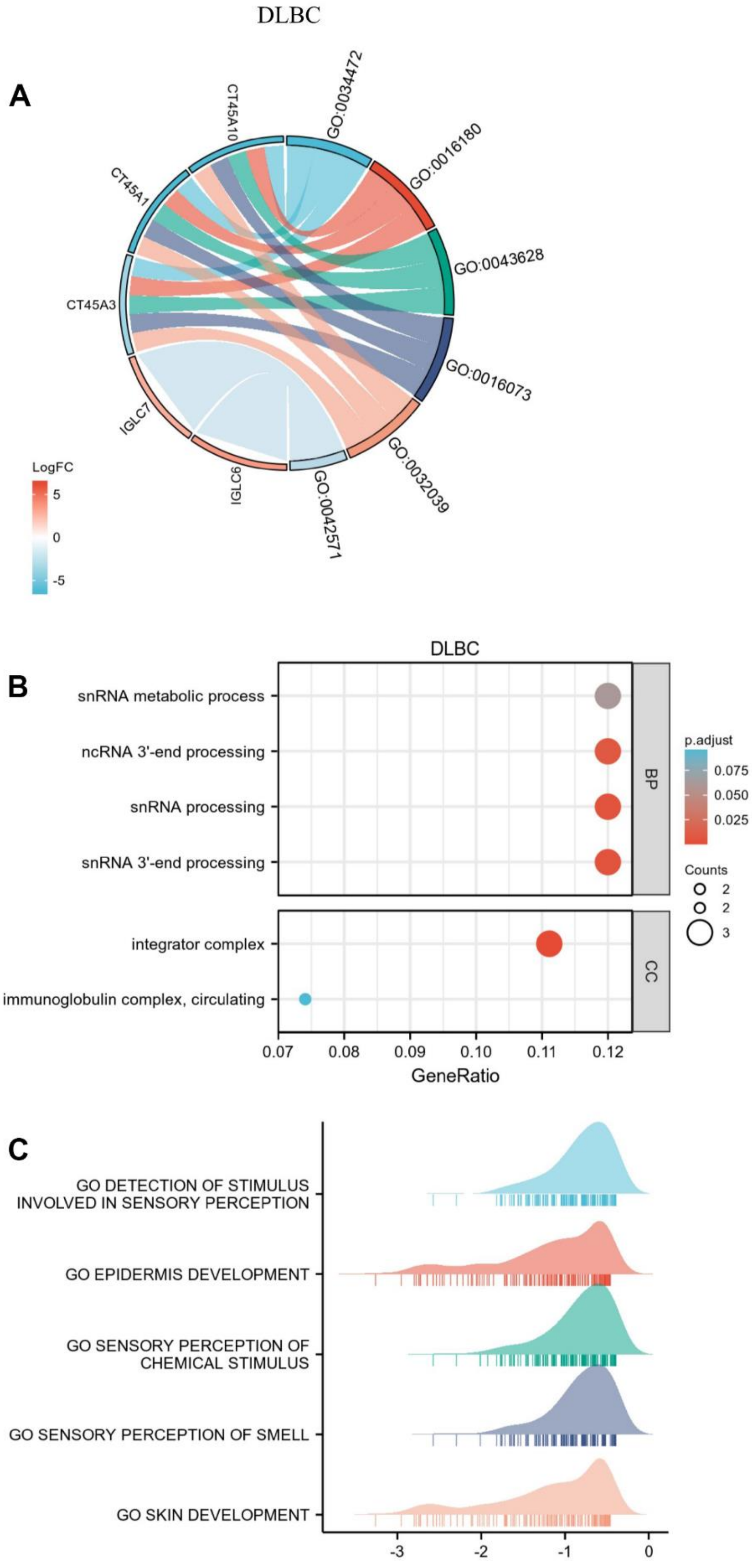

Supplementary Figure 2. Functional enrichment analysis for DEGs between High and Low expression of GPER1 expression in DLBC. (**A**) GO/KEGG pathway enrichment joint logFC for DEGs between High and -Low expression of GPER1 expression in DLBC presented as string graph. (**B**) GO/KEGG pathway enrichment presented as bubble chart, (**C**) GSEA of the signaling pathways associated with DEGs of GPER1 expression in DLBC.

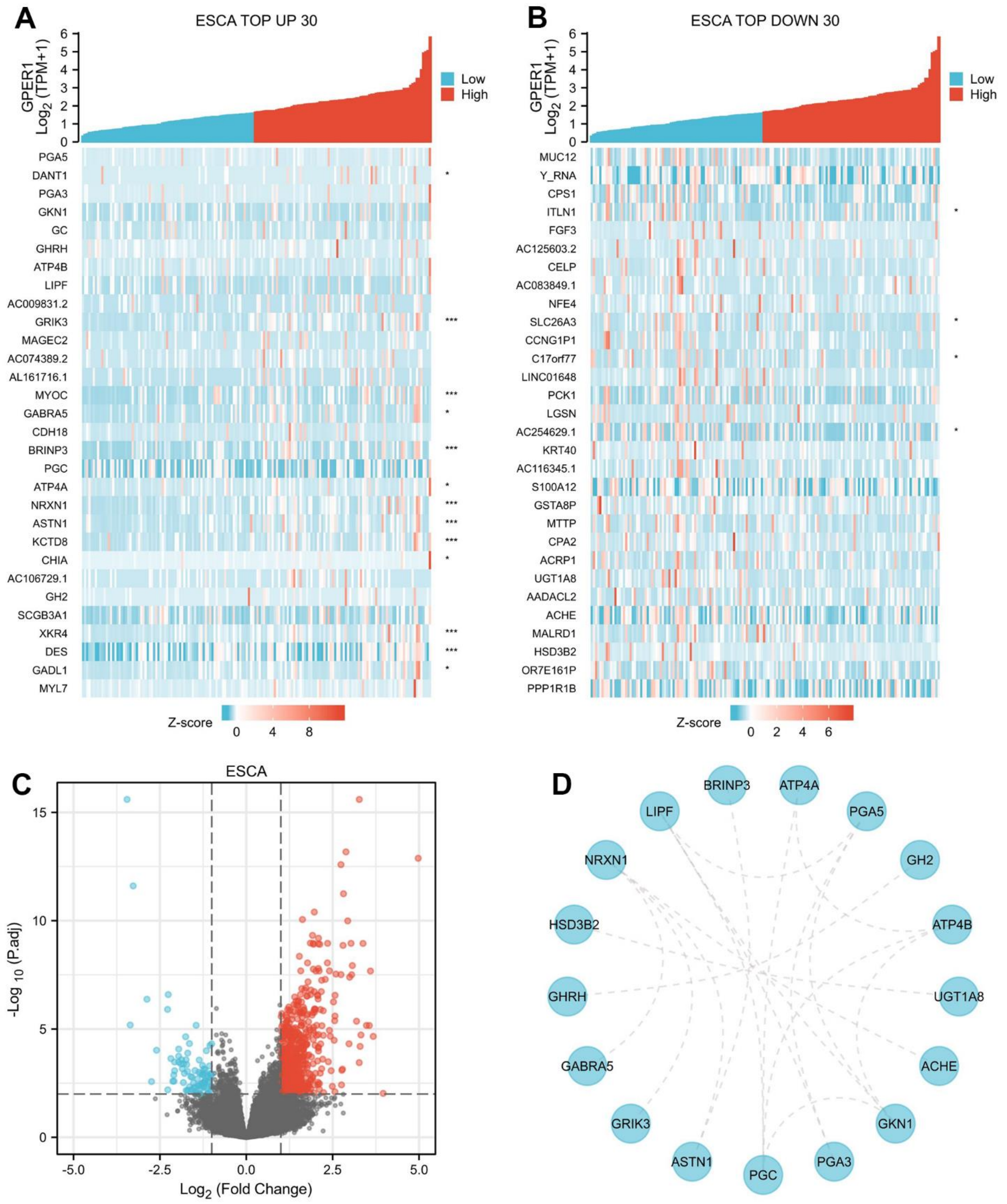

Supplementary Figure 3. DEGs of high and low *GPER1* expression in ESCA and PPI network of DEGs. (**A**) The heatmap of top 30 up-regulated DEGs, (**B**) The heatmap of top 30 down-regulated DEGs. Each square represents the expression value of other molecules after undergoing Z-score transformation across various samples (Z-score involves subtracting the mean expression value of each molecule in individual samples from its mean expression value across all samples and then dividing by the standard deviation), with color intensity indicating the absolute value of the expression level. (**C**) The volcano plots of DEGs between high and low *GPER1* expression groups, (**D**) PPI network of DEGs of high and low *GPER1* expression in ESCA.

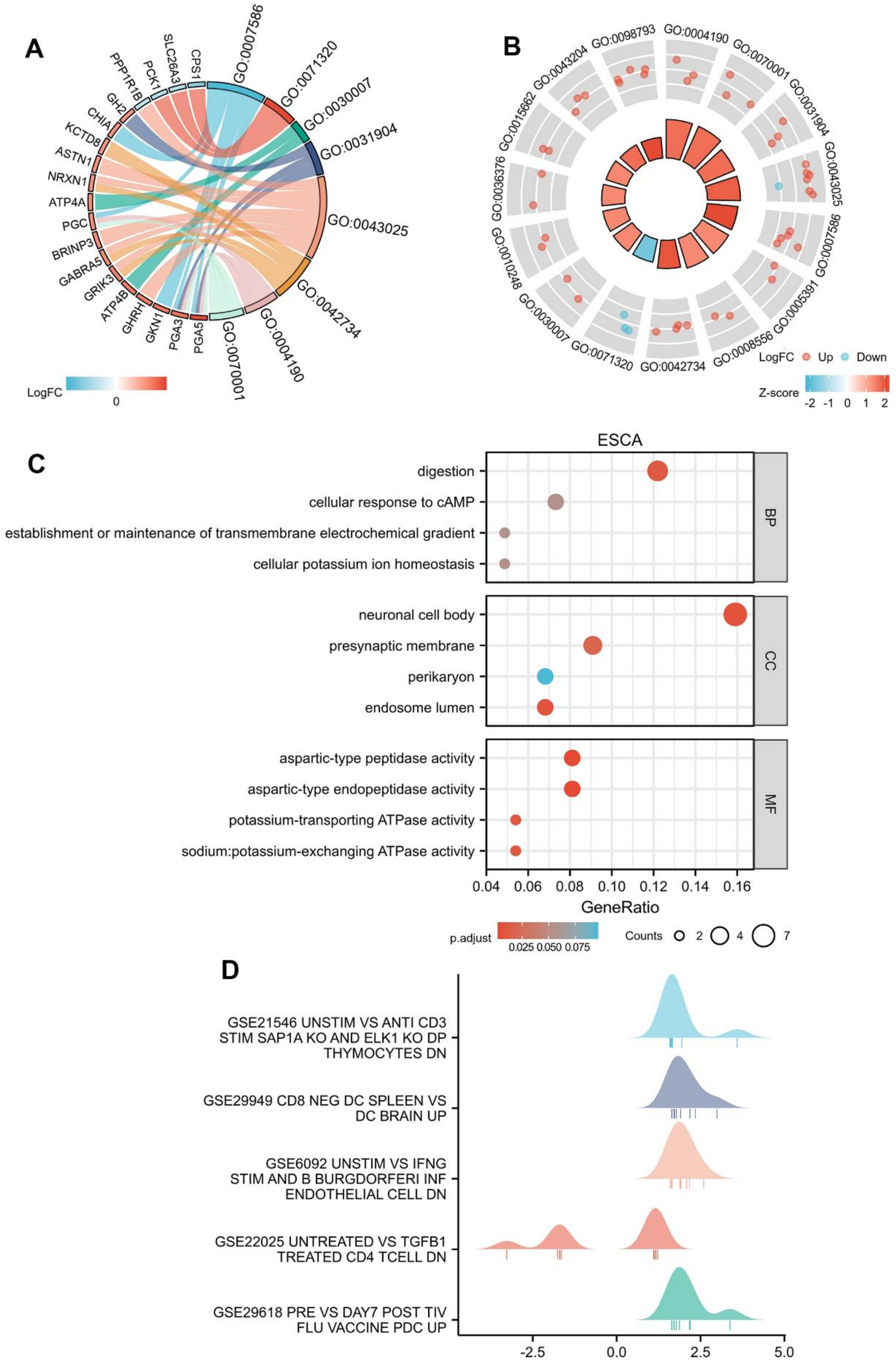

Supplementary Figure 4. Functional enrichment analysis for DEGs between High and Low expression of *GPER1* expression in ESCA. (**A**) GO/KEGG pathway enrichment joint logFC for DEGs between High and -Low expression of *GPER1* expression in ESCA presented as string graph. (**B**) GO/KEGG joint logFC results presented as circle graph. (**C**) GO/KEGG pathway enrichment presented as bubble chart, (**D**) GSEA of the signaling pathways associated with DEGs of *GPER1* expression in ESCA.

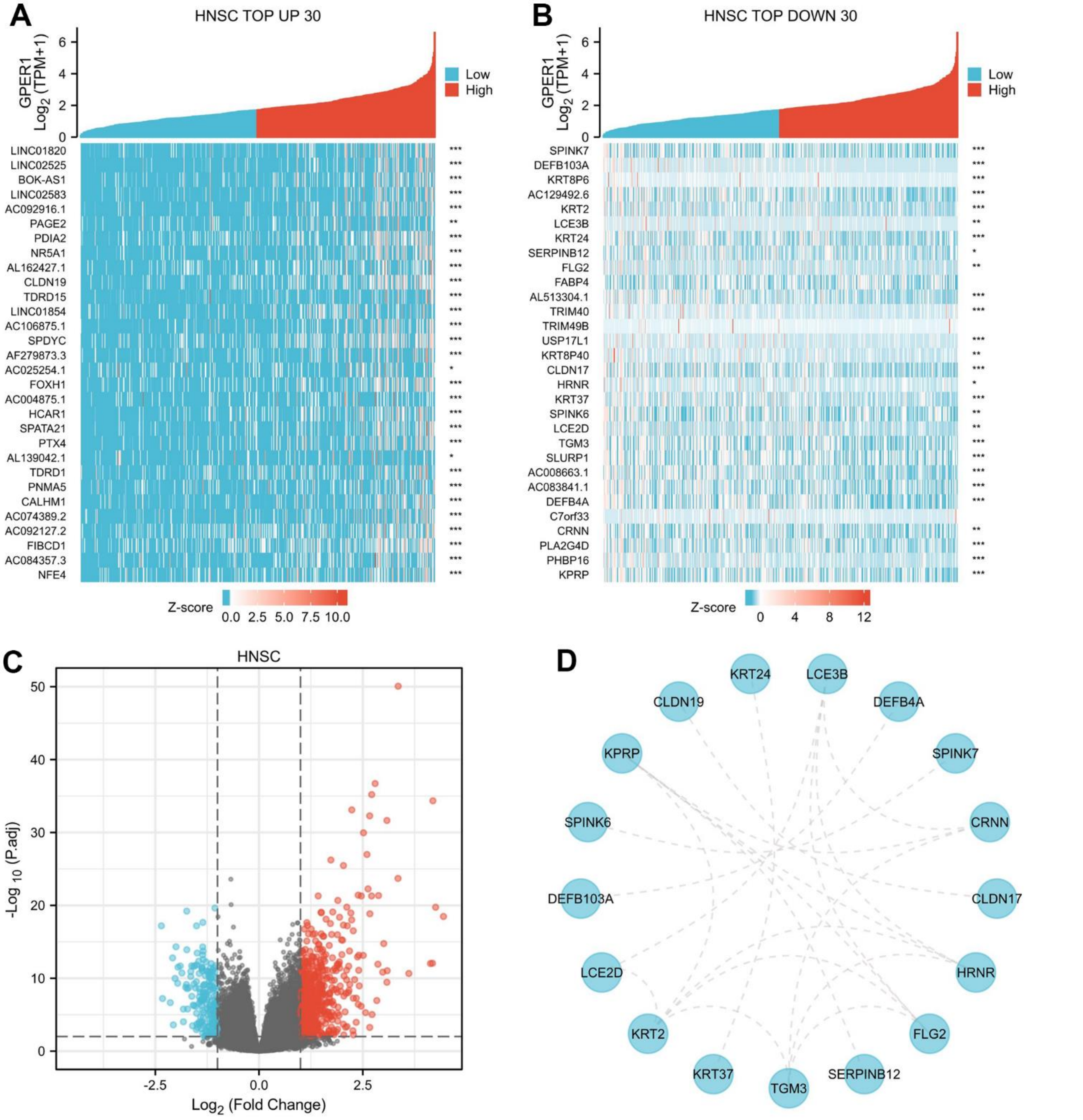

Supplementary Figure 5. DEGs of high and low *GPER1* expression in HNSC and PPI network of DEGs. (**A**) The heatmap of top 30 up-regulated DEGs, (**B**) The heatmap of top 30 down-regulated DEGs. Each square represents the expression value of other molecules after undergoing Z-score transformation across various samples (Z-score involves subtracting the mean expression value of each molecule in individual samples from its mean expression value across all samples and then dividing by the standard deviation), with color intensity indicating the absolute value of the expression level. (**C**) The volcano plots of DEGs between high and low *GPER1* expression groups, (**D**) PPI network of DEGs of high and low *GPER1* expression in HNSC.

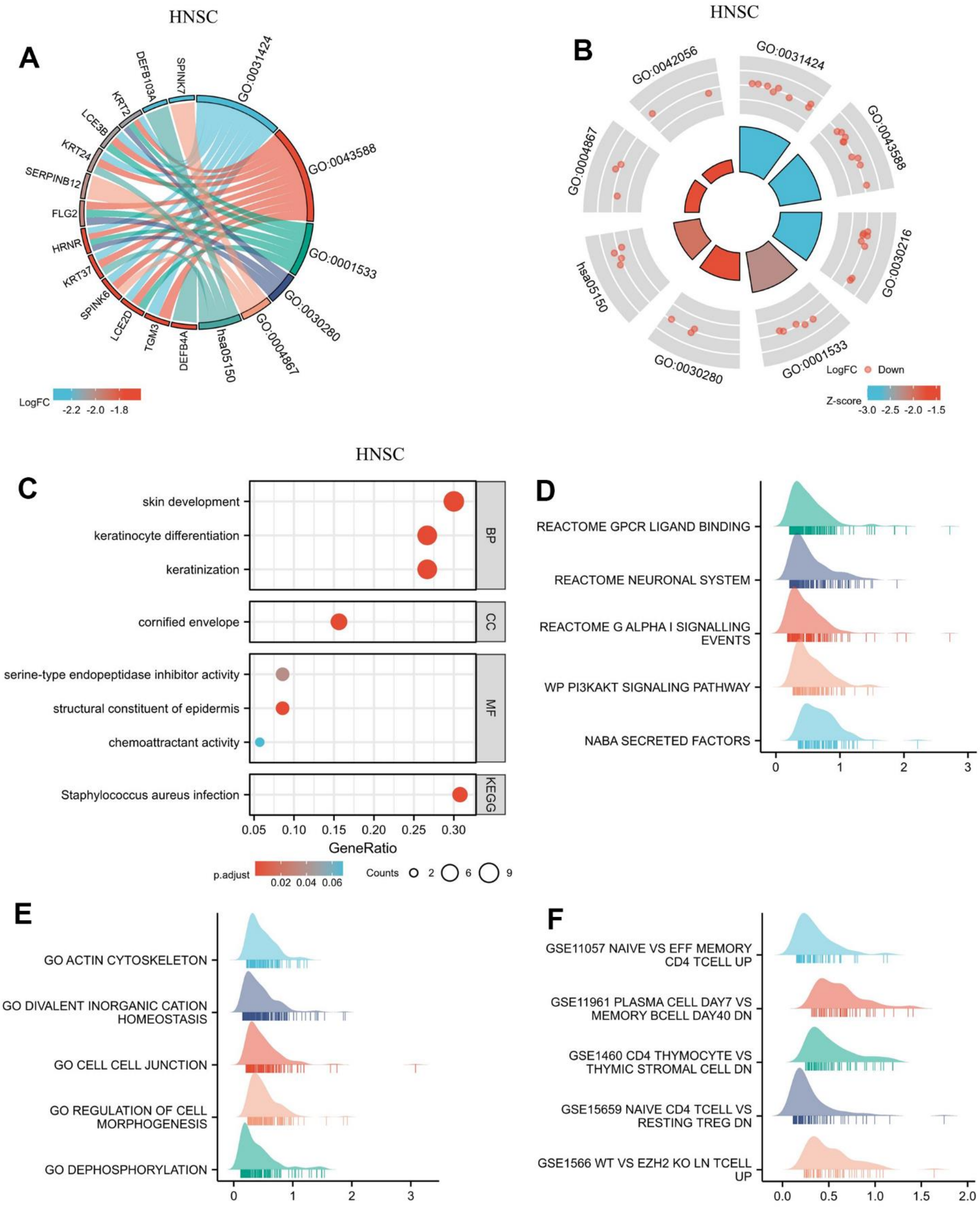

Supplementary Figure 6. Functional enrichment analysis for DEGs between High and Low expression of *GPER1* expression in HNSC. (**A**) GO/KEGG pathway enrichment joint logFC for DEGs between High and -Low expression of *GPER1* expression in HNSC presented as string graph. (**B**) GO/KEGG joint logFC results presented as circle graph. (**C**) GO/KEGG pathway enrichment presented as bubble chart, (**D**) GSEA of the biological pathways associated with DEGs of *GPER1* expression in HNSC, (**E**) GSEA of the GO associated with DEGs of *GPER1* expression in HNSC, (**F**) GSEA of the Immunologic signatures associated with DEGs of *GPER1* expression in HNSC.

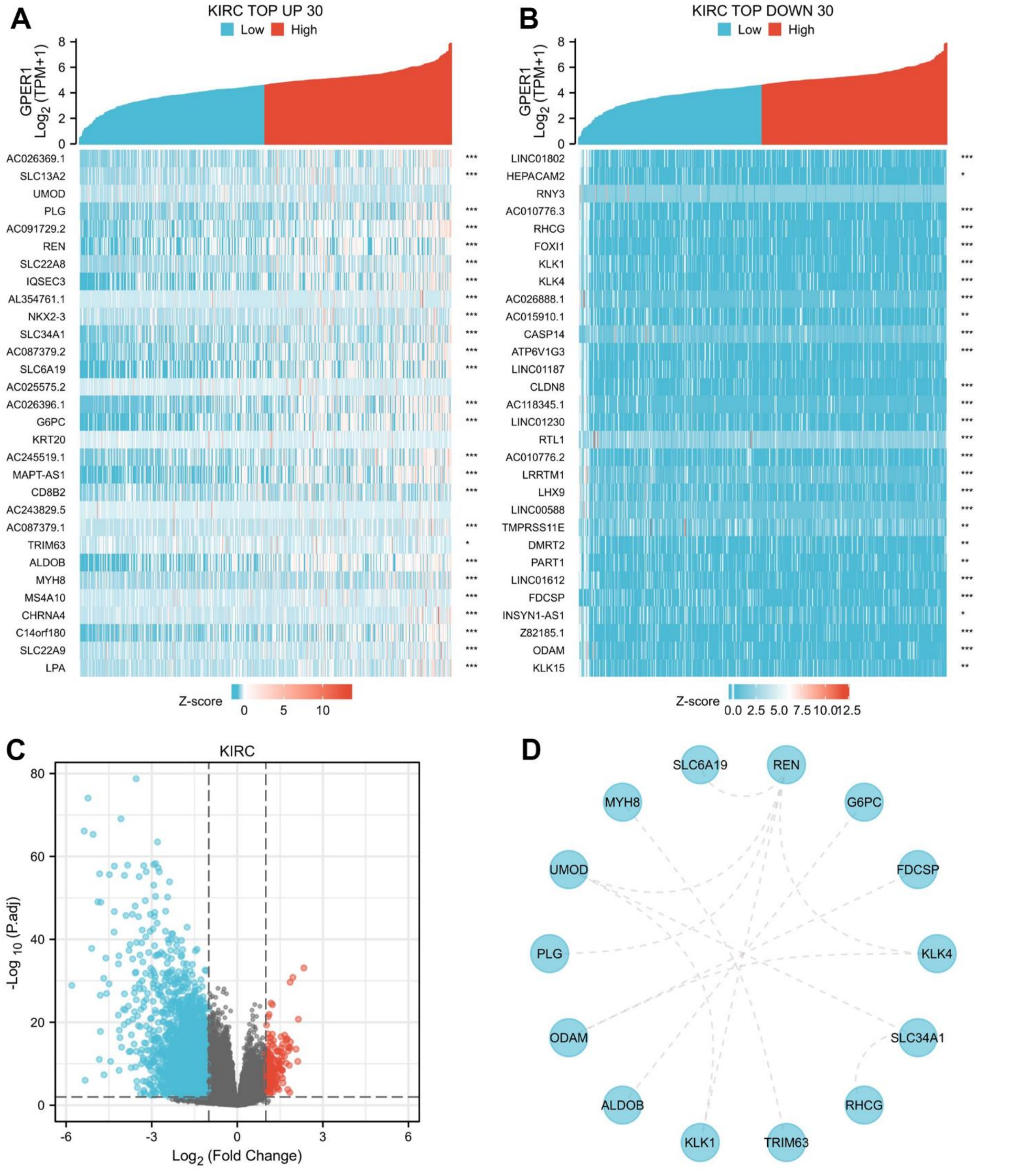

Supplementary Figure 7. DEGs of high and low *GPER1* expression in KIRC and PPI network of DEGs. (**A**) The heatmap of top 30 up-regulated DEGs, (**B**) The heatmap of top 30 down-regulated DEGs. Each square represents the expression value of other molecules after undergoing Z-score transformation across various samples (Z-score involves subtracting the mean expression value of each molecule in individual samples from its mean expression value across all samples and then dividing by the standard deviation), with color intensity indicating the absolute value of the expression level. (**C**) The volcano plots of DEGs between high and low *GPER1* expression groups, (**D**) PPI network of DEGs of high and low *GPER1* expression in KIRC.

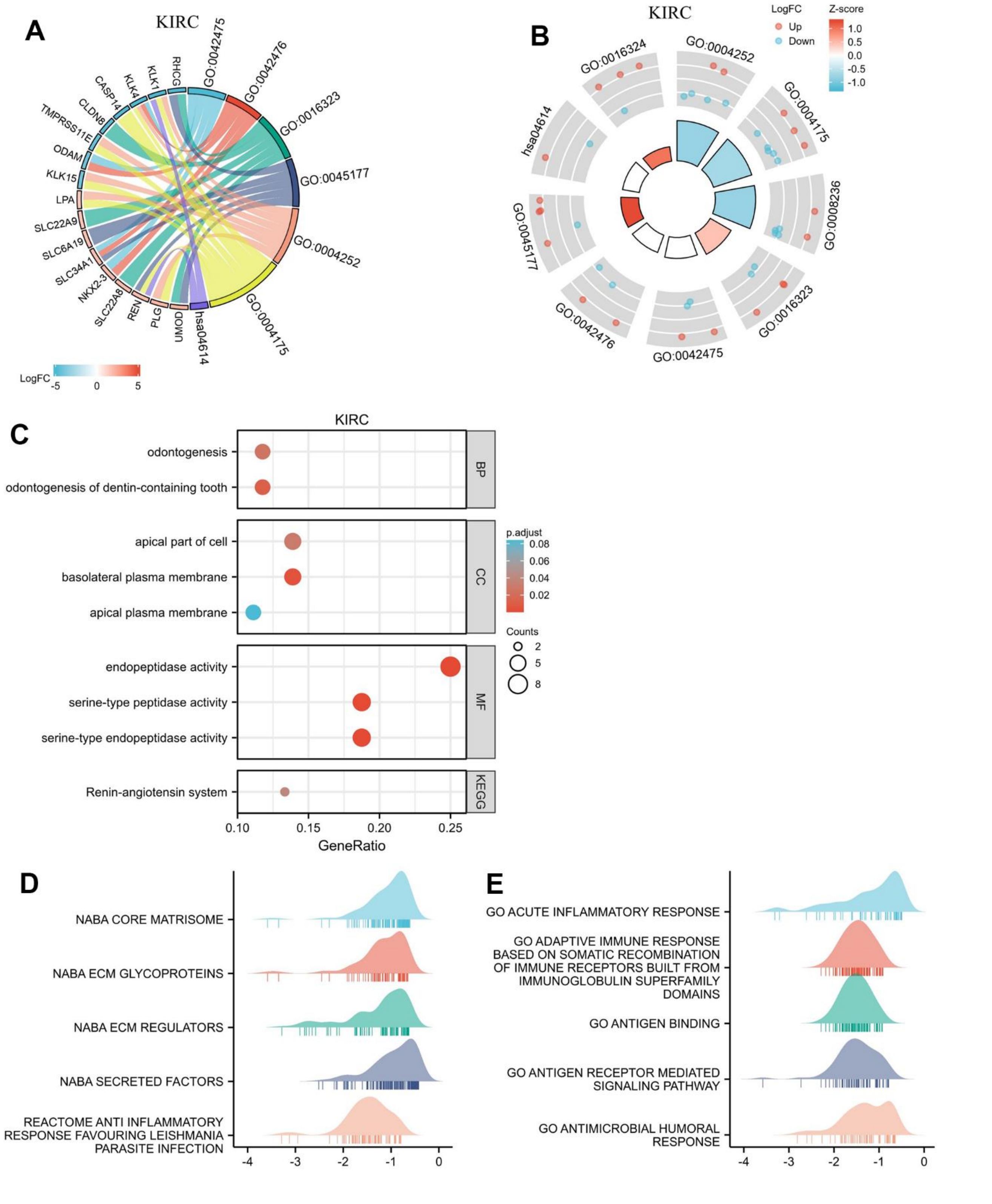

Supplementary Figure 8. Functional enrichment analysis for DEGs between High and Low expression of *GPER1* expression in KIRC. (**A**) GO/KEGG pathway enrichment joint logFC for DEGs between High and -Low expression of *GPER1* expression in KIRC presented as string graph. (**B**) GO/KEGG joint logFC results presented as circle graph. (**C**) GO/KEGG pathway enrichment presented as bubble chart, (**D**) GSEA of the biological pathways associated with DEGs of *GPER1* expression in KIRC, (**E**) GSEA of the GO associated with DEGs of *GPER1* expression in KIRC.

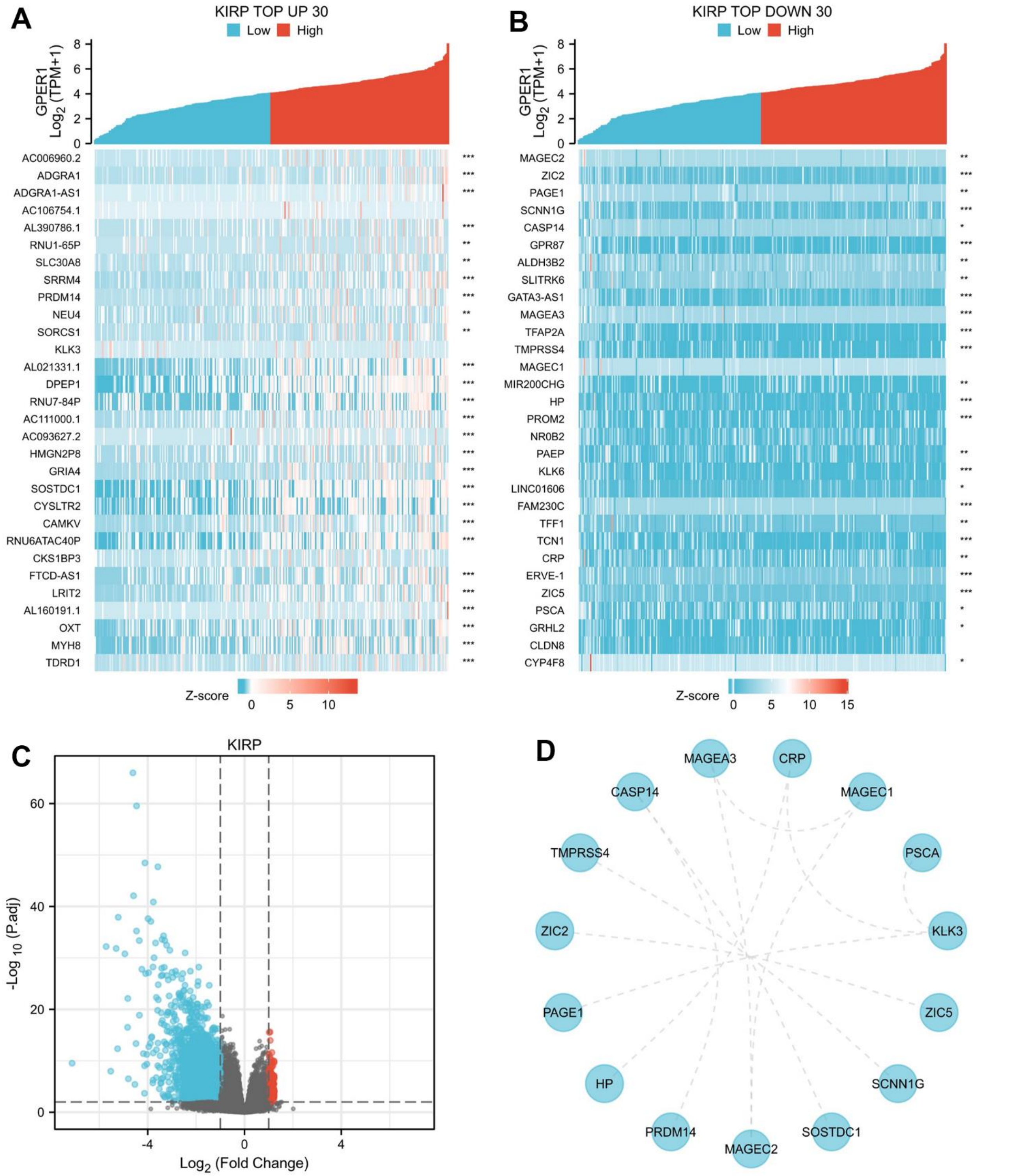

Supplementary Figure 9. DEGs of high and low *GPER1* expression in KIRP and PPI network of DEGs. (**A**) The heatmap of top 30 up-regulated DEGs, (**B**) The heatmap of top 30 down-regulated DEGs. Each square represents the expression value of other molecules after undergoing Z-score transformation across various samples (Z-score involves subtracting the mean expression value of each molecule in individual samples from its mean expression value across all samples and then dividing by the standard deviation), with color intensity indicating the absolute value of the expression level. (**C**) The volcano plots of DEGs between high and low *GPER1* expression groups, (**D**) PPI network of DEGs of high and low *GPER1* expression in KIRP.

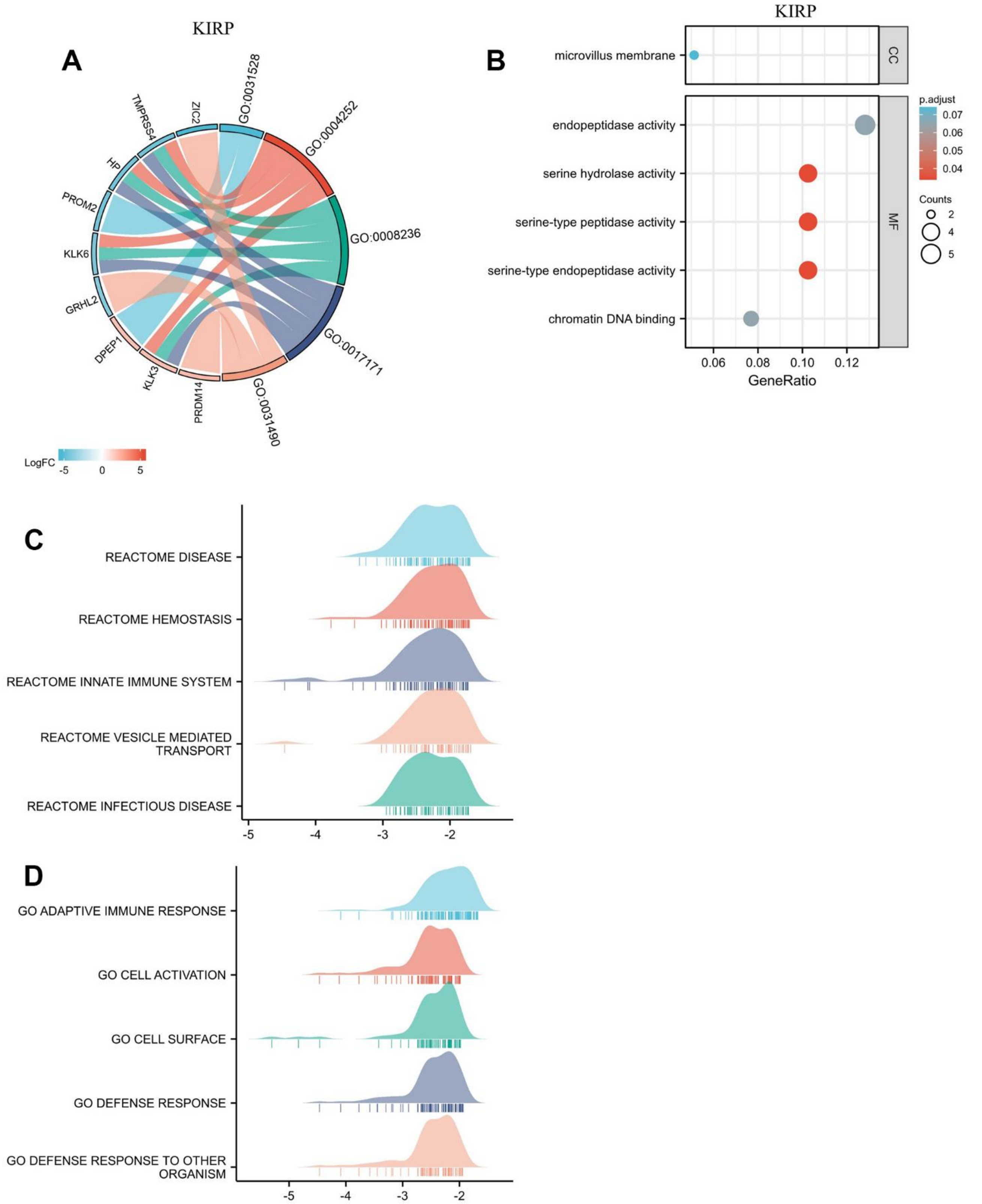

Supplementary Figure 10. Functional enrichment analysis for DEGs between High and Low expression of GPER1 expression in KIRP. (**A**) GO/KEGG pathway enrichment joint logFC for DEGs between High and -Low expression of GPER1 expression in KIRP presented as string graph. (**B**) GO/KEGG pathway enrichment presented as bubble chart, (**C**) GSEA of the biological pathways associated with DEGs of GPER1 expression in KIRP, (**D**) GSEA of the GO associated with DEGs of GPER1 expression in KIRP.

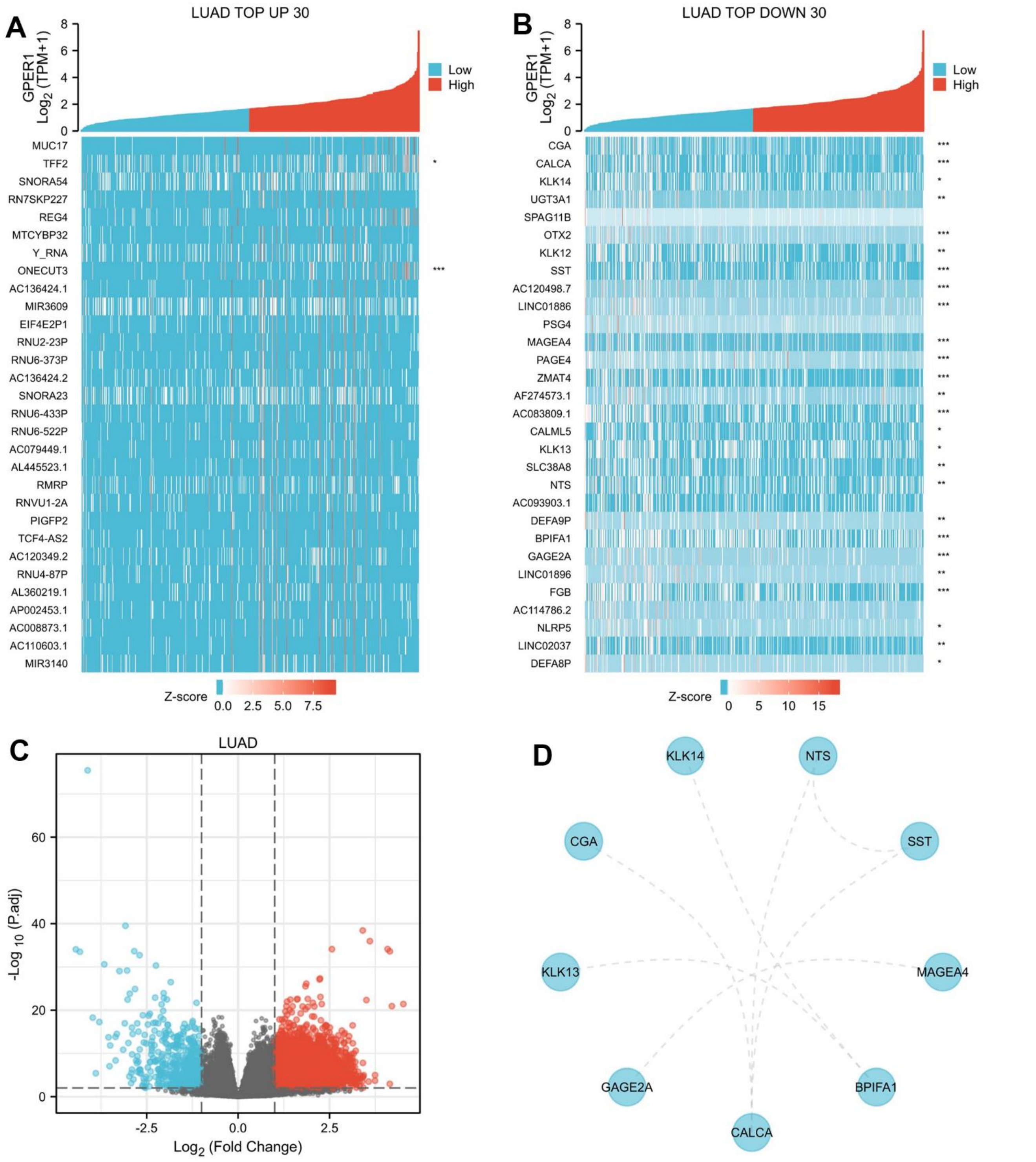

Supplementary Figure 11. DEGs of high and low *GPER1* expression in LUAD and PPI network of DEGs. (**A**) The heatmap of top 30 up-regulated DEGs, (**B**) The heatmap of top 30 down-regulated DEGs. Each square represents the expression value of other molecules after undergoing Z-score transformation across various samples (Z-score involves subtracting the mean expression value of each molecule in individual samples from its mean expression value across all samples and then dividing by the standard deviation), with color intensity indicating the absolute value of the expression level. (**C**) The volcano plots of DEGs between high and low *GPER1* expression groups, (**D**) PPI network of DEGs of high and low *GPER1* expression in LUAD.

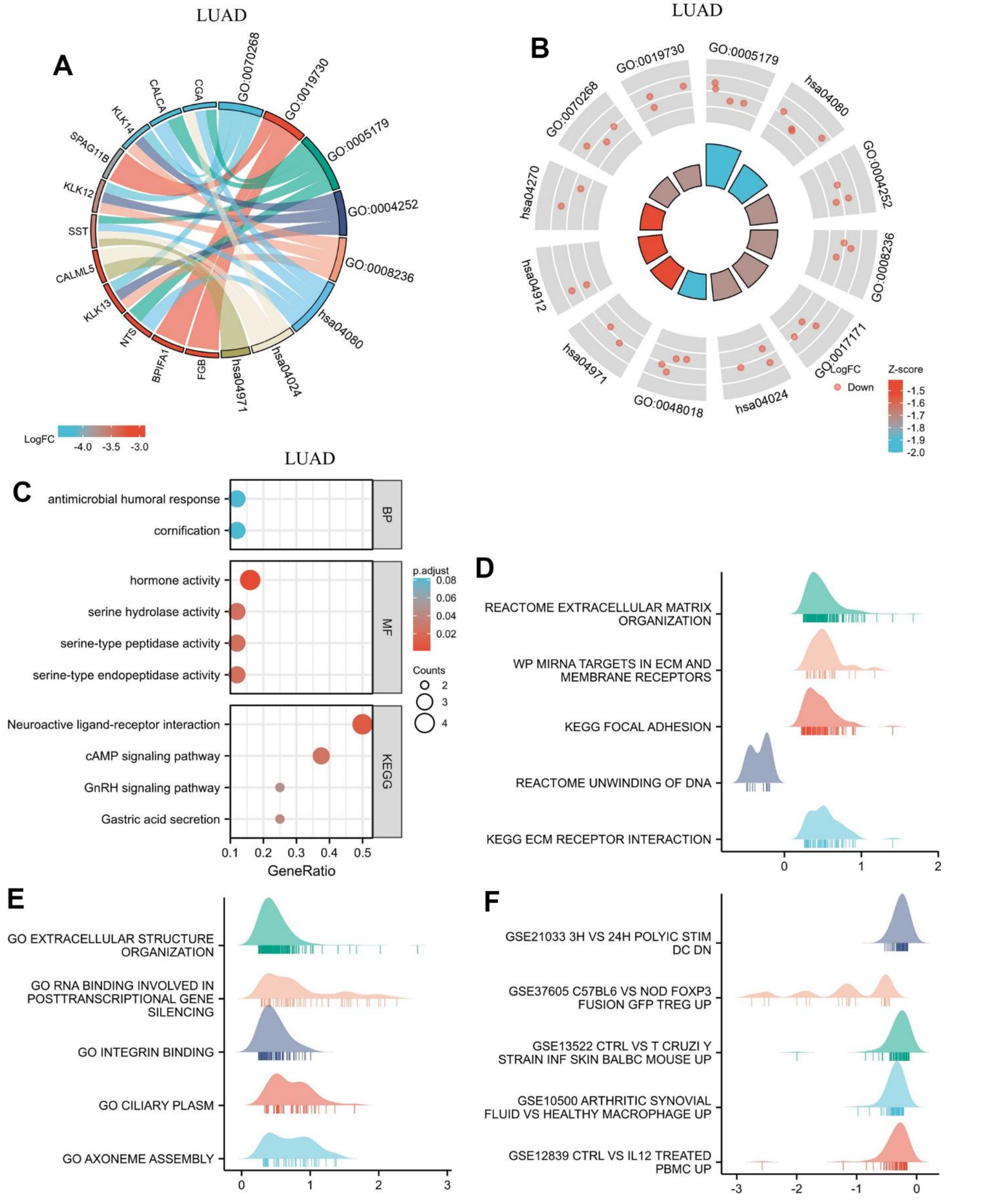

Supplementary Figure 12. Functional enrichment analysis for DEGs between High and Low expression of *GPER1* expression in LUAD. (**A**) GO/KEGG pathway enrichment joint logFC for DEGs between High and -Low expression of *GPER1* expression in LUAD presented as string graph. (**B**) GO/KEGG joint logFC results presented as circle graph. (**C**) GO/KEGG pathway enrichment presented as bubble chart, (**D**) GSEA of the biological pathways associated with DEGs of *GPER1* expression in LUAD, (**E**) GSEA of the GO associated with DEGs of *GPER1* expression in LUAD, (**F**) GSEA of the Immunologic signatures associated with DEGs of *GPER1* expression in LUAD.

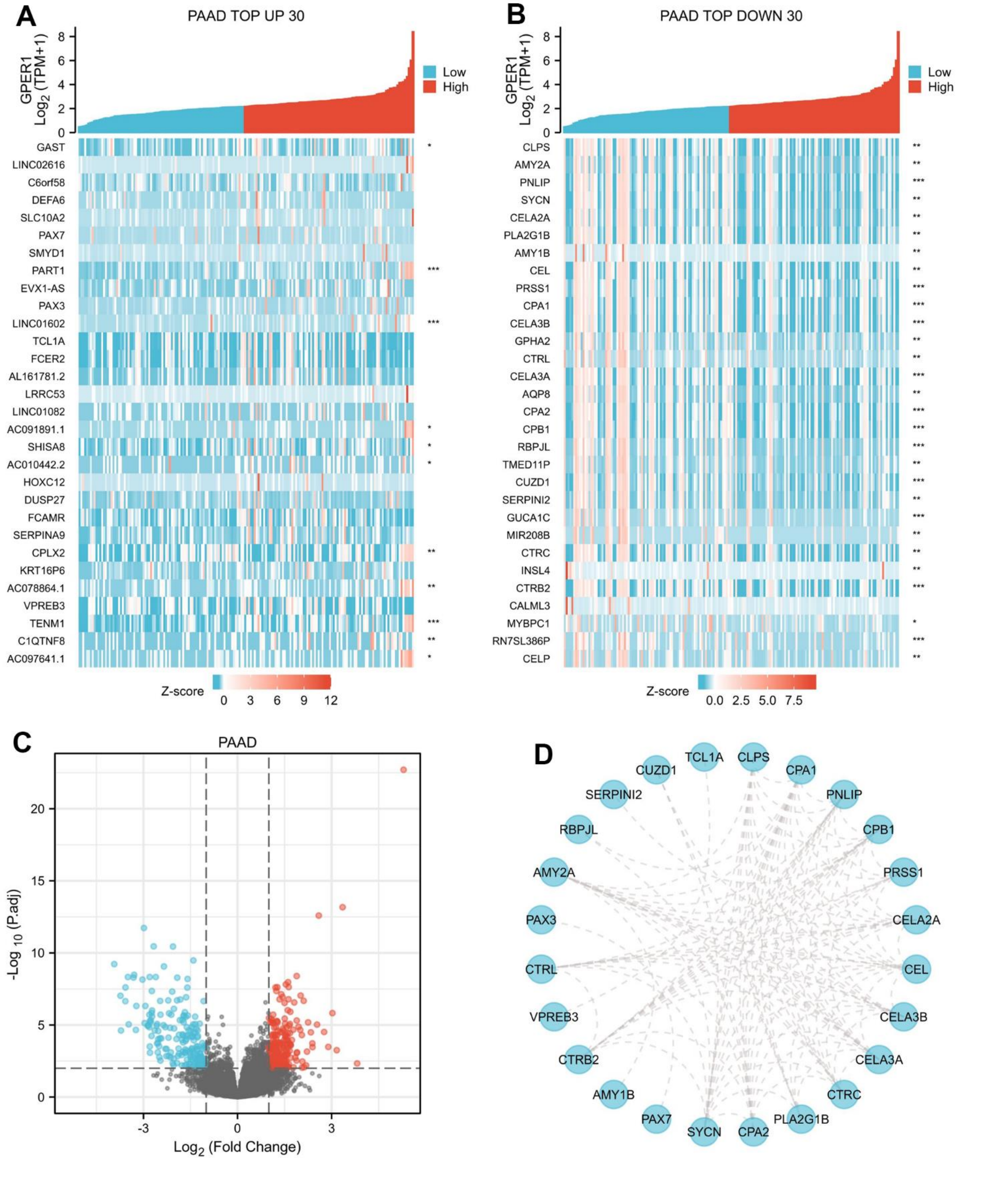

Supplementary Figure 13. DEGs of high and low *GPER1* expression in PAAD and PPI network of DEGs. (**A**) The heatmap of top 30 up-regulated DEGs, (**B**) The heatmap of top 30 down-regulated DEGs. Each square represents the expression value of other molecules after undergoing Z-score transformation across various samples (Z-score involves subtracting the mean expression value of each molecule in individual samples from its mean expression value across all samples and then dividing by the standard deviation), with color intensity indicating the absolute value of the expression level. (**C**) The volcano plots of DEGs between high and low *GPER1* expression groups, (**D**) PPI network of DEGs of high and low *GPER1* expression in PAAD.

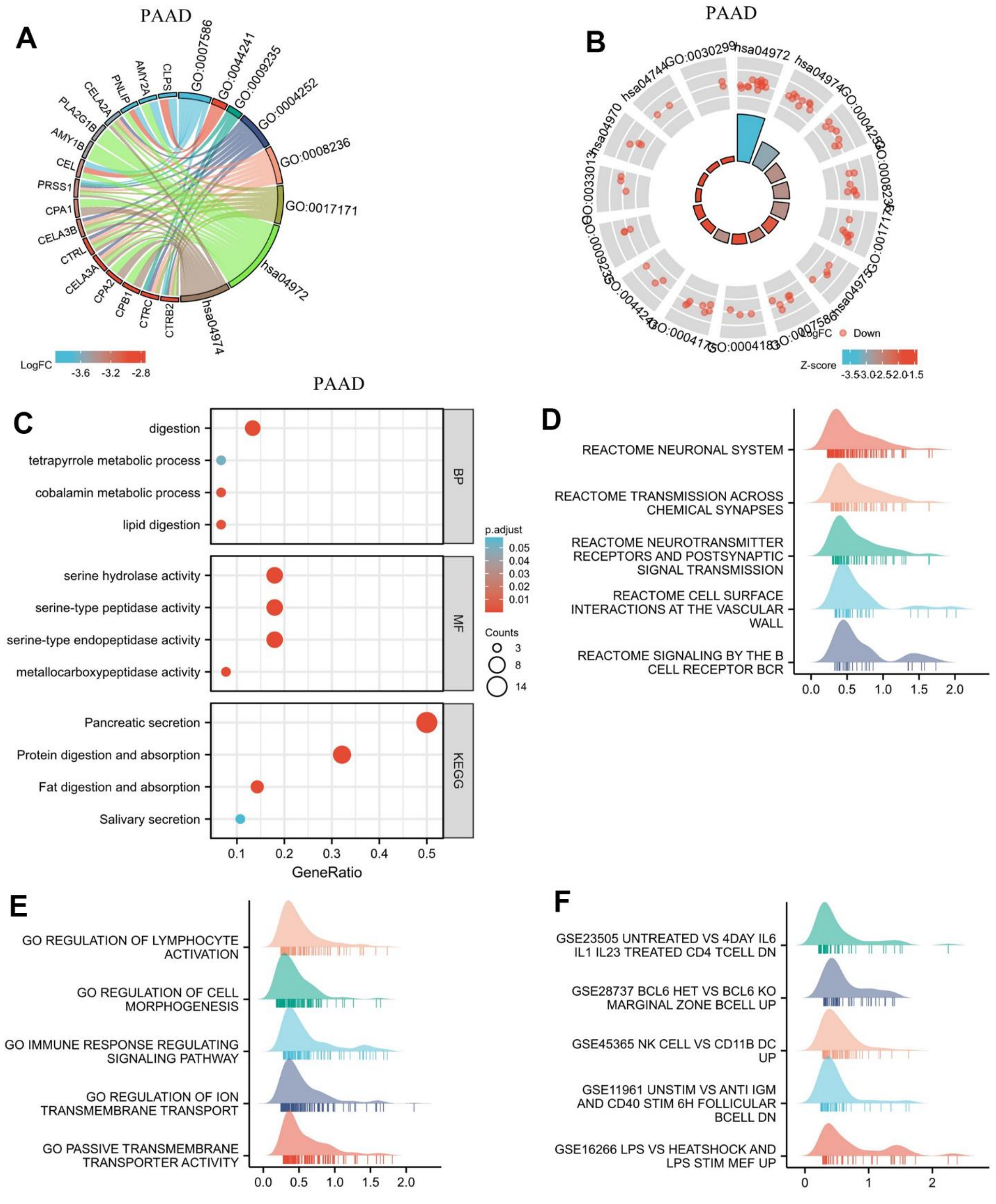

Supplementary Figure 14. Functional enrichment analysis for DEGs between High and Low expression of *GPER1* expression in PAAD. (**A**) GO/KEGG pathway enrichment joint logFC for DEGs between High and -Low expression of *GPER1* expression in PAAD presented as string graph. (**B**) GO/KEGG joint logFC results presented as circle graph. (**C**) GO/KEGG pathway enrichment presented as bubble chart, (**D**) GSEA of the biological pathways associated with DEGs of *GPER1* expression in PAAD, (**E**) GSEA of the GO associated with DEGs of *GPER1* expression in PAAD, (**F**) GSEA of the Immunologic signatures associated with DEGs of *GPER1* expression in PAAD.

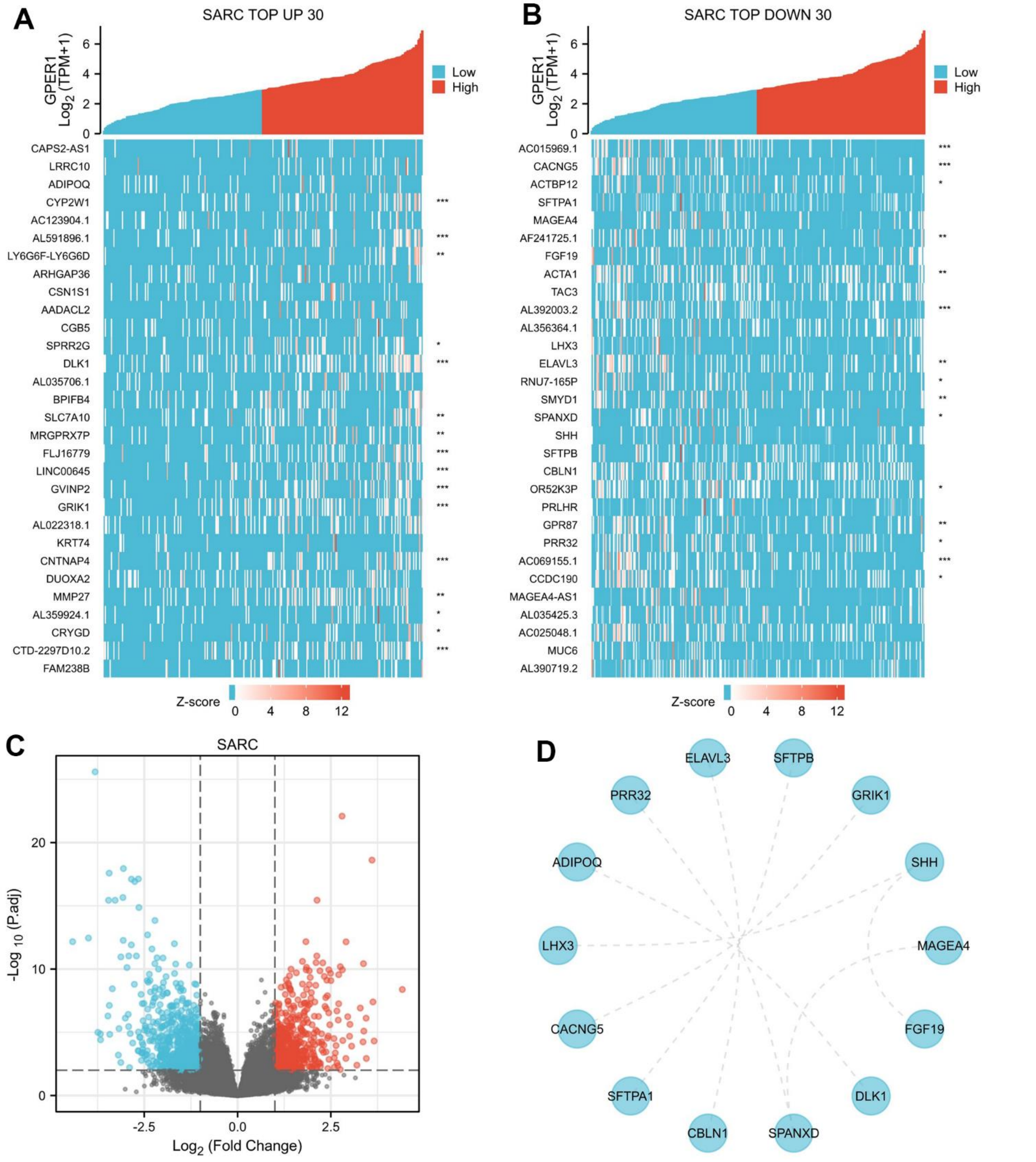

Supplementary Figure 15. DEGs of high and low *GPER1* expression in SARC and PPI network of DEGs. (**A**) The heatmap of top 30 up-regulated DEGs, (**B**) The heatmap of top 30 down-regulated DEGs. Each square represents the expression value of other molecules after undergoing Z-score transformation across various samples (Z-score involves subtracting the mean expression value of each molecule in individual samples from its mean expression value across all samples and then dividing by the standard deviation), with color intensity indicating the absolute value of the expression level. (**C**) The volcano plots of DEGs between high and low *GPER1* expression groups, (**D**) PPI network of DEGs of high and low *GPER1* expression in SARC.

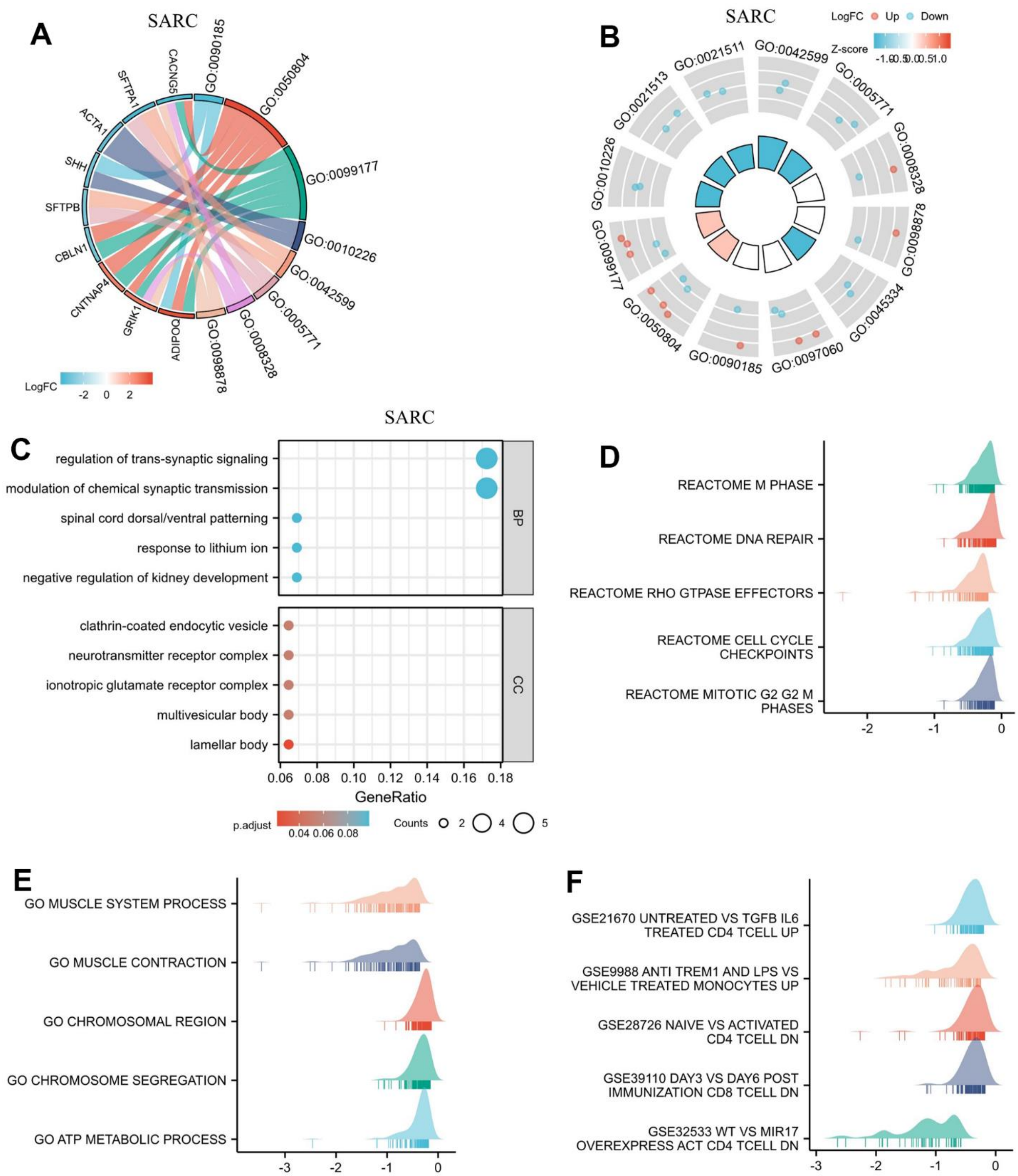

Supplementary Figure 16. Functional enrichment analysis for DEGs between High and Low expression of *GPER1* expression in SARC. (**A**) GO/KEGG pathway enrichment joint logFC for DEGs between High and -Low expression of *GPER1* expression in SARC presented as string graph. (**B**) GO/KEGG joint logFC results presented as circle graph. (**C**) GO/KEGG pathway enrichment presented as bubble chart, (**D**) GSEA of the biological pathways associated with DEGs of *GPER1* expression in SARC, (**E**) GSEA of the GO associated with DEGs of *GPER1* expression in SARC, (**F**) GSEA of the Immunologic signatures associated with DEGs of *GPER1* expression in SARC.

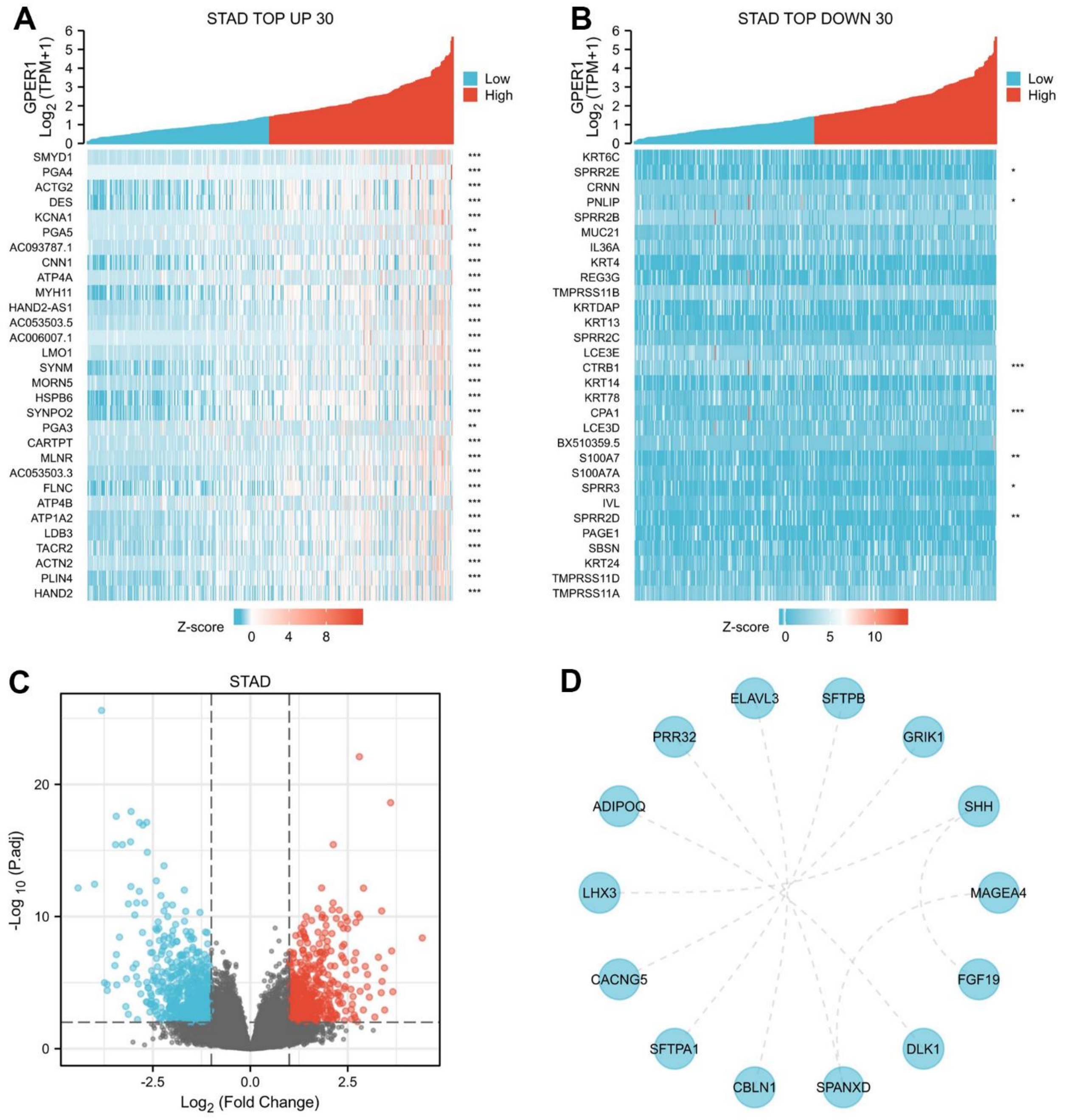

Supplementary Figure 17. DEGs of high and low *GPER1* expression in STAD and PPI network of DEGs. (**A**) The heatmap of top 30 up-regulated DEGs, (**B**) The heatmap of top 30 down-regulated DEGs. Each square represents the expression value of other molecules after undergoing Z-score transformation across various samples (Z-score involves subtracting the mean expression value of each molecule in individual samples from its mean expression value across all samples and then dividing by the standard deviation), with color intensity indicating the absolute value of the expression level. (**C**) The volcano plots of DEGs between high and low *GPER1* expression groups, (**D**) PPI network of DEGs of high and low *GPER1* expression in STAD.

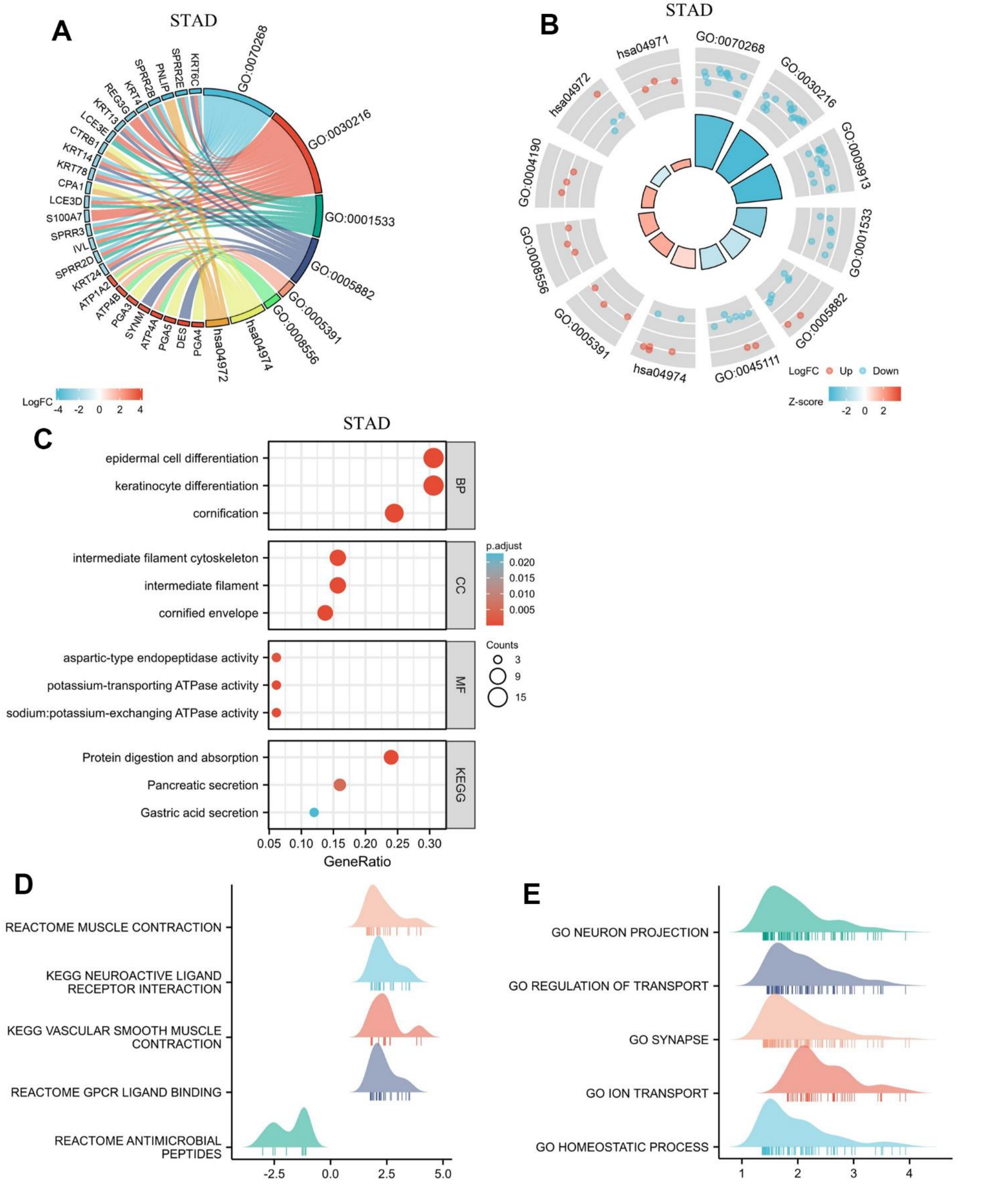

Supplementary Figure 18. Functional enrichment analysis for DEGs between High and Low expression of *GPER1* expression in STAD. (**A**) GO/KEGG pathway enrichment joint logFC for DEGs between High and -Low expression of *GPER1* expression in STAD presented as string graph. (**B**) GO/KEGG joint logFC results presented as circle graph. (**C**) GO/KEGG pathway enrichment presented as bubble chart, (**D**) GSEA of the biological pathways associated with DEGs of *GPER1* expression in STAD, (**E**) GSEA of the GO associated with DEGs of *GPER1* expression in STAD.

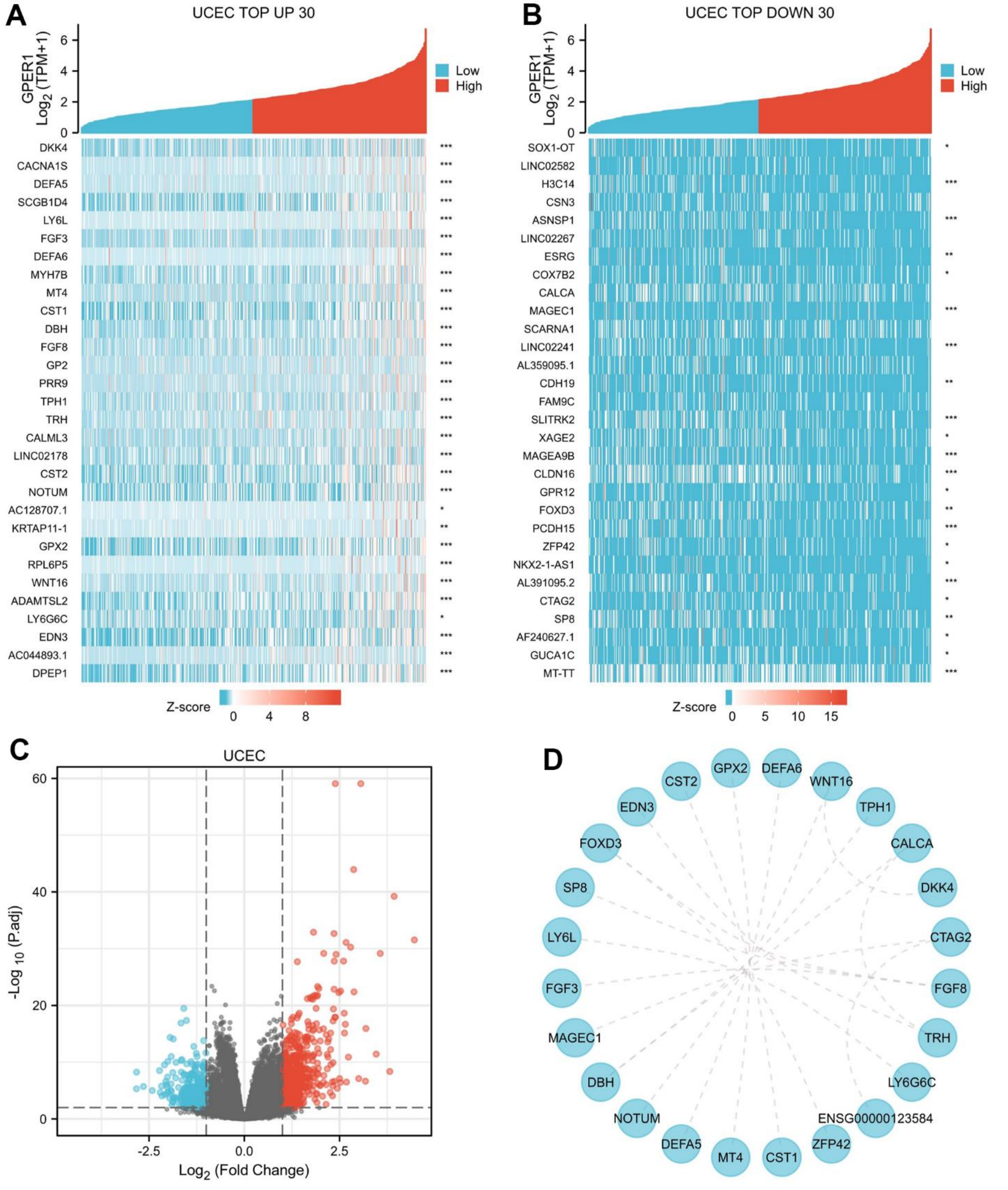

Supplementary Figure 19. DEGs of high and low *GPER1* expression in UCEC and PPI network of DEGs. (**A**) The heatmap of top 30 up-regulated DEGs, (**B**) The heatmap of top 30 down-regulated DEGs. Each square represents the expression value of other molecules after undergoing Z-score transformation across various samples (Z-score involves subtracting the mean expression value of each molecule in individual samples from its mean expression value across all samples and then dividing by the standard deviation), with color intensity indicating the absolute value of the expression level. (**C**) The volcano plots of DEGs between high and low *GPER1* expression groups, (**D**) PPI network of DEGs of high and low *GPER1* expression in UCEC.

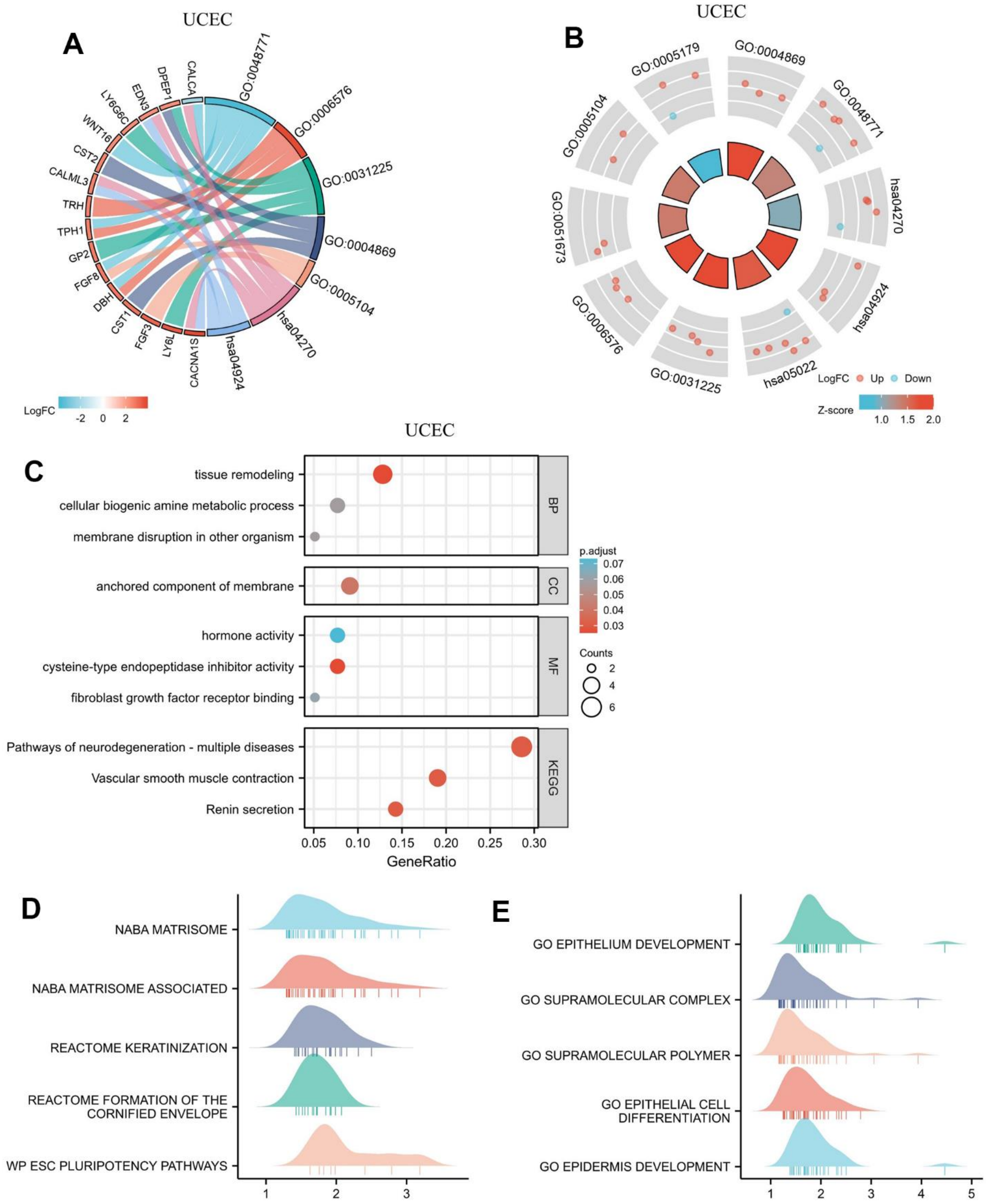

Supplementary Figure 20. Functional enrichment analysis for DEGs between High and Low expression of *GPER1* expression in UCEC. (**A**) GO/KEGG pathway enrichment joint logFC for DEGs between High and -Low expression of *GPER1* expression in UCEC presented as string graph. (**B**) GO/KEGG joint logFC results presented as circle graph. (**C**) GO/KEGG pathway enrichment presented as bubble chart, (**D**) GSEA of the biological pathways associated with DEGs of *GPER1* expression in UCEC, (**E**) GSEA of the GO associated with DEGs of *GPER1* expression in UCEC.

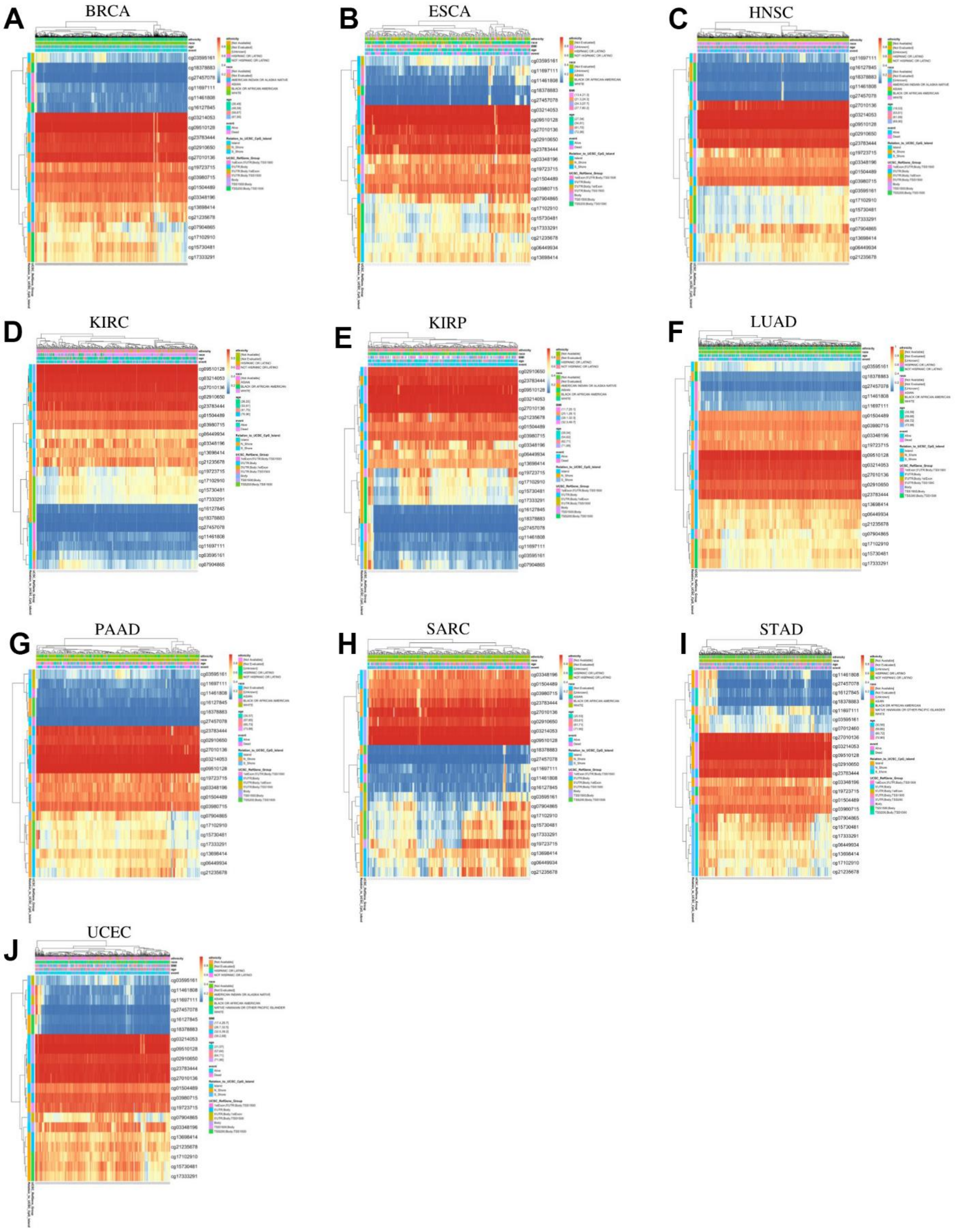

Supplementary Figure 21. The heatmaps of DNA methylation of *GPER1* in (**A**) BLCA. (**B**) ESCA, (**C**) HNSC, (**D**) KIRC, (**E**) KIRP, (**F**) LUAD, (**G**) PAAD, (**H**) SARC, (**I**) STAD, (**J**) UCEC.
