# Supplementary material for: Utility of G protein-coupled oestrogen receptor 1 as a biomarker for pan-cancer diagnosis, prognosis and immune infiltration: a comprehensive bioinformatics analysis
Source: Aging (Albany NY). 2023 Nov 2;15(21):12021–67. doi: 10.18632/aging.205162 (PMC10683611; doi:10.18632/aging.205162)
Supplement: Supplementary Tables 1 and 2 [file aging-15-205162-s002.pdf]

SUPPLEMENTARY TABLES

Supplementary Table 1. Details of GPER1 ROC in pan-cancer.

Tumor type	Tumor(n)	Normal(n)	AUC(CI)	Cut-off	Sensitivity	Specificity	Positive predictive value	Negative predictive value	YI
ACC	128	77	0.687(0.598-0.776)	2.033	0.532	0.922	0.804	0.766	0.454
BLCA	407	28	0.896(0.835-0.958)	1.272	0.865	0.786	0.983	0.286	0.651
BRCA	1099	292	0.849(0.827-0.871)	1.763	0.768	0.832	0.945	0.488	0.6
CESC	306	13	0.843(0.770-0.917)	1.128	0.596	1.000	1.000	0.095	0.595
**CHOL**	**36**	**9**	**0.951(0.886-1.000)**	**3.181**	**0.889**	**0.917**	**0.727**	**0.971**	**0.806**
**COAD**	**290**	**349**	**0.964(0.951-0.977)**	**1.707**	**0.900**	**0.928**	**0.913**	**0.918**	**0.828**
DLBC	47	444	0.492(0.422-0.561)	1.373	0.596	0.523	0.117	0.924	0.118
ESCA	182	666	0.781(0.745-0.818)	1.578	0.742	0.686	0.392	0.907	0.428
GBM	689	1157	0.551(0.524-0.577)	2.410	.0.787	0.310	0.404	0.709	0.097
HNSC	44	502	0.784(0.725-0.843)	1.037	0.745	0.791	0.228	0.968	0.495
**KICH**	**66**	**53**	**0.902(0.835-0.970)**	**1.491**	**0.909**	**0.868**	**0.896**	**0.885**	**0.777**
KIRC	531	100	0.690(0.635-0.745)	3.079	0.678	0.650	0.911	0.275	0.328
KIRP	289	60	0.568(0.494)0.641	3.299	0.478	0.750	0.902	0.230	0.228
**LAML**	**173**	**70**	**0.959(0.936-0.983)**	**0.373**	**0.913**	**0.914**	**0.963**	**0.81**	**0.828**
LGG	523	1152	0.548(0.519-0.577)	2.058	0.946	0.161	0.339	0.869	0.107
LIHC	371	160	0.548(0.497-0.598)	2.683	0.447	0.706	0.779	0.355	0.154
**LUAD**	**535**	**59**	**0.925(0.897-0.952)**	**2.704**	**0.898**	**0.865**	**0.424**	**0.987**	**0.764**
**LUSC**	**498**	**338**	**0.919(0.899-0.939)**	**1.590**	**0.884**	**0.822**	**0.880**	**0.827**	**0.706**
OSCC	329	32	0.753 (0.678-0.828)	1.627	0.906	0.520	0.155	0.983	0.426
OV	427	88	0.787(0.741-0.833)	1.046	0.621	0.830	0.946	0.311	0.450
PAAD	179	171	0.854(0.813-0.895)	1.014	0.777	0.836	0.832	0.781	0.613
PRAD	496	152	0.787(0.749-0.824)	2.272	0.659	0.822	0.924	0.425	0.482
**READ**	**93**	**318**	**0.965(0.947-0.983)**	**1.676**	**0.882**	**0.947**	**0.828**	**0.965**	**0.828**
SKCM	469	813	0.596(0.561-0.632)	0.884	0.337	0.899	0.658	0.702	0.236
**STAD**	**414**	**210**	**0.940(0.919-0.960)**	**2.555**	**0.903**	**0.848**	**0.921**	**0.817**	**0.751**
TGCT	154	165	0.831(0.779-0.883)	1.094	0.747	0.952	0.935	0.801	0.698
THCA	512	338	0.723(0.689-0.757)	2.880	0.611	0.766	0.798	0.566	0.378
THYM	119	446	0.469(0.420-0.517)	2.252	0.824	0.377	0.261	0.889	0.200
**UCEC**	**181**	**101**	**0.911(0.877-0.945)**	**1.805**	**0.845**	**0.901**	**0.939**	**0.765**	**0.746**
UCS	57	78	0.823 (0.742-0.905)	2.051	0.737	0.859	0.792	0.817	0.596

Abbreviations: ROC, Receiver Operator Characteristic curve; AUC, Area Under Curve; CI, Confidence Interval; YI, Youden's index.

Supplementary Table 2. Details of the prognostic K-M analysis of GPER1 in pan-cancer.

Cancer	N	HR (95% CI)	P value
ACC	79	0.64(0.30-1.36)	0.243
BLCA	433	1.19(0.89-1.59)	0.243
**BRCA**	**1090**	**0.69(0.50-0.97)**	**0.03**
CESC	309	1.04(0.66-1.65)	0.865
CHOL	45	0.70(0.27-1.85)	0.473
COAD	521	1.21(0.82-1.79)	0.33
**DLBC**	**48**	**0.09(0.01-0.79)**	**0.029**
**ESCA**	**173**	**0.46(0.28-0.77)**	**0.003**
GBM	174	0.90(0.64-1.27)	0.548
**HNSC**	**500**	**0.75(0.57-0.99)**	**0.042**
KICH	89	1.30(0.35-4.83)	0.698
**KIRC**	**611**	**0.59(0.44-0.80)**	**0.001**
**KIRP**	**288**	**0.45(0.25-0.81)**	**0.008**
LAML	151	1.18(0.78-1.80)	0.436
LGG	529	0.75(0.54-1.05)	0.093
LIHC	424	1.04(0.74-1.47)	0.825
**LUAD**	**513**	**0.71(0.52-0.98)**	**0.036**
LUSC	551	1.03(0.79-1.35)	0.826
MESO	86	0.69(0.43-1.11)	0.131
OS	101	1.26(0.68-2.35)	0.462
OSCC	361	0.93(0.67-1.29)	0.675
OV	379	1.16(0.89-1.50)	0.263
**PAAD**	**177**	**0.59(0.38-0.93)**	**0.022**
PCPG	186	0.63(0.15-2.65)	0.531
PRAD	551	0.78(0.22-2.80)	0.703
READ	177	2.15(0.93-4.95)	0.073
SKCM	472	1.04(0.80-1.36)	0.764
**SARC**	**259**	**0.38(0.20-0.70)**	**0.002**
**STAD**	**375**	**1.50(1.06-2.12)**	**0.023**
TGCT	156	1.24(0.17-9.35)	0.833
THCA	568	1.72(0.64-4.65)	0.284
THYM	121	0.52(0.13-2.09)	0.359
**UCEC**	**587**	**0.53(0.34-0.80)**	**0.003**
UCS	56	1.10(0.54-2.21)	0.798
UVM	80	1.52(0.67-3.47)	0.321
