# Supplementary material for: Utility of G protein-coupled oestrogen receptor 1 as a biomarker for pan-cancer diagnosis, prognosis and immune infiltration: a comprehensive bioinformatics analysis
Source: Aging (Albany NY). 2023 Nov 2;15(21):12021–67. doi: 10.18632/aging.205162 (PMC10683611; doi:10.18632/aging.205162)
Supplement: Supplementary Table 3 [file aging-15-205162-s003.docx]

**Supplementary Table 3. Top up and down 30 items of differential expressed genes of GPER1 in different cancers.**

| **Cancer** | **gene_name** | **log2FoldChange** | **padj** |  | **Cancer** | **gene_name** | **log2FoldChange** | **padj** |
| --- | --- | --- | --- | --- | --- | --- | --- | --- |
|  | **UP 30** |  |  |  |  | **DOWN 30** |  |  |
| **BRCA** | CHGB | 5.679703 | 2.2E-172 |  | BRCA | CSN2 | -6.77626 | 5.19E-41 |
|  | CARTPT | 5.154376 | 6.24E-41 |  |  | LALBA | -6.07296 | 6.3E-44 |
|  | CHGA | 4.436433 | 1.07E-98 |  |  | SMR3B | -4.71073 | 3.63E-42 |
|  | CPB1 | 3.479031 | 2.78E-53 |  |  | MAGEA10 | -3.70815 | 3.51E-26 |
|  | MUC2 | 3.393907 | 9.49E-52 |  |  | FGG | -3.67016 | 1.21E-45 |
|  | TRH | 3.203478 | 9.84E-66 |  |  | RN7SKP255 | -3.62787 | 6.23E-16 |
|  | PCSK1 | 3.149439 | 9.34E-89 |  |  | GNAT3 | -3.52968 | 5.71E-19 |
|  | AMER3 | 3.074475 | 9.07E-40 |  |  | RNU1-11P | -3.48795 | 1.02E-11 |
|  | CPLX2 | 2.894319 | 6.74E-25 |  |  | MAGEA4 | -3.45253 | 6.48E-21 |
|  | NTS | 2.845586 | 2.1E-30 |  |  | SMR3A | -3.38533 | 2.47E-10 |
|  | AC092979.1 | 2.805325 | 3.45E-24 |  |  | ZFP42 | -3.34346 | 1.48E-46 |
|  | CLVS2 | 2.786592 | 9.6E-43 |  |  | CSN3 | -3.33679 | 5.29E-19 |
|  | LRRC53 | 2.643618 | 4.37E-20 |  |  | SNORA74B | -3.09601 | 5.38E-58 |
|  | NEUROD1 | 2.613162 | 1.03E-09 |  |  | RN7SL3 | -3.08754 | 1.33E-76 |
|  | AL591034.1 | 2.598169 | 3.73E-13 |  |  | AC111149.2 | -3.07608 | 3.7E-14 |
|  | SEZ6 | 2.597792 | 2.54E-56 |  |  | LINC02169 | -3.02112 | 4.37E-29 |
|  | KRT1 | 2.438858 | 2.47E-47 |  |  | RN7SKP203 | -2.94823 | 2.5E-07 |
|  | RN7SL255P | 2.426905 | 3.49E-07 |  |  | SCARNA5 | -2.91809 | 1.14E-58 |
|  | AC073525.1 | 2.425074 | 2.23E-17 |  |  | AF241725.1 | -2.879 | 0.006302 |
|  | GRIA1 | 2.364418 | 7.12E-41 |  |  | RTP3 | -2.76834 | 3.74E-24 |
|  | RNA5SP107 | 2.363721 | 4.38E-05 |  |  | FGA | -2.7253 | 4.83E-17 |
|  | LEP | 2.306479 | 1.37E-46 |  |  | ORM2 | -2.65616 | 2.72E-52 |
|  | XKR7 | 2.296579 | 7.71E-39 |  |  | SELENOOLP | -2.62032 | 7.01E-08 |
|  | AC104407.1 | 2.270919 | 4.03E-17 |  |  | FABP7 | -2.60336 | 9.65E-34 |
|  | ARHGAP36 | 2.232731 | 3.65E-22 |  |  | SNORD15B | -2.60281 | 2.23E-76 |
|  | SLC22A12 | 2.230705 | 1.12E-22 |  |  | PRR27 | -2.59172 | 6.25E-10 |
|  | MYF6 | 2.21945 | 3.44E-20 |  |  | MAGEA3 | -2.57968 | 1.48E-09 |
|  | UCP1 | 2.195208 | 2.71E-34 |  |  | ORM1 | -2.56547 | 1.17E-41 |
|  | MYL1 | 2.135403 | 3.73E-05 |  |  | SNORA73B | -2.56232 | 1.58E-78 |
|  | RN7SKP256 | 2.113286 | 5.52E-06 |  |  | LBP | -2.52213 | 1.72E-52 |
|  |  |  |  |  |  |  |  |  |
| **DLBC** | IGKV1D-16 | 4.02296 | 6.07E-07 |  |  | MAGEB1 | -9.67615 | 1.68E-12 |
|  | FAM9C | 4.020583 | 1.06E-05 |  |  | MAGEA4 | -8.57687 | 2.26E-06 |
|  | IGHV7-34-1 | 3.741337 | 0.000294 |  |  | CT45A10 | -6.57595 | 2.09E-05 |
|  | AC091729.1 | 3.636869 | 8.35E-08 |  |  | AC073365.1 | -6.2522 | 0.00024 |
|  | IGLC6 | 3.559263 | 1.39E-07 |  |  | CT45A1 | -6.14872 | 0.000165 |
|  | LRP1B | 3.481076 | 0.000535 |  |  | LINC01307 | -6.13801 | 1.71E-06 |
|  | CCDC54 | 3.28575 | 0.000115 |  |  | PSORS1C2 | -6.03402 | 2.51E-10 |
|  | KBTBD12 | 3.25178 | 0.00041 |  |  | SLC5A12 | -4.84734 | 2.92E-06 |
|  | TNNT2 | 3.203985 | 1.27E-08 |  |  | CBY2 | -4.78559 | 1.79E-05 |
|  | LINC01239 | 3.180682 | 0.000921 |  |  | SLCO6A1 | -4.75205 | 1.33E-05 |
|  | MMP20 | 3.157434 | 0.002665 |  |  | LINC00858 | -4.53735 | 0.000481 |
|  | IL6-AS1 | 3.044837 | 7.9E-06 |  |  | C7orf33 | -4.4594 | 0.002018 |
|  | IL12B | 3.018359 | 1.72E-05 |  |  | FAR2P1 | -4.39035 | 8.32E-08 |
|  | MTRNR2L1 | 2.985345 | 0.001977 |  |  | AC005993.1 | -4.38502 | 0.004446 |
|  | AP005901.1 | 2.910931 | 0.000279 |  |  | LINC00491 | -4.33432 | 0.00212 |
|  | ARHGDIG | 2.897678 | 6.73E-05 |  |  | ACOD1 | -4.27085 | 2.91E-08 |
|  | AC019118.2 | 2.895929 | 0.00165 |  |  | AC011444.2 | -4.22817 | 2.9E-06 |
|  | IGLC7 | 2.852366 | 0.000123 |  |  | MAPK8IP1P2 | -4.04252 | 0.000828 |
|  | LINC00525 | 2.843114 | 0.001479 |  |  | PRSS21 | -3.84233 | 4.49E-11 |
|  | LINC01551 | 2.839816 | 0.000328 |  |  | CTXN3 | -3.8246 | 0.001302 |
|  | LINC00870 | 2.828969 | 0.002071 |  |  | AC007614.1 | -3.67546 | 0.002846 |
|  | ATP4B | 2.825603 | 0.00203 |  |  | MAPK8IP1P1 | -3.67213 | 0.005983 |
|  | AL122058.1 | 2.786671 | 0.000588 |  |  | CT45A3 | -3.63713 | 0.007667 |
|  | IGHV3OR16-10 | 2.781018 | 0.005247 |  |  | PLCZ1 | -3.45451 | 0.006227 |
|  | BMP7 | 2.765341 | 5.07E-05 |  |  | H2AB1 | -3.40522 | 0.000412 |
|  | CAMTA1-AS1 | 2.73883 | 0.002617 |  |  | CTAG2 | -3.38572 | 0.00074 |
|  | AC097065.2 | 2.711772 | 0.001224 |  |  | CRYM | -3.37788 | 1.49E-06 |
|  | LINC01697 | 2.704711 | 0.001338 |  |  | AC079160.1 | -3.3619 | 0.001466 |
|  | AC022483.3 | 2.696973 | 0.00985 |  |  | ZNF663P | -3.28797 | 0.00041 |
|  | AL355994.2 | 2.680885 | 0.006321 |  |  | DMRT2 | -3.25044 | 1.5E-08 |
|  |  |  |  |  |  |  |  |  |
| **ESCA** | PGA5 | 4.974989 | 1.32E-13 |  |  | MUC12 | -3.45197 | 2.54E-16 |
|  | DANT1 | 3.961629 | 0.009351 |  |  | Y_RNA | -3.36093 | 6.55E-06 |
|  | PGA3 | 3.673479 | 2.19E-05 |  |  | CPS1 | -3.2751 | 2.48E-12 |
|  | GKN1 | 3.592565 | 2.11E-08 |  |  | ITLN1 | -2.87599 | 4.23E-07 |
|  | GC | 3.559419 | 6.89E-06 |  |  | FGF3 | -2.74355 | 0.002664 |
|  | GHRH | 3.476339 | 6.86E-06 |  |  | AC125603.2 | -2.59851 | 9.56E-05 |
|  | ATP4B | 3.377648 | 1.12E-09 |  |  | CELP | -2.27915 | 1.25E-06 |
|  | LIPF | 3.317859 | 1.88E-05 |  |  | AC083849.1 | -2.27392 | 0.006437 |
|  | AC009831.2 | 3.292908 | 6.34E-05 |  |  | NFE4 | -2.26029 | 2.57E-07 |
|  | GRIK3 | 3.27007 | 2.54E-16 |  |  | SLC26A3 | -2.18405 | 0.000231 |
|  | MAGEC2 | 3.268042 | 0.000356 |  |  | CCNG1P1 | -2.12854 | 0.002569 |
|  | AC074389.2 | 3.1902 | 4.36E-06 |  |  | C17orf77 | -2.10059 | 0.001344 |
|  | AL161716.1 | 3.120386 | 0.002653 |  |  | LINC01648 | -2.09206 | 0.002557 |
|  | MYOC | 3.068908 | 1.18E-08 |  |  | PCK1 | -2.07691 | 0.000333 |
|  | GABRA5 | 3.049095 | 3.12E-08 |  |  | LGSN | -2.07591 | 0.000841 |
|  | CDH18 | 3.030286 | 1.11E-09 |  |  | AC254629.1 | -2.00593 | 0.000407 |
|  | BRINP3 | 2.989396 | 4.05E-08 |  |  | KRT40 | -1.95902 | 0.00018 |
|  | PGC | 2.972182 | 4.81E-05 |  |  | AC116345.1 | -1.95709 | 0.000338 |
|  | ATP4A | 2.934617 | 1.02E-10 |  |  | S100A12 | -1.95656 | 8.38E-05 |
|  | NRXN1 | 2.883713 | 6.69E-14 |  |  | GSTA8P | -1.89317 | 0.000407 |
|  | ASTN1 | 2.812478 | 5.71E-12 |  |  | MTTP | -1.87544 | 0.000262 |
|  | KCTD8 | 2.798903 | 1.39E-09 |  |  | CPA2 | -1.85303 | 0.000685 |
|  | CHIA | 2.783089 | 0.000715 |  |  | ACRP1 | -1.82137 | 0.00296 |
|  | AC106729.1 | 2.764067 | 0.000807 |  |  | UGT1A8 | -1.81561 | 0.0004 |
|  | GH2 | 2.754326 | 0.003788 |  |  | AADACL2 | -1.79219 | 0.001112 |
|  | SCGB3A1 | 2.739665 | 3.09E-08 |  |  | ACHE | -1.75669 | 2.27E-05 |
|  | XKR4 | 2.738651 | 2.59E-13 |  |  | MALRD1 | -1.74589 | 0.000261 |
|  | DES | 2.592838 | 2.91E-08 |  |  | HSD3B2 | -1.74167 | 0.006869 |
|  | GADL1 | 2.56807 | 2.78E-07 |  |  | OR7E161P | -1.72516 | 0.000439 |
|  | MYL7 | 2.566134 | 2.53E-06 |  |  | PPP1R1B | -1.70864 | 0.007047 |
|  |  |  |  |  |  |  |  |  |
| **HNSC** | LINC01820 | 4.445306 | 3.35E-19 |  |  | SPINK7 | -2.35355 | 6.43E-18 |
|  | LINC02525 | 4.256483 | 1.82E-20 |  |  | DEFB103A | -2.32595 | 6.15E-08 |
|  | BOK-AS1 | 4.190943 | 4.46E-35 |  |  | KRT8P6 | -2.13943 | 2.24E-07 |
|  | LINC02583 | 4.180457 | 8.78E-13 |  |  | AC129492.6 | -2.13874 | 1.35E-18 |
|  | AC092916.1 | 4.131718 | 9.46E-13 |  |  | KRT2 | -2.08278 | 1.62E-13 |
|  | PAGE2 | 3.610667 | 2.22E-11 |  |  | LCE3B | -2.06553 | 0.000251 |
|  | PDIA2 | 3.353922 | 8.49E-51 |  |  | KRT24 | -2.02499 | 1.06E-10 |
|  | NR5A1 | 3.349581 | 2E-24 |  |  | SERPINB12 | -2.0012 | 4.81E-15 |
|  | AL162427.1 | 3.08942 | 9.22E-12 |  |  | FLG2 | -1.96096 | 4.91E-10 |
|  | CLDN19 | 3.082975 | 2.25E-32 |  |  | FABP4 | -1.95264 | 3.02E-12 |
|  | TDRD15 | 3.081994 | 3.55E-10 |  |  | AL513304.1 | -1.89947 | 2.07E-12 |
|  | LINC01854 | 3.006034 | 1.75E-15 |  |  | TRIM40 | -1.85819 | 1.9E-11 |
|  | AC106875.1 | 2.981822 | 1.45E-11 |  |  | TRIM49B | -1.82267 | 9.42E-05 |
|  | SPDYC | 2.880291 | 4.5E-22 |  |  | USP17L1 | -1.82237 | 1.7E-06 |
|  | AF279873.3 | 2.866091 | 1.19E-12 |  |  | KRT8P40 | -1.76748 | 1.75E-09 |
|  | AC025254.1 | 2.838769 | 1.14E-07 |  |  | CLDN17 | -1.74963 | 7.09E-10 |
|  | FOXH1 | 2.797984 | 1.94E-37 |  |  | HRNR | -1.74234 | 6.12E-20 |
|  | AC004875.1 | 2.724289 | 9.48E-06 |  |  | KRT37 | -1.7409 | 1.23E-14 |
|  | HCAR1 | 2.716259 | 6.25E-36 |  |  | SPINK6 | -1.73011 | 2.69E-09 |
|  | SPATA21 | 2.713204 | 5.11E-22 |  |  | LCE2D | -1.72275 | 1.65E-06 |
|  | PTX4 | 2.671537 | 1.43E-19 |  |  | TGM3 | -1.69241 | 1.98E-09 |
|  | AL139042.1 | 2.668375 | 0.000561 |  |  | SLURP1 | -1.64514 | 5.76E-11 |
|  | TDRD1 | 2.667108 | 5.22E-33 |  |  | AC008663.1 | -1.64401 | 1.17E-11 |
|  | PNMA5 | 2.627251 | 5.48E-23 |  |  | AC083841.1 | -1.62355 | 1.96E-12 |
|  | CALHM1 | 2.601694 | 1.05E-27 |  |  | DEFB4A | -1.62001 | 3.01E-08 |
|  | AC074389.2 | 2.567982 | 5.29E-06 |  |  | C7orf33 | -1.60312 | 0.000335 |
|  | AC092127.2 | 2.53823 | 4.38E-16 |  |  | CRNN | -1.58789 | 4.72E-07 |
|  | FIBCD1 | 2.51764 | 1.1E-30 |  |  | PLA2G4D | -1.57733 | 2.41E-12 |
|  | AC084357.3 | 2.512317 | 7.31E-14 |  |  | PHBP16 | -1.56481 | 6.01E-07 |
|  | NFE4 | 2.466176 | 5.11E-22 |  |  | KPRP | -1.56438 | 5.92E-10 |
|  |  |  |  |  |  |  |  |  |
| **KIRC** | AC026369.1 | 2.333746 | 7.8E-34 |  |  | LINC01802 | -5.80041 | 1.25E-29 |
|  | SLC13A2 | 2.141196 | 1.81E-21 |  |  | HEPACAM2 | -5.36748 | 7.47E-67 |
|  | UMOD | 2.120776 | 2.75E-11 |  |  | RNY3 | -5.33556 | 9.66E-07 |
|  | PLG | 2.061276 | 2.98E-14 |  |  | AC010776.3 | -5.28553 | 6.86E-16 |
|  | AC091729.2 | 1.947709 | 1.52E-31 |  |  | RHCG | -5.23437 | 8.44E-75 |
|  | REN | 1.865768 | 5.37E-16 |  |  | FOXI1 | -5.10487 | 1.49E-38 |
|  | SLC22A8 | 1.859449 | 1.43E-14 |  |  | KLK1 | -5.05568 | 4.85E-66 |
|  | IQSEC3 | 1.852744 | 2.16E-30 |  |  | KLK4 | -4.88956 | 9.2E-50 |
|  | AL354761.1 | 1.839688 | 0.00084 |  |  | AC026888.1 | -4.83689 | 9.14E-12 |
|  | NKX2-3 | 1.821784 | 2.36E-10 |  |  | AC015910.1 | -4.82578 | 1.57E-56 |
|  | SLC34A1 | 1.801698 | 8.97E-15 |  |  | CASP14 | -4.80444 | 3.05E-27 |
|  | AC087379.2 | 1.795259 | 1.39E-14 |  |  | ATP6V1G3 | -4.79953 | 1.62E-18 |
|  | SLC6A19 | 1.785883 | 8.33E-14 |  |  | LINC01187 | -4.78768 | 1.1E-49 |
|  | AC025575.2 | 1.770251 | 0.000165 |  |  | CLDN8 | -4.68223 | 2.5E-31 |
|  | AC026396.1 | 1.751408 | 1.09E-16 |  |  | AC118345.1 | -4.67353 | 4.6E-08 |
|  | G6PC | 1.736606 | 9.35E-17 |  |  | LINC01230 | -4.65788 | 1.83E-35 |
|  | KRT20 | 1.711726 | 6.76E-11 |  |  | RTL1 | -4.60625 | 3.29E-36 |
|  | AC245519.1 | 1.70814 | 2.73E-16 |  |  | AC010776.2 | -4.58368 | 1.11E-27 |
|  | MAPT-AS1 | 1.659387 | 1.25E-17 |  |  | LRRTM1 | -4.49483 | 5.33E-30 |
|  | CD8B2 | 1.658602 | 1.5E-12 |  |  | LHX9 | -4.48315 | 2.43E-56 |
|  | AC243829.5 | 1.657908 | 0.002425 |  |  | LINC00588 | -4.43978 | 2.7E-11 |
|  | AC087379.1 | 1.604515 | 4.17E-06 |  |  | TMPRSS11E | -4.32054 | 1.74E-42 |
|  | TRIM63 | 1.59371 | 1.81E-15 |  |  | DMRT2 | -4.31106 | 2E-47 |
|  | ALDOB | 1.588953 | 2.88E-13 |  |  | PART1 | -4.30789 | 2.18E-58 |
|  | MYH8 | 1.585164 | 3.01E-07 |  |  | LINC01612 | -4.17037 | 1.89E-22 |
|  | MS4A10 | 1.583622 | 3.58E-09 |  |  | FDCSP | -4.16584 | 8.56E-35 |
|  | CHRNA4 | 1.567504 | 4.05E-09 |  |  | INSYN1-AS1 | -4.14829 | 9.43E-37 |
|  | C14orf180 | 1.563322 | 1.95E-11 |  |  | Z82185.1 | -4.14316 | 3.88E-09 |
|  | SLC22A9 | 1.558219 | 1.84E-12 |  |  | ODAM | -4.13388 | 7.9E-19 |
|  | LPA | 1.551674 | 1.34E-14 |  |  | KLK15 | -4.11396 | 5.1E-17 |
|  |  |  |  |  |  |  |  |  |
| **KIRP** | AC006960.2 | 3.770621 | 1.03E-09 |  |  | MAGEC2 | -7.12796 | 2.92E-10 |
|  | ADGRA1 | 3.227046 | 2.2E-16 |  |  | ZIC2 | -5.71988 | 6.14E-33 |
|  | ADGRA1-AS1 | 2.639541 | 6.48E-08 |  |  | PAGE1 | -5.53015 | 1.04E-08 |
|  | AC106754.1 | 2.549595 | 0.004098 |  |  | SCNN1G | -5.305 | 1.45E-32 |
|  | AL390786.1 | 2.507955 | 6.53E-06 |  |  | CASP14 | -5.24717 | 4.53E-13 |
|  | RNU1-65P | 2.349311 | 0.000606 |  |  | GPR87 | -5.2195 | 1.26E-38 |
|  | SLC30A8 | 2.16378 | 7.48E-09 |  |  | ALDH3B2 | -4.94699 | 1.59E-31 |
|  | SRRM4 | 2.154214 | 1.05E-13 |  |  | SLITRK6 | -4.8328 | 3.12E-17 |
|  | PRDM14 | 2.109059 | 1.08E-07 |  |  | GATA3-AS1 | -4.82458 | 7.58E-23 |
|  | NEU4 | 2.056326 | 5.4E-13 |  |  | MAGEA3 | -4.80024 | 3.14E-07 |
|  | SORCS1 | 1.984952 | 5.06E-08 |  |  | TFAP2A | -4.61386 | 1.05E-66 |
|  | KLK3 | 1.957505 | 0.000193 |  |  | TMPRSS4 | -4.58751 | 8.19E-43 |
|  | AL021331.1 | 1.897637 | 1.8E-15 |  |  | MAGEC1 | -4.53085 | 3.88E-06 |
|  | DPEP1 | 1.88911 | 4.47E-14 |  |  | MIR200CHG | -4.48588 | 4.47E-14 |
|  | RNU7-84P | 1.855812 | 8.12E-10 |  |  | HP | -4.46265 | 5.94E-36 |
|  | AC111000.1 | 1.832738 | 3.02E-08 |  |  | PROM2 | -4.4614 | 2.86E-60 |
|  | AC093627.2 | 1.806645 | 0.000557 |  |  | NR0B2 | -4.34994 | 4.24E-34 |
|  | HMGN2P8 | 1.787473 | 3.98E-06 |  |  | PAEP | -4.34544 | 1.37E-19 |
|  | GRIA4 | 1.776642 | 5.97E-07 |  |  | KLK6 | -4.2441 | 1.6E-28 |
|  | SOSTDC1 | 1.772019 | 1.74E-07 |  |  | LINC01606 | -4.17931 | 4.01E-12 |
|  | CYSLTR2 | 1.762683 | 5.96E-09 |  |  | FAM230C | -4.13453 | 0.000207 |
|  | CAMKV | 1.75502 | 1.4E-07 |  |  | TFF1 | -4.13177 | 9.33E-10 |
|  | RNU6ATAC40P | 1.752628 | 7.1E-16 |  |  | TCN1 | -4.1158 | 3.38E-49 |
|  | CKS1BP3 | 1.751759 | 0.001125 |  |  | CRP | -4.09057 | 1.1E-27 |
|  | FTCD-AS1 | 1.747456 | 1.53E-08 |  |  | ERVE-1 | -4.039 | 5.79E-12 |
|  | LRIT2 | 1.745183 | 1.58E-07 |  |  | ZIC5 | -4.03456 | 4.29E-10 |
|  | AL160191.1 | 1.743061 | 0.000862 |  |  | PSCA | -3.98742 | 2.32E-38 |
|  | OXT | 1.739147 | 6.8E-08 |  |  | GRHL2 | -3.96695 | 7.38E-28 |
|  | MYH8 | 1.737142 | 1.06E-06 |  |  | CLDN8 | -3.95683 | 1.82E-13 |
|  | TDRD1 | 1.735415 | 1.12E-06 |  |  | CYP4F8 | -3.89238 | 3.95E-14 |
|  |  |  |  |  |  |  |  |  |
| **LUAD** | MUC17 | 4.519871 | 3.85E-22 |  |  | CGA | -4.43785 | 8.69E-35 |
|  | TFF2 | 4.204987 | 1.18E-21 |  |  | CALCA | -4.32752 | 3.08E-34 |
|  | SNORA54 | 4.1498 | 2.44E-34 |  |  | KLK14 | -4.1144 | 3.37E-76 |
|  | RN7SKP227 | 4.14822 | 0.001121 |  |  | UGT3A1 | -3.97433 | 5.37E-19 |
|  | REG4 | 4.087856 | 7.67E-35 |  |  | SPAG11B | -3.89068 | 3.88E-06 |
|  | MTCYBP32 | 3.745449 | 1.06E-05 |  |  | OTX2 | -3.79483 | 5.49E-18 |
|  | Y_RNA | 3.744401 | 0.000204 |  |  | KLK12 | -3.65999 | 2.53E-31 |
|  | ONECUT3 | 3.603643 | 1.08E-36 |  |  | SST | -3.54398 | 1.86E-14 |
|  | AC136424.1 | 3.568508 | 0.00033 |  |  | AC120498.7 | -3.51599 | 9.07E-08 |
|  | MIR3609 | 3.508831 | 4.56E-23 |  |  | LINC01886 | -3.4986 | 1.45E-12 |
|  | EIF4E2P1 | 3.435628 | 1.09E-05 |  |  | PSG4 | -3.35395 | 1.52E-14 |
|  | RNU2-23P | 3.423645 | 0.008142 |  |  | MAGEA4 | -3.3486 | 4.32E-09 |
|  | RNU6-373P | 3.416804 | 2.88E-05 |  |  | PAGE4 | -3.31505 | 3.15E-15 |
|  | AC136424.2 | 3.41357 | 1.61E-08 |  |  | ZMAT4 | -3.24426 | 9.09E-30 |
|  | SNORA23 | 3.409439 | 3.71E-39 |  |  | AF274573.1 | -3.14217 | 1.44E-11 |
|  | RNU6-433P | 3.408781 | 2.41E-05 |  |  | AC083809.1 | -3.10478 | 2.99E-33 |
|  | RNU6-522P | 3.397323 | 0.000316 |  |  | CALML5 | -3.09921 | 1.11E-17 |
|  | AC079449.1 | 3.352772 | 1.55E-05 |  |  | KLK13 | -3.07987 | 3.02E-40 |
|  | AL445523.1 | 3.349121 | 5.42E-05 |  |  | SLC38A8 | -3.03667 | 6.77E-30 |
|  | RMRP | 3.33828 | 7.27E-13 |  |  | NTS | -3.02332 | 3.64E-23 |
|  | RNVU1-2A | 3.32747 | 9.12E-05 |  |  | AC093903.1 | -3.00078 | 3.92E-13 |
|  | PIGFP2 | 3.32328 | 0.003746 |  |  | DEFA9P | -2.97394 | 2.13E-05 |
|  | TCF4-AS2 | 3.319599 | 0.000388 |  |  | BPIFA1 | -2.96263 | 1.54E-24 |
|  | AC120349.2 | 3.319475 | 4.77E-07 |  |  | GAGE2A | -2.9252 | 0.000737 |
|  | RNU4-87P | 3.299831 | 5.4E-05 |  |  | LINC01896 | -2.91877 | 3.56E-06 |
|  | AL360219.1 | 3.282207 | 1.79E-06 |  |  | FGB | -2.90323 | 1.09E-16 |
|  | AP002453.1 | 3.272121 | 0.000154 |  |  | AC114786.2 | -2.90254 | 5.45E-07 |
|  | AC008873.1 | 3.248509 | 0.000393 |  |  | NLRP5 | -2.8985 | 1.71E-10 |
|  | AC110603.1 | 3.235379 | 0.007956 |  |  | LINC02037 | -2.88363 | 3.2E-15 |
|  | MIR3140 | 3.230778 | 8.38E-05 |  |  | DEFA8P | -2.86171 | 0.000102 |
|  |  |  |  |  |  |  |  |  |
| **PAAD** | GAST | 5.295368 | 1.98E-23 |  |  | CLPS | -3.93196 | 5.99E-10 |
|  | LINC02616 | 3.814449 | 0.004668 |  |  | AMY2A | -3.73366 | 9.41E-08 |
|  | C6orf58 | 3.351175 | 6.78E-14 |  |  | PNLIP | -3.71655 | 2.42E-05 |
|  | DEFA6 | 3.163035 | 0.00056 |  |  | SYCN | -3.57753 | 2.24E-07 |
|  | SLC10A2 | 3.025845 | 1.5E-06 |  |  | CELA2A | -3.5742 | 2.5E-08 |
|  | PAX7 | 2.894427 | 0.000333 |  |  | PLA2G1B | -3.49531 | 4.73E-09 |
|  | SMYD1 | 2.76112 | 3.05E-05 |  |  | AMY1B | -3.47128 | 9.1E-06 |
|  | PART1 | 2.59148 | 2.57E-13 |  |  | CEL | -3.31764 | 5.14E-09 |
|  | EVX1-AS | 2.53582 | 9.42E-06 |  |  | PRSS1 | -3.29916 | 3.33E-09 |
|  | PAX3 | 2.391012 | 0.000343 |  |  | CPA1 | -3.24249 | 2.2E-05 |
|  | LINC01602 | 2.385376 | 0.000189 |  |  | CELA3B | -3.22709 | 4.8E-07 |
|  | TCL1A | 2.337213 | 3.23E-05 |  |  | GPHA2 | -3.03068 | 7.07E-09 |
|  | FCER2 | 2.25079 | 6.6E-06 |  |  | CTRL | -2.98589 | 1.87E-12 |
|  | AL161781.2 | 2.214891 | 0.000704 |  |  | CELA3A | -2.96249 | 7.44E-06 |
|  | LRRC53 | 2.186798 | 0.006359 |  |  | AQP8 | -2.93905 | 4.8E-07 |
|  | LINC01082 | 2.175603 | 0.001987 |  |  | CPA2 | -2.86169 | 1.15E-05 |
|  | AC091891.1 | 2.122423 | 0.008614 |  |  | CPB1 | -2.85445 | 1.09E-05 |
|  | SHISA8 | 2.109999 | 2.11E-07 |  |  | RBPJL | -2.81167 | 1.93E-06 |
|  | AC010442.2 | 2.067253 | 0.003424 |  |  | TMED11P | -2.81075 | 1.43E-05 |
|  | HOXC12 | 2.054466 | 0.009056 |  |  | CUZD1 | -2.80184 | 4.72E-09 |
|  | DUSP27 | 2.050722 | 4.99E-05 |  |  | SERPINI2 | -2.79908 | 5.3E-06 |
|  | FCAMR | 2.012047 | 9.13E-08 |  |  | GUCA1C | -2.79841 | 0.00031 |
|  | SERPINA9 | 1.998499 | 2.2E-05 |  |  | MIR208B | -2.7923 | 0.000844 |
|  | CPLX2 | 1.945624 | 1.33E-05 |  |  | CTRC | -2.77358 | 6.61E-06 |
|  | KRT16P6 | 1.898161 | 0.000787 |  |  | INSL4 | -2.76615 | 4.78E-06 |
|  | AC078864.1 | 1.889646 | 0.000874 |  |  | CTRB2 | -2.742 | 5.78E-05 |
|  | VPREB3 | 1.887945 | 4.92E-06 |  |  | CALML3 | -2.73861 | 1.47E-06 |
|  | TENM1 | 1.886258 | 4.03E-09 |  |  | MYBPC1 | -2.73334 | 4.4E-08 |
|  | C1QTNF8 | 1.880201 | 0.002935 |  |  | RN7SL386P | -2.73245 | 9.97E-05 |
|  | AC097641.1 | 1.8399 | 0.001862 |  |  | CELP | -2.72686 | 9.82E-07 |
|  |  |  |  |  |  |  |  |  |
| **SARC** | CAPS2-AS1 | 4.41408 | 4.14E-09 |  |  | AC015969.1 | -4.41953 | 6.86E-13 |
|  | LRRC10 | 3.65913 | 4.9E-05 |  |  | CACNG5 | -3.99629 | 3.55E-13 |
|  | ADIPOQ | 3.628391 | 3.98E-08 |  |  | ACTBP12 | -3.81663 | 2.55E-26 |
|  | CYP2W1 | 3.602179 | 2.43E-19 |  |  | SFTPA1 | -3.74198 | 1.02E-05 |
|  | AC123904.1 | 3.449882 | 0.00118 |  |  | MAGEA4 | -3.67298 | 1.35E-05 |
|  | AL591896.1 | 3.443899 | 7.66E-07 |  |  | AF241725.1 | -3.6709 | 3.93E-05 |
|  | LY6G6F-LY6G6D | 3.382348 | 8.11E-06 |  |  | FGF19 | -3.47454 | 5.16E-07 |
|  | ARHGAP36 | 3.372745 | 3.76E-11 |  |  | ACTA1 | -3.45669 | 3.65E-16 |
|  | CSN1S1 | 3.361365 | 5.8E-05 |  |  | TAC3 | -3.44041 | 2.6E-18 |
|  | AADACL2 | 3.288065 | 1.21E-07 |  |  | AL392003.2 | -3.43614 | 7.54E-08 |
|  | CGB5 | 3.19638 | 0.004007 |  |  | AL356364.1 | -3.4254 | 1.46E-05 |
|  | SPRR2G | 3.055855 | 0.000125 |  |  | LHX3 | -3.3564 | 3.61E-09 |
|  | DLK1 | 3.010789 | 2.37E-06 |  |  | ELAVL3 | -3.28142 | 3.65E-16 |
|  | AL035706.1 | 3.0006 | 2.07E-05 |  |  | RNU7-165P | -3.19346 | 0.000637 |
|  | BPIFB4 | 2.951367 | 5.9E-07 |  |  | SMYD1 | -3.17696 | 5.14E-06 |
|  | SLC7A10 | 2.906408 | 7.08E-13 |  |  | SPANXD | -3.1286 | 0.002457 |
|  | MRGPRX7P | 2.901919 | 0.001172 |  |  | SHH | -3.12708 | 1.1E-11 |
|  | FLJ16779 | 2.839997 | 2.24E-10 |  |  | SFTPB | -3.07247 | 2.21E-16 |
|  | LINC00645 | 2.818842 | 4.48E-05 |  |  | CBLN1 | -3.06412 | 5.41E-13 |
|  | GVINP2 | 2.808075 | 1.15E-10 |  |  | OR52K3P | -3.05888 | 1.13E-18 |
|  | GRIK1 | 2.799981 | 8.09E-23 |  |  | PRLHR | -3.00816 | 7.66E-07 |
|  | AL022318.1 | 2.752948 | 6.51E-11 |  |  | GPR87 | -2.9632 | 7.59E-11 |
|  | KRT74 | 2.752492 | 0.008833 |  |  | PRR32 | -2.93107 | 3.85E-07 |
|  | CNTNAP4 | 2.708938 | 5.55E-08 |  |  | AC069155.1 | -2.91859 | 3.54E-06 |
|  | DUOXA2 | 2.70883 | 2.8E-10 |  |  | CCDC190 | -2.91543 | 9.23E-12 |
|  | MMP27 | 2.697422 | 1.09E-06 |  |  | MAGEA4-AS1 | -2.89343 | 0.006272 |
|  | AL359924.1 | 2.688201 | 0.004437 |  |  | AL035425.3 | -2.87621 | 1.25E-05 |
|  | CRYGD | 2.665713 | 0.002744 |  |  | AC025048.1 | -2.84667 | 1.23E-12 |
|  | CTD-2297D10.2 | 2.661669 | 2.06E-07 |  |  | MUC6 | -2.84283 | 7.78E-18 |
|  | FAM238B | 2.638462 | 0.000112 |  |  | AL390719.2 | -2.82342 | 6.43E-06 |
|  |  |  |  |  |  |  |  |  |
| **STAD** | SMYD1 | 5.060315 | 9.4E-64 |  |  | KRT6C | -4.19653 | 4.22E-26 |
|  | PGA4 | 4.145076 | 2.34E-09 |  |  | SPRR2E | -4.1428 | 1.59E-13 |
|  | ACTG2 | 4.025859 | 8.74E-73 |  |  | CRNN | -4.02888 | 3.03E-10 |
|  | DES | 3.99453 | 1.47E-54 |  |  | PNLIP | -4.01644 | 1.11E-16 |
|  | KCNA1 | 3.928379 | 7.26E-42 |  |  | SPRR2B | -3.71862 | 1.46E-05 |
|  | PGA5 | 3.892619 | 4.78E-22 |  |  | MUC21 | -3.40624 | 6.3E-19 |
|  | AC093787.1 | 3.847605 | 9.78E-31 |  |  | IL36A | -3.14786 | 1.02E-12 |
|  | CNN1 | 3.834825 | 3.15E-72 |  |  | KRT4 | -3.09284 | 4.75E-15 |
|  | ATP4A | 3.829305 | 3.95E-31 |  |  | REG3G | -3.01416 | 1.56E-15 |
|  | MYH11 | 3.82658 | 1.27E-66 |  |  | TMPRSS11B | -2.9845 | 1.07E-10 |
|  | HAND2-AS1 | 3.78063 | 5.94E-61 |  |  | KRTDAP | -2.98407 | 6.56E-18 |
|  | AC053503.5 | 3.72851 | 1.6E-40 |  |  | KRT13 | -2.89279 | 1.4E-14 |
|  | AC006007.1 | 3.690454 | 2.55E-14 |  |  | SPRR2C | -2.76766 | 0.00039 |
|  | LMO1 | 3.688032 | 6.5E-41 |  |  | LCE3E | -2.76647 | 6.57E-06 |
|  | SYNM | 3.667388 | 7.19E-64 |  |  | CTRB1 | -2.72653 | 3.05E-13 |
|  | MORN5 | 3.636493 | 9.26E-41 |  |  | KRT14 | -2.67849 | 1.46E-15 |
|  | HSPB6 | 3.600107 | 6.96E-64 |  |  | KRT78 | -2.65156 | 2.03E-18 |
|  | SYNPO2 | 3.597985 | 3.86E-67 |  |  | CPA1 | -2.6117 | 2.55E-14 |
|  | PGA3 | 3.559703 | 1.63E-14 |  |  | LCE3D | -2.58272 | 3.25E-06 |
|  | CARTPT | 3.524482 | 1.63E-25 |  |  | BX510359.5 | -2.58123 | 0.00052 |
|  | MLNR | 3.520583 | 1.14E-52 |  |  | S100A7 | -2.55421 | 1.87E-09 |
|  | AC053503.3 | 3.515696 | 3.34E-36 |  |  | S100A7A | -2.47066 | 9.5E-09 |
|  | FLNC | 3.512991 | 7.21E-72 |  |  | SPRR3 | -2.47053 | 7.51E-10 |
|  | ATP4B | 3.499929 | 1.02E-24 |  |  | IVL | -2.42942 | 7.88E-07 |
|  | ATP1A2 | 3.49548 | 3.6E-60 |  |  | SPRR2D | -2.26984 | 1.05E-08 |
|  | LDB3 | 3.486824 | 6.81E-78 |  |  | PAGE1 | -2.25229 | 1.84E-11 |
|  | TACR2 | 3.483184 | 1.99E-57 |  |  | SBSN | -2.24333 | 1.84E-11 |
|  | ACTN2 | 3.436404 | 1.93E-53 |  |  | KRT24 | -2.22498 | 1.14E-08 |
|  | PLIN4 | 3.424547 | 1.63E-59 |  |  | TMPRSS11D | -2.19589 | 2.85E-13 |
|  | HAND2 | 3.404781 | 1.66E-49 |  |  | TMPRSS11A | -2.16722 | 4.54E-08 |
|  |  |  |  |  |  |  |  |  |
| **UCEC** | DKK4 | 4.463754 | 2.86E-32 |  |  | SOX1-OT | -2.8321 | 4.86E-06 |
|  | CACNA1S | 3.932173 | 5.93E-40 |  |  | LINC02582 | -2.82896 | 5.66E-09 |
|  | DEFA5 | 3.819052 | 4.34E-09 |  |  | H3C14 | -2.64817 | 1.94E-06 |
|  | SCGB1D4 | 3.569165 | 6.85E-30 |  |  | CSN3 | -2.41695 | 9.94E-06 |
|  | LY6L | 3.461639 | 3.78E-12 |  |  | ASNSP1 | -2.22575 | 3E-09 |
|  | FGF3 | 3.192026 | 1.15E-16 |  |  | LINC02267 | -2.20854 | 2.16E-05 |
|  | DEFA6 | 3.180518 | 2.22E-07 |  |  | ESRG | -2.1719 | 1.67E-06 |
|  | MYH7B | 3.057163 | 8.33E-60 |  |  | COX7B2 | -2.16445 | 8.61E-05 |
|  | MT4 | 3.003322 | 7.89E-08 |  |  | CALCA | -2.09621 | 2.97E-10 |
|  | CST1 | 2.878676 | 4.13E-23 |  |  | MAGEC1 | -2.08749 | 5.33E-05 |
|  | DBH | 2.869849 | 1.16E-44 |  |  | SCARNA1 | -2.06171 | 2.5E-07 |
|  | FGF8 | 2.790128 | 5.53E-31 |  |  | LINC02241 | -2.04071 | 8.61E-07 |
|  | GP2 | 2.733085 | 1.3E-11 |  |  | AL359095.1 | -2.03164 | 3.19E-05 |
|  | PRR9 | 2.681432 | 3.15E-07 |  |  | CDH19 | -2.031 | 2.3E-06 |
|  | TPH1 | 2.670639 | 8.1E-32 |  |  | FAM9C | -2.01546 | 4.15E-08 |
|  | TRH | 2.651408 | 2.1E-17 |  |  | SLITRK2 | -2.00881 | 7.28E-11 |
|  | CALML3 | 2.629303 | 7.37E-16 |  |  | XAGE2 | -1.99633 | 1.76E-05 |
|  | LINC02178 | 2.621949 | 2.34E-19 |  |  | MAGEA9B | -1.9605 | 0.000222 |
|  | CST2 | 2.60473 | 1.55E-28 |  |  | CLDN16 | -1.94761 | 4.36E-15 |
|  | NOTUM | 2.528843 | 2.87E-23 |  |  | GPR12 | -1.92959 | 9.07E-06 |
|  | AC128707.1 | 2.521285 | 3.82E-07 |  |  | FOXD3 | -1.92514 | 6.71E-07 |
|  | KRTAP11-1 | 2.501929 | 7.89E-08 |  |  | PCDH15 | -1.91832 | 1.02E-11 |
|  | GPX2 | 2.480432 | 5.55E-23 |  |  | ZFP42 | -1.91321 | 1.45E-05 |
|  | RPL6P5 | 2.440681 | 4.02E-06 |  |  | NKX2-1-AS1 | -1.90357 | 0.003829 |
|  | WNT16 | 2.408017 | 1.01E-29 |  |  | AL391095.2 | -1.89971 | 0.000134 |
|  | ADAMTSL2 | 2.389168 | 8.33E-60 |  |  | CTAG2 | -1.88067 | 0.001075 |
|  | LY6G6C | 2.389021 | 1.15E-18 |  |  | SP8 | -1.87614 | 6.68E-09 |
|  | EDN3 | 2.377345 | 1.26E-18 |  |  | AF240627.1 | -1.8715 | 0.001089 |
|  | AC044893.1 | 2.36381 | 2.38E-07 |  |  | GUCA1C | -1.87142 | 0.000199 |
|  | DPEP1 | 2.360235 | 1.59E-28 |  |  | MT-TT | -1.86488 | 8.18E-15 |
