# Supplementary material for: Utility of G protein-coupled oestrogen receptor 1 as a biomarker for pan-cancer diagnosis, prognosis and immune infiltration: a comprehensive bioinformatics analysis
Source: Aging (Albany NY). 2023 Nov 2;15(21):12021–67. doi: 10.18632/aging.205162 (PMC10683611; doi:10.18632/aging.205162)
Supplement: Supplementary Table 4 [file aging-15-205162-s004.docx]

**Supplementary Table 4. The interaction proteins of top 30 DEGs of high- and low-GPER1 expression and their co-expression scores in different cancers.**

| **Cancer** | **node1** | **node2** | **coexpression** | **experimentally_determined_interaction** | **database_annotated** | **automated_textmining** | **combined_score** |
| --- | --- | --- | --- | --- | --- | --- | --- |
| BRCA | FGA | FGG | 0.993 | 0.986 | 0.9 | 0.961 | 0.999 |
|  | ORM1 | ORM2 | 0.954 | 0.68 | 0 | 0.91 | 0.984 |
|  | CHGA | CHGB | 0.407 | 0.788 | 0 | 0.875 | 0.982 |
|  | LALBA | CSN2 | 0.294 | 0 | 0 | 0.948 | 0.962 |
|  | CSN3 | CSN2 | 0.189 | 0.213 | 0 | 0.931 | 0.952 |
|  | LALBA | CSN3 | 0.201 | 0 | 0 | 0.911 | 0.926 |
|  | CARTPT | LEP | 0 | 0 | 0 | 0.911 | 0.911 |
|  | NTS | TRH | 0.054 | 0 | 0.5 | 0.811 | 0.903 |
|  | UCP1 | LEP | 0.062 | 0 | 0 | 0.828 | 0.832 |
|  | TRH | LEP | 0 | 0 | 0 | 0.829 | 0.83 |
|  | CARTPT | TRH | 0.062 | 0 | 0 | 0.747 | 0.753 |
|  | FGA | ORM2 | 0.583 | 0 | 0 | 0.362 | 0.723 |
|  | ORM1 | FGA | 0.488 | 0 | 0 | 0.465 | 0.714 |
|  | ORM1 | FGG | 0.606 | 0 | 0 | 0.297 | 0.711 |
|  | FGG | ORM2 | 0.607 | 0 | 0 | 0.294 | 0.711 |
|  | CHGA | NEUROD1 | 0.377 | 0 | 0 | 0.551 | 0.708 |
|  | MAGEA10 | MAGEA4 | 0.544 | 0.295 | 0 | 0.805 | 0.679 |
|  | CARTPT | PCSK1 | 0.111 | 0 | 0 | 0.573 | 0.604 |
|  | CHGA | PCSK1 | 0.196 | 0 | 0 | 0.505 | 0.585 |
|  | PCSK1 | CHGB | 0.188 | 0 | 0 | 0.509 | 0.585 |
|  | NEUROD1 | CPLX2 | 0.208 | 0 | 0 | 0.497 | 0.584 |
|  | CHGA | NTS | 0.062 | 0 | 0 | 0.559 | 0.569 |
|  | NTS | CARTPT | 0.062 | 0 | 0 | 0.56 | 0.569 |
|  | NEUROD1 | PCSK1 | 0.097 | 0 | 0 | 0.531 | 0.558 |
|  | NTS | LEP | 0 | 0 | 0 | 0.539 | 0.539 |
|  | NEUROD1 | FABP7 | 0.096 | 0 | 0 | 0.508 | 0.536 |
|  | MYF6 | MYL1 | 0.314 | 0 | 0 | 0.341 | 0.528 |
|  | PCSK1 | LEP | 0 | 0 | 0 | 0.521 | 0.521 |
|  | LBP | ORM1 | 0.138 | 0 | 0 | 0.439 | 0.495 |
|  | CHGA | CPLX2 | 0.415 | 0 | 0 | 0.168 | 0.493 |
|  | CPLX2 | CHGB | 0.375 | 0 | 0 | 0.194 | 0.476 |
|  | CARTPT | CHGB | 0.233 | 0 | 0 | 0.323 | 0.459 |
|  | CHGA | CARTPT | 0.309 | 0 | 0 | 0.219 | 0.438 |
|  | NEUROD1 | CHGB | 0.273 | 0 | 0 | 0.256 | 0.436 |
|  | LBP | ORM2 | 0.102 | 0 | 0 | 0.396 | 0.434 |
|  | UCP1 | TRH | 0 | 0 | 0 | 0.429 | 0.429 |
|  | CLVS2 | CPLX2 | 0.425 | 0 | 0 | 0 | 0.425 |
|  | UCP1 | CARTPT | 0 | 0 | 0 | 0.422 | 0.422 |
|  | NTS | CHGB | 0.062 | 0 | 0 | 0.4 | 0.413 |
|  | CHGB | XKR7 | 0.226 | 0 | 0 | 0.267 | 0.409 |
|  |  |  |  |  |  |  |  |
| DLCA | CTAG2 | MAGEA4 | 0.098 | 0 | 0 | 0.71 | 0.727 |
|  | CT45A1 | CT45A10 | 0 | 0.309 | 0.54 | 0.562 | 0.671 |
|  | MAGEA4 | CT45A1 | 0.139 | 0 | 0 | 0.558 | 0.603 |
|  | CT45A1 | CT45A3 | 0 | 0 | 0.54 | 0.829 | 0.545 |
|  | CT45A3 | CT45A10 | 0 | 0 | 0.54 | 0 | 0.54 |
|  | CTAG2 | CT45A1 | 0.07 | 0 | 0 | 0.506 | 0.52 |
|  | PLCZ1 | SPERT | 0.509 | 0 | 0 | 0 | 0.509 |
|  | CTAG2 | MAGEB1 | 0.063 | 0 | 0 | 0.458 | 0.47 |
|  |  |  |  |  |  |  |  |
| ESCA | ATP4A | ATP4B | 0.062 | 0.344 | 0.8 | 0.833 | 0.976 |
|  | PGA5 | PGA3 | 0.071 | 0 | 0.8 | 0.902 | 0.808 |
|  | GH2 | GHRH | 0 | 0 | 0 | 0.749 | 0.749 |
|  | ATP4B | PGC | 0.049 | 0 | 0 | 0.673 | 0.676 |
|  | UGT1A8 | HSD3B2 | 0 | 0.056 | 0.65 | 0.072 | 0.666 |
|  | ACHE | NRXN1 | 0.092 | 0.177 | 0 | 0.532 | 0.62 |
|  | GKN1 | LIPF | 0.095 | 0 | 0 | 0.556 | 0.581 |
|  | PGA3 | LIPF | 0.062 | 0.076 | 0 | 0.519 | 0.546 |
|  | PGC | GKN1 | 0.065 | 0 | 0 | 0.495 | 0.507 |
|  | ASTN1 | BRINP3 | 0.17 | 0 | 0 | 0.409 | 0.489 |
|  | GRIK3 | NRXN1 | 0.231 | 0.104 | 0 | 0.295 | 0.472 |
|  | ATP4B | GKN1 | 0 | 0 | 0 | 0.466 | 0.466 |
|  | GABRA5 | NRXN1 | 0.184 | 0.079 | 0 | 0.346 | 0.465 |
|  | ASTN1 | NRXN1 | 0.352 | 0 | 0 | 0.197 | 0.457 |
|  | PGA5 | GKN1 | 0.064 | 0 | 0 | 0.441 | 0.454 |
|  | PGA5 | LIPF | 0.062 | 0.076 | 0 | 0.39 | 0.425 |
|  | PGC | LIPF | 0.063 | 0.076 | 0 | 0.384 | 0.42 |
|  | ATP4A | PGC | 0.062 | 0.117 | 0 | 0.354 | 0.418 |
|  |  |  |  |  |  |  |  |
| HNSC | LCE3B | LCE2D | 0.089 | 0 | 0.9 | 0.294 | 0.93 |
|  | DEFB4A | DEFB103A | 0.143 | 0 | 0.8 | 0.913 | 0.841 |
|  | SPINK7 | SPINK6 | 0 | 0 | 0 | 0.736 | 0.736 |
|  | CRNN | KPRP | 0.186 | 0 | 0 | 0.634 | 0.689 |
|  | CLDN17 | CLDN19 | 0.062 | 0 | 0.6 | 0.545 | 0.631 |
|  | HRNR | KPRP | 0.093 | 0 | 0 | 0.594 | 0.616 |
|  | FLG2 | KPRP | 0.115 | 0 | 0 | 0.572 | 0.605 |
|  | CRNN | TGM3 | 0.099 | 0 | 0 | 0.556 | 0.582 |
|  | SERPINB12 | KPRP | 0.233 | 0 | 0 | 0.459 | 0.567 |
|  | LCE3B | FLG2 | 0.062 | 0 | 0 | 0.552 | 0.562 |
|  | TGM3 | FLG2 | 0.062 | 0 | 0 | 0.528 | 0.538 |
|  | KRT37 | KRT24 | 0.06 | 0 | 0.5 | 0.155 | 0.518 |
|  | LCE3B | HRNR | 0.062 | 0 | 0 | 0.476 | 0.488 |
|  | KRT2 | TGM3 | 0.092 | 0.179 | 0 | 0.349 | 0.472 |
|  | HRNR | TGM3 | 0.062 | 0 | 0 | 0.46 | 0.471 |
|  | KRT2 | FLG2 | 0.062 | 0 | 0 | 0.445 | 0.457 |
|  | KRT2 | HRNR | 0.062 | 0 | 0 | 0.435 | 0.448 |
|  | KRT2 | LCE2D | 0.138 | 0 | 0 | 0.37 | 0.433 |
|  | KRT2 | KPRP | 0.157 | 0 | 0 | 0.352 | 0.431 |
|  | CRNN | LCE3B | 0 | 0 | 0 | 0.407 | 0.407 |
|  |  |  |  |  |  |  |  |
| KIRC | REN | KLK4 | 0 | 0 | 0 | 0.956 | 0.956 |
|  | G6PC | ALDOB | 0.145 | 0 | 0.8 | 0.483 | 0.904 |
|  | FDCSP | ODAM | 0.066 | 0 | 0 | 0.71 | 0.717 |
|  | REN | PLG | 0.065 | 0 | 0 | 0.642 | 0.652 |
|  | KLK4 | ODAM | 0 | 0 | 0 | 0.635 | 0.635 |
|  | REN | KLK1 | 0 | 0 | 0 | 0.61 | 0.61 |
|  | SLC34A1 | UMOD | 0.174 | 0 | 0 | 0.522 | 0.589 |
|  | RHCG | SLC34A1 | 0.522 | 0 | 0 | 0.15 | 0.576 |
|  | TRIM63 | MYH8 | 0.089 | 0.182 | 0 | 0.471 | 0.572 |
|  | REN | UMOD | 0.085 | 0 | 0 | 0.51 | 0.532 |
|  | REN | SLC6A19 | 0.051 | 0 | 0 | 0.425 | 0.431 |
|  | KLK1 | UMOD | 0 | 0 | 0 | 0.422 | 0.422 |
|  |  |  |  |  |  |  |  |
| KIRP | CRP | HP | 0.09 | 0 | 0 | 0.858 | 0.865 |
|  | MAGEC1 | MAGEC2 | 0.779 | 0 | 0 | 0.831 | 0.79 |
|  | PSCA | KLK3 | 0 | 0 | 0 | 0.681 | 0.681 |
|  | KLK3 | PAGE1 | 0 | 0 | 0 | 0.665 | 0.665 |
|  | ZIC5 | ZIC2 | 0.614 | 0 | 0 | 0.773 | 0.649 |
|  | CRP | KLK3 | 0 | 0 | 0 | 0.602 | 0.602 |
|  | SCNN1G | TMPRSS4 | 0.062 | 0 | 0 | 0.534 | 0.544 |
|  | SOSTDC1 | CASP14 | 0 | 0 | 0 | 0.53 | 0.53 |
|  | MAGEC2 | MAGEA3 | 0.452 | 0 | 0 | 0.737 | 0.502 |
|  | MAGEC1 | MAGEA3 | 0.419 | 0 | 0 | 0.76 | 0.486 |
|  | PRDM14 | CASP14 | 0 | 0.056 | 0 | 0.432 | 0.44 |
|  |  |  |  |  |  |  |  |
| LUAD | NTS | SST | 0.065 | 0 | 0 | 0.873 | 0.876 |
|  | SST | CALCA | 0.066 | 0 | 0 | 0.866 | 0.87 |
|  | NTS | CALCA | 0 | 0 | 0 | 0.773 | 0.773 |
|  | MAGEA4 | GAGE2A | 0.152 | 0 | 0 | 0.519 | 0.574 |
|  | BPIFA1 | KLK13 | 0 | 0 | 0 | 0.572 | 0.572 |
|  | CALCA | CGA | 0 | 0 | 0.5 | 0.094 | 0.527 |
|  | BPIFA1 | KLK14 | 0 | 0 | 0 | 0.42 | 0.42 |
|  |  |  |  |  |  |  |  |
| PAAD | CLPS | PNLIP | 0.351 | 0.612 | 0.8 | 0.996 | 0.999 |
|  | CPA1 | CELA3B | 0.829 | 0.569 | 0 | 0.696 | 0.975 |
|  | PNLIP | CEL | 0.389 | 0 | 0.8 | 0.794 | 0.972 |
|  | CPA1 | CELA3A | 0.759 | 0.569 | 0 | 0.689 | 0.964 |
|  | CPB1 | CTRL | 0.921 | 0.139 | 0 | 0.478 | 0.961 |
|  | CPA1 | CTRC | 0.431 | 0.702 | 0 | 0.77 | 0.957 |
|  | PRSS1 | CTRC | 0.228 | 0 | 0.9 | 0.878 | 0.941 |
|  | CELA2A | CPB1 | 0.824 | 0.194 | 0 | 0.617 | 0.941 |
|  | CPA1 | CTRL | 0.902 | 0.139 | 0 | 0.344 | 0.939 |
|  | CPA1 | CTRB2 | 0.804 | 0.139 | 0 | 0.629 | 0.932 |
|  | CPA1 | CEL | 0.863 | 0 | 0 | 0.509 | 0.929 |
|  | CEL | CTRL | 0.904 | 0.056 | 0 | 0.165 | 0.917 |
|  | CELA3B | CPB1 | 0.755 | 0.262 | 0 | 0.567 | 0.915 |
|  | CELA3B | CEL | 0.825 | 0.056 | 0 | 0.38 | 0.888 |
|  | PNLIP | CPB1 | 0.784 | 0 | 0 | 0.494 | 0.886 |
|  | CELA3A | CPB1 | 0.636 | 0.262 | 0 | 0.602 | 0.884 |
|  | CTRC | CPB1 | 0.66 | 0.344 | 0 | 0.484 | 0.875 |
|  | PLA2G1B | CPB1 | 0.81 | 0 | 0 | 0.359 | 0.873 |
|  | CEL | CPB1 | 0.809 | 0 | 0 | 0.364 | 0.873 |
|  | CPA1 | CPA2 | 0.332 | 0 | 0.8 | 0.924 | 0.866 |
|  | CLPS | CEL | 0.502 | 0 | 0 | 0.736 | 0.863 |
|  | CELA3B | CTRL | 0.846 | 0 | 0 | 0.421 | 0.859 |
|  | CPA1 | CELA2A | 0.529 | 0.194 | 0 | 0.655 | 0.858 |
|  | CPA1 | CPB1 | 0.826 | 0 | 0 | 0.773 | 0.843 |
|  | CELA2A | CTRL | 0.823 | 0 | 0 | 0.475 | 0.839 |
|  | CPA2 | CELA3A | 0.268 | 0.345 | 0 | 0.66 | 0.823 |
|  | SYCN | CELA3B | 0.665 | 0 | 0 | 0.487 | 0.821 |
|  | CPA1 | PRSS1 | 0.241 | 0.139 | 0 | 0.738 | 0.814 |
|  | PAX7 | PAX3 | 0.076 | 0 | 0.8 | 0.906 | 0.811 |
|  | AMY1B | AMY2A | 0 | 0 | 0.8 | 0.857 | 0.803 |
|  | CPA1 | PNLIP | 0.502 | 0 | 0 | 0.595 | 0.79 |
|  | CTRB2 | CEL | 0.66 | 0.056 | 0 | 0.383 | 0.785 |
|  | CELA3A | CEL | 0.666 | 0.056 | 0 | 0.359 | 0.78 |
|  | CELA3A | CTRL | 0.755 | 0 | 0 | 0.409 | 0.779 |
|  | CELA3B | PNLIP | 0.433 | 0.057 | 0 | 0.621 | 0.779 |
|  | CLPS | CPB1 | 0.66 | 0 | 0 | 0.359 | 0.773 |
|  | CELA3A | SYCN | 0.651 | 0 | 0 | 0.352 | 0.764 |
|  | CELA3B | CTRC | 0.437 | 0.569 | 0 | 0.855 | 0.763 |
|  | CTRB2 | CTRL | 0.751 | 0 | 0 | 0.345 | 0.759 |
|  | CLPS | CELA3B | 0.662 | 0 | 0 | 0.311 | 0.758 |
|  | CTRB2 | CPB1 | 0.491 | 0.139 | 0 | 0.484 | 0.754 |
|  | CELA3A | PNLIP | 0.46 | 0.057 | 0 | 0.547 | 0.749 |
|  | CPA1 | SYCN | 0.478 | 0 | 0 | 0.531 | 0.744 |
|  | CPA2 | CELA2A | 0.207 | 0.194 | 0 | 0.631 | 0.743 |
|  | CPA2 | CTRC | 0.146 | 0.369 | 0 | 0.558 | 0.741 |
|  | CLPS | CELA3A | 0.579 | 0 | 0 | 0.402 | 0.738 |
|  | PNLIP | AMY2A | 0.156 | 0 | 0 | 0.702 | 0.738 |
|  | CELA2A | CEL | 0.52 | 0.056 | 0 | 0.452 | 0.73 |
|  | CPA2 | CELA3B | 0.144 | 0.345 | 0 | 0.538 | 0.718 |
|  | CTRB2 | PRSS1 | 0.183 | 0.265 | 0.5 | 0.607 | 0.714 |
|  | PLA2G1B | PNLIP | 0.382 | 0 | 0 | 0.556 | 0.714 |
|  | CEL | CTRC | 0.479 | 0.056 | 0 | 0.46 | 0.711 |
|  | PRSS1 | CPB1 | 0.344 | 0.139 | 0 | 0.528 | 0.71 |
|  | CELA3A | CTRC | 0.294 | 0.569 | 0 | 0.872 | 0.705 |
|  | CPA2 | CTRB2 | 0.222 | 0.139 | 0 | 0.592 | 0.703 |
|  | SYCN | PNLIP | 0.457 | 0 | 0 | 0.43 | 0.677 |
|  | PRSS1 | PNLIP | 0.199 | 0.057 | 0 | 0.591 | 0.664 |
|  | CPA1 | AMY2A | 0.155 | 0.059 | 0 | 0.606 | 0.659 |
|  | CTRB2 | PNLIP | 0.508 | 0.057 | 0 | 0.322 | 0.659 |
|  | CPA1 | CLPS | 0.427 | 0 | 0 | 0.426 | 0.657 |
|  | CELA2A | PNLIP | 0.216 | 0.057 | 0 | 0.548 | 0.637 |
|  | CPA1 | RBPJL | 0.065 | 0 | 0 | 0.623 | 0.633 |
|  | SYCN | CPB1 | 0.381 | 0 | 0 | 0.414 | 0.622 |
|  | CPB1 | SERPINI2 | 0.082 | 0 | 0 | 0.606 | 0.622 |
|  | CPA2 | PRSS1 | 0.187 | 0.139 | 0 | 0.499 | 0.619 |
|  | CPB1 | AMY2A | 0.187 | 0.059 | 0 | 0.44 | 0.612 |
|  | PNLIP | CTRC | 0.16 | 0.057 | 0 | 0.539 | 0.603 |
|  | CPA2 | PNLIP | 0.268 | 0 | 0 | 0.467 | 0.594 |
|  | CPA1 | PLA2G1B | 0.38 | 0 | 0 | 0.328 | 0.566 |
|  | CELA2A | CUZD1 | 0.066 | 0.126 | 0 | 0.511 | 0.565 |
|  | PLA2G1B | CEL | 0.249 | 0 | 0 | 0.443 | 0.564 |
|  | CPA2 | RBPJL | 0 | 0 | 0 | 0.559 | 0.559 |
|  | SYCN | CUZD1 | 0.108 | 0 | 0 | 0.524 | 0.557 |
|  | CELA2A | AMY2A | 0.157 | 0.058 | 0 | 0.475 | 0.547 |
|  | CLPS | SYCN | 0.377 | 0 | 0 | 0.293 | 0.541 |
|  | CTRC | CTRL | 0.488 | 0 | 0 | 0.428 | 0.541 |
|  | CELA3A | CTRB2 | 0.468 | 0 | 0 | 0.669 | 0.537 |
|  | CLPS | PLA2G1B | 0.18 | 0 | 0 | 0.454 | 0.533 |
|  | CELA3A | CUZD1 | 0.141 | 0.126 | 0 | 0.425 | 0.531 |
|  | PRSS1 | CEL | 0.203 | 0.056 | 0 | 0.425 | 0.529 |
|  | PLA2G1B | SYCN | 0.334 | 0 | 0 | 0.314 | 0.524 |
|  | VPREB3 | TCL1A | 0.323 | 0 | 0 | 0.32 | 0.519 |
|  | CTRB2 | CELA3B | 0.47 | 0 | 0 | 0.404 | 0.518 |
|  | CLPS | CELA2A | 0.193 | 0 | 0 | 0.422 | 0.514 |
|  | PLA2G1B | CELA3B | 0.149 | 0 | 0 | 0.438 | 0.502 |
|  | CEL | AMY2A | 0.122 | 0 | 0 | 0.453 | 0.499 |
|  | CPA2 | CTRL | 0.238 | 0.139 | 0 | 0.294 | 0.497 |
|  | CELA3A | PRSS1 | 0.388 | 0 | 0 | 0.637 | 0.496 |
|  | CELA3A | AMY2A | 0.119 | 0.058 | 0 | 0.442 | 0.496 |
|  | CTRB2 | SYCN | 0.347 | 0 | 0 | 0.251 | 0.491 |
|  | CELA3A | PLA2G1B | 0.267 | 0 | 0 | 0.32 | 0.481 |
|  | SYCN | AMY2A | 0.108 | 0 | 0 | 0.442 | 0.481 |
|  | CLPS | AMY2A | 0.096 | 0 | 0 | 0.447 | 0.479 |
|  | CELA2A | CTRC | 0.455 | 0 | 0 | 0.769 | 0.479 |
|  | CELA3B | AMY2A | 0.084 | 0.058 | 0 | 0.433 | 0.468 |
|  | SYCN | CELA2A | 0.175 | 0 | 0 | 0.374 | 0.462 |
|  | CPA2 | AMY2A | 0.123 | 0.059 | 0 | 0.396 | 0.458 |
|  | CPA2 | SYCN | 0.224 | 0 | 0 | 0.322 | 0.452 |
|  | SYCN | CTRC | 0.17 | 0 | 0 | 0.367 | 0.452 |
|  | CPA2 | CEL | 0.215 | 0 | 0 | 0.322 | 0.446 |
|  | CTRB2 | CELA2A | 0.361 | 0 | 0 | 0.635 | 0.443 |
|  | PRSS1 | AMY2A | 0.108 | 0.058 | 0 | 0.384 | 0.437 |
|  | CLPS | CTRB2 | 0.311 | 0 | 0 | 0.214 | 0.435 |
|  | CPA2 | CPB1 | 0.361 | 0 | 0 | 0.823 | 0.431 |
|  | SYCN | CTRL | 0.356 | 0 | 0 | 0.134 | 0.419 |
|  | SYCN | CEL | 0.293 | 0 | 0 | 0.21 | 0.418 |
|  | CTRC | AMY2A | 0.133 | 0.058 | 0 | 0.33 | 0.405 |
|  |  |  |  |  |  |  |  |
| SARC | SFTPB | SFTPA1 | 0.427 | 0 | 0.6 | 0.892 | 0.973 |
|  | GRIK1 | CACNG5 | 0.063 | 0.212 | 0.54 | 0.078 | 0.644 |
|  | SHH | LHX3 | 0 | 0 | 0 | 0.589 | 0.589 |
|  | MAGEA4 | SPANXD | 0.07 | 0 | 0 | 0.528 | 0.542 |
|  | FGF19 | SHH | 0 | 0 | 0 | 0.54 | 0.54 |
|  | DLK1 | ADIPOQ | 0.052 | 0 | 0 | 0.497 | 0.502 |
|  | SPANXD | PRR32 | 0 | 0 | 0 | 0.469 | 0.469 |
|  | CBLN1 | ELAVL3 | 0.346 | 0 | 0 | 0.127 | 0.405 |
|  |  |  |  |  |  |  |  |
| STAD | LDB3 | ACTN2 | 0.543 | 0.839 | 0 | 0.978 | 0.998 |
|  | SPRR2E | SPRR2D | 0.521 | 0 | 0.9 | 0.681 | 0.983 |
|  | ATP4A | ATP4B | 0.062 | 0.344 | 0.8 | 0.833 | 0.976 |
|  | SPRR2E | SPRR2B | 0.483 | 0 | 0.9 | 0.519 | 0.972 |
|  | SPRR3 | SPRR2D | 0.332 | 0 | 0.9 | 0.561 | 0.968 |
|  | LCE3D | LCE3E | 0.678 | 0 | 0.9 | 0.909 | 0.967 |
|  | SPRR3 | SPRR2B | 0.1 | 0 | 0.9 | 0.635 | 0.964 |
|  | SPRR3 | SPRR2E | 0.3 | 0 | 0.9 | 0.508 | 0.962 |
|  | SPRR2B | SPRR2D | 0.292 | 0 | 0.9 | 0.519 | 0.962 |
|  | PGA3 | PGA4 | 0.089 | 0.8 | 0.8 | 0.837 | 0.96 |
|  | MYH11 | ACTG2 | 0.566 | 0.369 | 0.6 | 0.641 | 0.955 |
|  | CPA1 | CTRB1 | 0.817 | 0.139 | 0 | 0.713 | 0.951 |
|  | SPRR3 | IVL | 0.283 | 0.213 | 0.6 | 0.727 | 0.93 |
|  | SPRR2B | LCE3E | 0.52 | 0 | 0.6 | 0.636 | 0.924 |
|  | CNN1 | ACTG2 | 0.789 | 0.179 | 0 | 0.588 | 0.922 |
|  | CNN1 | MYH11 | 0.572 | 0 | 0 | 0.793 | 0.908 |
|  | SPRR2B | LCE3D | 0.371 | 0 | 0.6 | 0.655 | 0.905 |
|  | SPRR2E | LCE3E | 0.489 | 0 | 0.6 | 0.468 | 0.881 |
|  | S100A7 | S100A7A | 0.393 | 0 | 0.8 | 0.681 | 0.875 |
|  | SPRR2E | LCE3D | 0.461 | 0 | 0.6 | 0.468 | 0.875 |
|  | PGA5 | PGA4 | 0.334 | 0 | 0.8 | 0.766 | 0.862 |
|  | SPRR3 | LCE3D | 0.15 | 0 | 0.6 | 0.6 | 0.852 |
|  | IVL | LCE3D | 0.262 | 0 | 0.6 | 0.484 | 0.834 |
|  | FLNC | LDB3 | 0.065 | 0.149 | 0 | 0.807 | 0.833 |
|  | SPRR2D | LCE3D | 0.305 | 0 | 0.6 | 0.402 | 0.819 |
|  | PGA5 | PGA3 | 0.071 | 0 | 0.8 | 0.902 | 0.808 |
|  | DES | ACTN2 | 0.176 | 0.057 | 0.6 | 0.425 | 0.797 |
|  | SPRR2E | IVL | 0.333 | 0 | 0.6 | 0.294 | 0.795 |
|  | CTRB1 | PNLIP | 0.533 | 0.057 | 0 | 0.568 | 0.793 |
|  | SPRR2D | IVL | 0.299 | 0 | 0.6 | 0.322 | 0.793 |
|  | CPA1 | PNLIP | 0.502 | 0 | 0 | 0.595 | 0.79 |
|  | SPRR3 | LCE3E | 0.16 | 0 | 0.6 | 0.392 | 0.778 |
|  | S100A7 | IVL | 0.243 | 0 | 0 | 0.719 | 0.778 |
|  | SPRR2D | LCE3E | 0.294 | 0 | 0.6 | 0.275 | 0.777 |
|  | SPRR2B | IVL | 0.135 | 0 | 0.6 | 0.39 | 0.77 |
|  | IVL | LCE3E | 0.213 | 0 | 0.6 | 0.327 | 0.769 |
|  | KRT14 | IVL | 0.197 | 0 | 0 | 0.71 | 0.757 |
|  | ATP4B | ATP1A2 | 0.062 | 0.288 | 0.54 | 0.303 | 0.757 |
|  | SYNPO2 | FLNC | 0.081 | 0.352 | 0 | 0.6 | 0.741 |
|  | KRT13 | KRT4 | 0.42 | 0.446 | 0 | 0.816 | 0.728 |
|  | KRTDAP | SBSN | 0.163 | 0 | 0 | 0.683 | 0.723 |
|  | CRNN | SPRR3 | 0.379 | 0 | 0 | 0.56 | 0.715 |
|  | KRT13 | IVL | 0.138 | 0 | 0 | 0.656 | 0.69 |
|  | HAND2 | SMYD1 | 0.084 | 0.056 | 0 | 0.646 | 0.668 |
|  | SPRR3 | KRT4 | 0.398 | 0 | 0 | 0.391 | 0.618 |
|  | IVL | KRT4 | 0.152 | 0 | 0 | 0.53 | 0.584 |
|  | ACTG2 | ACTN2 | 0.087 | 0.259 | 0 | 0.427 | 0.578 |
|  | SMYD1 | LDB3 | 0.556 | 0.058 | 0 | 0.071 | 0.578 |
|  | S100A7A | IVL | 0.123 | 0 | 0 | 0.533 | 0.573 |
|  | CNN1 | LDB3 | 0.063 | 0.067 | 0 | 0.545 | 0.567 |
|  | KRT14 | KRTDAP | 0.153 | 0 | 0 | 0.509 | 0.566 |
|  | KRTDAP | LCE3D | 0.286 | 0 | 0 | 0.413 | 0.563 |
|  | CRNN | IVL | 0.139 | 0 | 0 | 0.506 | 0.556 |
|  | SYNPO2 | ACTN2 | 0.096 | 0.375 | 0 | 0.273 | 0.554 |
|  | S100A7 | LCE3D | 0.214 | 0 | 0 | 0.455 | 0.553 |
|  | DES | LDB3 | 0.192 | 0.101 | 0 | 0.431 | 0.55 |
|  | S100A7A | LCE3D | 0.139 | 0 | 0 | 0.492 | 0.543 |
|  | CRNN | KRT4 | 0.168 | 0 | 0 | 0.468 | 0.539 |
|  | KRT78 | SPRR2E | 0.333 | 0 | 0 | 0.323 | 0.529 |
|  | ATP4B | PGA4 | 0.049 | 0 | 0 | 0.518 | 0.522 |
|  | KRT14 | SBSN | 0.153 | 0 | 0 | 0.457 | 0.521 |
|  | SMYD1 | ACTN2 | 0.353 | 0.075 | 0 | 0.263 | 0.521 |
|  | KRTDAP | SPRR2E | 0.334 | 0 | 0 | 0.31 | 0.52 |
|  | KRTDAP | KRT4 | 0.157 | 0 | 0 | 0.452 | 0.518 |
|  | SPRR3 | S100A7 | 0.195 | 0 | 0 | 0.42 | 0.513 |
|  | S100A7 | SPRR2E | 0.344 | 0 | 0 | 0.287 | 0.512 |
|  | MYH11 | ACTN2 | 0.166 | 0.143 | 0 | 0.361 | 0.503 |
|  | FLNC | SYNM | 0.142 | 0.192 | 0 | 0.325 | 0.491 |
|  | S100A7 | SPRR2B | 0.197 | 0 | 0 | 0.391 | 0.49 |
|  | MYH11 | LDB3 | 0.14 | 0.149 | 0 | 0.354 | 0.487 |
|  | CRNN | LCE3D | 0.152 | 0 | 0 | 0.418 | 0.485 |
|  | KRT14 | KRT6C | 0.367 | 0.139 | 0 | 0.373 | 0.481 |
|  | SPRR2E | SBSN | 0.183 | 0 | 0 | 0.391 | 0.481 |
|  | SPRR3 | KRTDAP | 0.163 | 0 | 0 | 0.391 | 0.469 |
|  | KRT6C | KRT78 | 0.251 | 0 | 0.3 | 0.468 | 0.468 |
|  | IL36A | S100A7A | 0.065 | 0 | 0 | 0.455 | 0.468 |
|  | KRT78 | KRTDAP | 0.151 | 0 | 0 | 0.398 | 0.467 |
|  | FLNC | MYH11 | 0.065 | 0.108 | 0 | 0.409 | 0.464 |
|  | S100A7A | LCE3E | 0.152 | 0 | 0 | 0.392 | 0.462 |
|  | KRTDAP | IVL | 0.152 | 0 | 0 | 0.39 | 0.46 |
|  | ATP1A2 | KCNA1 | 0.291 | 0.056 | 0 | 0.251 | 0.455 |
|  | CRNN | SPRR2E | 0.274 | 0 | 0 | 0.255 | 0.436 |
|  | LCE3D | SBSN | 0.173 | 0 | 0 | 0.345 | 0.435 |
|  | KRT78 | KRT4 | 0.201 | 0 | 0.3 | 0.508 | 0.433 |
|  | SYNPO2 | MYH11 | 0.161 | 0.149 | 0 | 0.264 | 0.429 |
|  | S100A7A | SPRR2B | 0.177 | 0 | 0 | 0.335 | 0.429 |
|  | CRNN | ACTN2 | 0 | 0 | 0 | 0.426 | 0.426 |
|  | KRT14 | S100A7 | 0.177 | 0 | 0 | 0.322 | 0.418 |
|  | KRT13 | SPRR3 | 0.317 | 0 | 0 | 0.183 | 0.418 |
|  | CRNN | KRT78 | 0.284 | 0 | 0 | 0.22 | 0.417 |
|  | IVL | SBSN | 0.152 | 0 | 0 | 0.341 | 0.417 |
|  | KRT6C | KRTDAP | 0.23 | 0 | 0 | 0.27 | 0.414 |
|  | KRT14 | KRT13 | 0.171 | 0 | 0.3 | 0.758 | 0.412 |
|  | HAND2 | ACTN2 | 0.059 | 0 | 0 | 0.4 | 0.411 |
|  |  |  |  |  |  |  |  |
| UCEC | DEFA6 | DEFA5 | 0.798 | 0 | 0.6 | 0.857 | 0.922 |
|  | WNT16 | NOTUM | 0 | 0.141 | 0.6 | 0.468 | 0.801 |
|  | TPH1 | DBH | 0.111 | 0 | 0 | 0.773 | 0.789 |
|  | CALCA | DBH | 0 | 0 | 0 | 0.747 | 0.747 |
|  | DKK4 | WNT16 | 0 | 0.058 | 0.6 | 0.346 | 0.732 |
|  | CTAG2 | MAGEC1 | 0.094 | 0 | 0 | 0.704 | 0.72 |
|  | FGF8 | FGF3 | 0.063 | 0 | 0.5 | 0.93 | 0.68 |
|  | TRH | CALCA | 0.062 | 0 | 0 | 0.629 | 0.637 |
|  | LY6G6C | LY6L | 0 | 0 | 0 | 0.625 | 0.625 |
|  | ENSG00000123584 | CTAG2 | 0 | 0 | 0 | 0.595 | 0.595 |
|  | FGF8 | SP8 | 0 | 0.056 | 0 | 0.563 | 0.57 |
|  | ZFP42 | FOXD3 | 0.063 | 0.078 | 0 | 0.521 | 0.55 |
|  | TRH | EDN3 | 0 | 0 | 0.5 | 0.074 | 0.517 |
|  | FGF8 | FOXD3 | 0 | 0 | 0 | 0.455 | 0.455 |
|  | CST1 | CST2 | 0.433 | 0 | 0 | 0.909 | 0.443 |
|  | MT4 | GPX2 | 0.143 | 0 | 0 | 0.373 | 0.439 |
