# Supplementary material for: Utility of G protein-coupled oestrogen receptor 1 as a biomarker for pan-cancer diagnosis, prognosis and immune infiltration: a comprehensive bioinformatics analysis
Source: Aging (Albany NY). 2023 Nov 2;15(21):12021–67. doi: 10.18632/aging.205162 (PMC10683611; doi:10.18632/aging.205162)
Supplement: Supplementary Table 6 [file aging-15-205162-s006.docx]

**Supplementary Table 6. GO and KEGG enrichment analysis for top 30 DEGs of high- and low-GPER1 expression.**

| **CANCER** | **ONTOLOGY** | **ID** | **Description** | **GeneRatio** | **BgRatio** | **pvalue** | **p.adjust** | **qvalue** | **geneID** | **Count** |
| --- | --- | --- | --- | --- | --- | --- | --- | --- | --- | --- |
| BRCA | BP | GO:0090276 | regulation of peptide hormone secretion | 7/43 | 208/18670 | 4.4161E-07 | 0.00043072 | 0.00031894 | CHGA/FGA/FGG/LEP/NEUROD1/TRH/CARTPT | 7 |
|  | BP | GO:0050708 | regulation of protein secretion | 9/43 | 472/18670 | 1.0269E-06 | 0.00043072 | 0.00031894 | CHGA/FGA/FGG/LEP/NEUROD1/ORM1/ORM2/TRH/CARTPT | 9 |
|  | BP | GO:0030072 | peptide hormone secretion | 7/43 | 250/18670 | 1.5161E-06 | 0.00043072 | 0.00031894 | CHGA/FGA/FGG/LEP/NEUROD1/TRH/CARTPT | 7 |
|  | BP | GO:0002791 | regulation of peptide secretion | 9/43 | 500/18670 | 1.6534E-06 | 0.00043072 | 0.00031894 | CHGA/FGA/FGG/LEP/NEUROD1/ORM1/ORM2/TRH/CARTPT | 9 |
|  | BP | GO:0046883 | regulation of hormone secretion | 7/43 | 266/18670 | 2.2896E-06 | 0.00047716 | 0.00035332 | CHGA/FGA/FGG/LEP/NEUROD1/TRH/CARTPT | 7 |
|  | BP | GO:0046879 | hormone secretion | 7/43 | 312/18670 | 6.5445E-06 | 0.00113656 | 0.0008416 | CHGA/FGA/FGG/LEP/NEUROD1/TRH/CARTPT | 7 |
|  | BP | GO:0009914 | hormone transport | 7/43 | 322/18670 | 8.0413E-06 | 0.0011679 | 0.00086481 | CHGA/FGA/FGG/LEP/NEUROD1/TRH/CARTPT | 7 |
|  | BP | GO:0023061 | signal release | 8/43 | 462/18670 | 8.9666E-06 | 0.0011679 | 0.00086481 | CHGA/FGA/FGG/LEP/NEUROD1/TRH/CARTPT/CPLX2 | 8 |
|  | BP | GO:0046887 | positive regulation of hormone secretion | 5/43 | 131/18670 | 1.2243E-05 | 0.00141743 | 0.00104958 | FGA/FGG/LEP/TRH/CARTPT | 5 |
|  | BP | GO:0035296 | regulation of tube diameter | 5/43 | 143/18670 | 1.8716E-05 | 0.00155145 | 0.00114881 | CHGA/FGA/FGG/LEP/NTS | 5 |
|  | BP | GO:0050880 | regulation of blood vessel size | 5/43 | 143/18670 | 1.8716E-05 | 0.00155145 | 0.00114881 | CHGA/FGA/FGG/LEP/NTS | 5 |
|  | BP | GO:0097746 | regulation of blood vessel diameter | 5/43 | 143/18670 | 1.8716E-05 | 0.00155145 | 0.00114881 | CHGA/FGA/FGG/LEP/NTS | 5 |
|  | BP | GO:0035150 | regulation of tube size | 5/43 | 144/18670 | 1.9356E-05 | 0.00155145 | 0.00114881 | CHGA/FGA/FGG/LEP/NTS | 5 |
|  | BP | GO:0051953 | negative regulation of amine transport | 3/43 | 27/18670 | 3.2027E-05 | 0.00222632 | 0.00164854 | CHGA/LEP/TRH | 3 |
|  | BP | GO:1903532 | positive regulation of secretion by cell | 7/43 | 399/18670 | 3.2049E-05 | 0.00222632 | 0.00164854 | FGA/FGG/LEP/ORM1/ORM2/TRH/CARTPT | 7 |
|  | BP | GO:0042730 | fibrinolysis | 3/43 | 28/18670 | 3.5812E-05 | 0.00233228 | 0.001727 | FGA/FGG/KRT1 | 3 |
|  | BP | GO:0097756 | negative regulation of blood vessel diameter | 4/43 | 84/18670 | 4.1163E-05 | 0.00239892 | 0.00177635 | CHGA/FGA/FGG/LEP | 4 |
|  | BP | GO:0032760 | positive regulation of tumor necrosis factor production | 4/43 | 86/18670 | 4.5152E-05 | 0.00239892 | 0.00177635 | LBP/LEP/ORM1/ORM2 | 4 |
|  | BP | GO:0003018 | vascular process in circulatory system | 5/43 | 173/18670 | 4.6667E-05 | 0.00239892 | 0.00177635 | CHGA/FGA/FGG/LEP/NTS | 5 |
|  | BP | GO:1903557 | positive regulation of tumor necrosis factor superfamily cytokine production | 4/43 | 88/18670 | 4.9416E-05 | 0.00239892 | 0.00177635 | LBP/LEP/ORM1/ORM2 | 4 |
|  | BP | GO:0051047 | positive regulation of secretion | 7/43 | 428/18670 | 5.0031E-05 | 0.00239892 | 0.00177635 | FGA/FGG/LEP/ORM1/ORM2/TRH/CARTPT | 7 |
|  | BP | GO:0050796 | regulation of insulin secretion | 5/43 | 176/18670 | 5.0649E-05 | 0.00239892 | 0.00177635 | CHGA/LEP/NEUROD1/TRH/CARTPT | 5 |
|  | BP | GO:0051952 | regulation of amine transport | 4/43 | 95/18670 | 6.6681E-05 | 0.00302092 | 0.00223693 | CHGA/LEP/TRH/CARTPT | 4 |
|  | BP | GO:0015837 | amine transport | 4/43 | 102/18670 | 8.7975E-05 | 0.0038196 | 0.00282833 | CHGA/LEP/TRH/CARTPT | 4 |
|  | BP | GO:0030073 | insulin secretion | 5/43 | 207/18670 | 0.00010909 | 0.00454676 | 0.00336678 | CHGA/LEP/NEUROD1/TRH/CARTPT | 5 |
|  | BP | GO:0090278 | negative regulation of peptide hormone secretion | 3/43 | 45/18670 | 0.00015095 | 0.00604946 | 0.0044795 | CHGA/LEP/CARTPT | 3 |
|  | BP | GO:1904036 | negative regulation of epithelial cell apoptotic process | 3/43 | 46/18670 | 0.00016122 | 0.00622184 | 0.00460714 | FGA/FGG/NEUROD1 | 3 |
|  | BP | GO:0006953 | acute-phase response | 3/43 | 47/18670 | 0.00017193 | 0.00637157 | 0.00471801 | LBP/ORM1/ORM2 | 3 |
|  | BP | GO:0032102 | negative regulation of response to external stimulus | 6/43 | 365/18670 | 0.00017733 | 0.00637157 | 0.00471801 | FABP7/FGA/FGG/KRT1/LEP/CARTPT | 6 |
|  | BP | GO:0051048 | negative regulation of secretion | 5/43 | 238/18670 | 0.00020929 | 0.0071084 | 0.00526362 | CHGA/CSN2/LEP/TRH/CARTPT | 5 |
|  | BP | GO:0002576 | platelet degranulation | 4/43 | 128/18670 | 0.00021148 | 0.0071084 | 0.00526362 | FGA/FGG/ORM1/ORM2 | 4 |
|  | BP | GO:0008343 | adult feeding behavior | 2/43 | 10/18670 | 0.00023045 | 0.00727663 | 0.00538819 | LEP/CARTPT | 2 |
|  | BP | GO:0070091 | glucagon secretion | 2/43 | 10/18670 | 0.00023045 | 0.00727663 | 0.00538819 | LEP/CARTPT | 2 |
|  | BP | GO:0030195 | negative regulation of blood coagulation | 3/43 | 53/18670 | 0.00024602 | 0.00753972 | 0.005583 | FGA/FGG/KRT1 | 3 |
|  | BP | GO:1900047 | negative regulation of hemostasis | 3/43 | 54/18670 | 0.00026007 | 0.00774272 | 0.00573332 | FGA/FGG/KRT1 | 3 |
|  | BP | GO:0050819 | negative regulation of coagulation | 3/43 | 57/18670 | 0.00030532 | 0.00883737 | 0.00654389 | FGA/FGG/KRT1 | 3 |
|  | BP | GO:0019229 | regulation of vasoconstriction | 3/43 | 58/18670 | 0.00032146 | 0.00900409 | 0.00666734 | FGA/FGG/LEP | 3 |
|  | BP | GO:0030534 | adult behavior | 4/43 | 144/18670 | 0.00033159 | 0.00900409 | 0.00666734 | LEP/TRH/CARTPT/SEZ6 | 4 |
|  | BP | GO:0051956 | negative regulation of amino acid transport | 2/43 | 12/18670 | 0.00033701 | 0.00900409 | 0.00666734 | LEP/TRH | 2 |
|  | BP | GO:0050714 | positive regulation of protein secretion | 5/43 | 268/18670 | 0.00036183 | 0.00933635 | 0.00691337 | FGA/FGG/ORM1/ORM2/TRH | 5 |
|  | BP | GO:0051222 | positive regulation of protein transport | 6/43 | 418/18670 | 0.00036736 | 0.00933635 | 0.00691337 | FGA/FGG/LEP/ORM1/ORM2/TRH | 6 |
|  | BP | GO:0032096 | negative regulation of response to food | 2/43 | 13/18670 | 0.0003977 | 0.00963721 | 0.00713615 | LEP/CARTPT | 2 |
|  | BP | GO:0032099 | negative regulation of appetite | 2/43 | 13/18670 | 0.0003977 | 0.00963721 | 0.00713615 | LEP/CARTPT | 2 |
|  | BP | GO:0046888 | negative regulation of hormone secretion | 3/43 | 66/18670 | 0.00047066 | 0.01112433 | 0.00823733 | CHGA/LEP/CARTPT | 3 |
|  | BP | GO:0032680 | regulation of tumor necrosis factor production | 4/43 | 160/18670 | 0.00049418 | 0.01112433 | 0.00823733 | LBP/LEP/ORM1/ORM2 | 4 |
|  | BP | GO:0002793 | positive regulation of peptide secretion | 5/43 | 288/18670 | 0.00050259 | 0.01112433 | 0.00823733 | FGA/FGG/ORM1/ORM2/TRH | 5 |
|  | BP | GO:0032640 | tumor necrosis factor production | 4/43 | 163/18670 | 0.00053002 | 0.01112433 | 0.00823733 | LBP/LEP/ORM1/ORM2 | 4 |
|  | BP | GO:1903555 | regulation of tumor necrosis factor superfamily cytokine production | 4/43 | 163/18670 | 0.00053002 | 0.01112433 | 0.00823733 | LBP/LEP/ORM1/ORM2 | 4 |
|  | BP | GO:0032105 | negative regulation of response to extracellular stimulus | 2/43 | 15/18670 | 0.0005338 | 0.01112433 | 0.00823733 | LEP/CARTPT | 2 |
|  | BP | GO:0032108 | negative regulation of response to nutrient levels | 2/43 | 15/18670 | 0.0005338 | 0.01112433 | 0.00823733 | LEP/CARTPT | 2 |
|  | BP | GO:1904951 | positive regulation of establishment of protein localization | 6/43 | 456/18670 | 0.00058207 | 0.01189247 | 0.00880612 | FGA/FGG/LEP/ORM1/ORM2/TRH | 6 |
|  | BP | GO:0071706 | tumor necrosis factor superfamily cytokine production | 4/43 | 168/18670 | 0.00059381 | 0.01189895 | 0.00881092 | LBP/LEP/ORM1/ORM2 | 4 |
|  | BP | GO:0034116 | positive regulation of heterotypic cell-cell adhesion | 2/43 | 16/18670 | 0.00060916 | 0.01197636 | 0.00886824 | FGA/FGG | 2 |
|  | BP | GO:1903524 | positive regulation of blood circulation | 3/43 | 73/18670 | 0.00063258 | 0.01220645 | 0.00903862 | CHGA/FGA/FGG | 3 |
|  | BP | GO:0042310 | vasoconstriction | 3/43 | 76/18670 | 0.00071158 | 0.01324044 | 0.00980426 | FGA/FGG/LEP | 3 |
|  | BP | GO:0061045 | negative regulation of wound healing | 3/43 | 76/18670 | 0.00071158 | 0.01324044 | 0.00980426 | FGA/FGG/KRT1 | 3 |
|  | BP | GO:0030193 | regulation of blood coagulation | 3/43 | 79/18670 | 0.00079661 | 0.01443589 | 0.01068947 | FGA/FGG/KRT1 | 3 |
|  | BP | GO:0048871 | multicellular organismal homeostasis | 6/43 | 485/18670 | 0.00080353 | 0.01443589 | 0.01068947 | KRT1/LEP/MUC2/NEUROD1/UCP1/CARTPT | 6 |
|  | BP | GO:1900046 | regulation of hemostasis | 3/43 | 80/18670 | 0.00082632 | 0.01459363 | 0.01080627 | FGA/FGG/KRT1 | 3 |
|  | BP | GO:0032095 | regulation of response to food | 2/43 | 19/18670 | 0.00086426 | 0.01500926 | 0.01111404 | LEP/CARTPT | 2 |
|  | BP | GO:0050818 | regulation of coagulation | 3/43 | 84/18670 | 0.0009522 | 0.01626543 | 0.01204421 | FGA/FGG/KRT1 | 3 |
|  | BP | GO:0030277 | maintenance of gastrointestinal epithelium | 2/43 | 21/18670 | 0.00105827 | 0.01720291 | 0.01273839 | MUC2/NEUROD1 | 2 |
|  | BP | GO:0032891 | negative regulation of organic acid transport | 2/43 | 21/18670 | 0.00105827 | 0.01720291 | 0.01273839 | LEP/TRH | 2 |
|  | BP | GO:1904469 | positive regulation of tumor necrosis factor secretion | 2/43 | 21/18670 | 0.00105827 | 0.01720291 | 0.01273839 | ORM1/ORM2 | 2 |
|  | BP | GO:0050829 | defense response to Gram-negative bacterium | 3/43 | 88/18670 | 0.00108963 | 0.01720291 | 0.01273839 | CHGA/LALBA/LBP | 3 |
|  | BP | GO:1904035 | regulation of epithelial cell apoptotic process | 3/43 | 88/18670 | 0.00108963 | 0.01720291 | 0.01273839 | FGA/FGG/NEUROD1 | 3 |
|  | BP | GO:1903035 | negative regulation of response to wounding | 3/43 | 90/18670 | 0.0011628 | 0.01808409 | 0.01339089 | FGA/FGG/KRT1 | 3 |
|  | BP | GO:0032098 | regulation of appetite | 2/43 | 23/18670 | 0.00127124 | 0.01947994 | 0.01442448 | LEP/CARTPT | 2 |
|  | BP | GO:1903531 | negative regulation of secretion by cell | 4/43 | 211/18670 | 0.00138568 | 0.02085043 | 0.0154393 | CHGA/LEP/TRH/CARTPT | 4 |
|  | BP | GO:0090277 | positive regulation of peptide hormone secretion | 3/43 | 96/18670 | 0.0014007 | 0.02085043 | 0.0154393 | FGA/FGG/TRH | 3 |
|  | BP | GO:0031639 | plasminogen activation | 2/43 | 25/18670 | 0.00150301 | 0.02145387 | 0.01588613 | FGA/FGG | 2 |
|  | BP | GO:0032104 | regulation of response to extracellular stimulus | 2/43 | 25/18670 | 0.00150301 | 0.02145387 | 0.01588613 | LEP/CARTPT | 2 |
|  | BP | GO:0032107 | regulation of response to nutrient levels | 2/43 | 25/18670 | 0.00150301 | 0.02145387 | 0.01588613 | LEP/CARTPT | 2 |
|  | BP | GO:0022600 | digestive system process | 3/43 | 100/18670 | 0.0015751 | 0.02200217 | 0.01629214 | LEP/MUC2/NEUROD1 | 3 |
|  | BP | GO:0007631 | feeding behavior | 3/43 | 101/18670 | 0.00162072 | 0.02200217 | 0.01629214 | LEP/TRH/CARTPT | 3 |
|  | BP | GO:0050830 | defense response to Gram-positive bacterium | 3/43 | 101/18670 | 0.00162072 | 0.02200217 | 0.01629214 | CHGA/LALBA/LBP | 3 |
|  | BP | GO:0034114 | regulation of heterotypic cell-cell adhesion | 2/43 | 26/18670 | 0.00162588 | 0.02200217 | 0.01629214 | FGA/FGG | 2 |
|  | BP | GO:0002021 | response to dietary excess | 2/43 | 27/18670 | 0.00175339 | 0.02312697 | 0.01712503 | LEP/UCP1 | 2 |
|  | BP | GO:0072378 | blood coagulation, fibrin clot formation | 2/43 | 27/18670 | 0.00175339 | 0.02312697 | 0.01712503 | FGA/FGG | 2 |
|  | BP | GO:0001894 | tissue homeostasis | 4/43 | 227/18670 | 0.00181119 | 0.0235908 | 0.01746849 | KRT1/MUC2/NEUROD1/CARTPT | 4 |
|  | BP | GO:1903792 | negative regulation of anion transport | 2/43 | 28/18670 | 0.00188551 | 0.02425557 | 0.01796074 | LEP/TRH | 2 |
|  | BP | GO:0010669 | epithelial structure maintenance | 2/43 | 29/18670 | 0.00202222 | 0.02569703 | 0.01902811 | MUC2/NEUROD1 | 2 |
|  | BP | GO:1904019 | epithelial cell apoptotic process | 3/43 | 111/18670 | 0.00212304 | 0.02665307 | 0.01973604 | FGA/FGG/NEUROD1 | 3 |
|  | BP | GO:0045907 | positive regulation of vasoconstriction | 2/43 | 32/18670 | 0.00245971 | 0.03015311 | 0.02232774 | FGA/FGG | 2 |
|  | BP | GO:1904467 | regulation of tumor necrosis factor secretion | 2/43 | 32/18670 | 0.00245971 | 0.03015311 | 0.02232774 | ORM1/ORM2 | 2 |
|  | BP | GO:0010951 | negative regulation of endopeptidase activity | 4/43 | 250/18670 | 0.00257095 | 0.03027103 | 0.02241506 | CSN2/MAGEA3/SMR3B/SMR3A | 4 |
|  | BP | GO:0042755 | eating behavior | 2/43 | 33/18670 | 0.00261458 | 0.03027103 | 0.02241506 | LEP/TRH | 2 |
|  | BP | GO:0050718 | positive regulation of interleukin-1 beta secretion | 2/43 | 33/18670 | 0.00261458 | 0.03027103 | 0.02241506 | ORM1/ORM2 | 2 |
|  | BP | GO:0051955 | regulation of amino acid transport | 2/43 | 33/18670 | 0.00261458 | 0.03027103 | 0.02241506 | LEP/TRH | 2 |
|  | BP | GO:2000352 | negative regulation of endothelial cell apoptotic process | 2/43 | 33/18670 | 0.00261458 | 0.03027103 | 0.02241506 | FGA/FGG | 2 |
|  | BP | GO:0051954 | positive regulation of amine transport | 2/43 | 35/18670 | 0.00293777 | 0.03327347 | 0.0246383 | TRH/CARTPT | 2 |
|  | BP | GO:1990774 | tumor necrosis factor secretion | 2/43 | 35/18670 | 0.00293777 | 0.03327347 | 0.0246383 | ORM1/ORM2 | 2 |
|  | BP | GO:0010466 | negative regulation of peptidase activity | 4/43 | 262/18670 | 0.00304346 | 0.0340999 | 0.02525025 | CSN2/MAGEA3/SMR3B/SMR3A | 4 |
|  | BP | GO:1900026 | positive regulation of substrate adhesion-dependent cell spreading | 2/43 | 37/18670 | 0.00327875 | 0.03634534 | 0.02691296 | FGA/FGG | 2 |
|  | BP | GO:0032094 | response to food | 2/43 | 38/18670 | 0.00345587 | 0.03751054 | 0.02777576 | LEP/CARTPT | 2 |
|  | BP | GO:0050716 | positive regulation of interleukin-1 secretion | 2/43 | 38/18670 | 0.00345587 | 0.03751054 | 0.02777576 | ORM1/ORM2 | 2 |
|  | BP | GO:0051930 | regulation of sensory perception of pain | 2/43 | 39/18670 | 0.00363736 | 0.03864357 | 0.02861474 | SMR3B/SMR3A | 2 |
|  | BP | GO:1902042 | negative regulation of extrinsic apoptotic signaling pathway via death domain receptors | 2/43 | 39/18670 | 0.00363736 | 0.03864357 | 0.02861474 | FGA/FGG | 2 |
|  | BP | GO:0045088 | regulation of innate immune response | 5/43 | 452/18670 | 0.00367151 | 0.03864357 | 0.02861474 | FGA/FGG/LBP/LEP/MUC2 | 5 |
|  | BP | GO:0044058 | regulation of digestive system process | 2/43 | 40/18670 | 0.00382323 | 0.03932454 | 0.02911899 | LEP/NEUROD1 | 2 |
|  | BP | GO:0051931 | regulation of sensory perception | 2/43 | 40/18670 | 0.00382323 | 0.03932454 | 0.02911899 | SMR3B/SMR3A | 2 |
|  | BP | GO:0031644 | regulation of neurological system process | 3/43 | 137/18670 | 0.00384943 | 0.03932454 | 0.02911899 | CARTPT/SMR3B/SMR3A | 3 |
|  | BP | GO:0007586 | digestion | 3/43 | 139/18670 | 0.00400897 | 0.03982859 | 0.02949223 | LEP/MUC2/NEUROD1 | 3 |
|  | BP | GO:0001580 | detection of chemical stimulus involved in sensory perception of bitter taste | 2/43 | 41/18670 | 0.00401344 | 0.03982859 | 0.02949223 | RTP3/GNAT3 | 2 |
|  | BP | GO:0050873 | brown fat cell differentiation | 2/43 | 41/18670 | 0.00401344 | 0.03982859 | 0.02949223 | LEP/UCP1 | 2 |
|  | BP | GO:0002224 | toll-like receptor signaling pathway | 3/43 | 146/18670 | 0.00459895 | 0.04520857 | 0.03347599 | FGA/FGG/LBP | 3 |
|  | BP | GO:1903522 | regulation of blood circulation | 4/43 | 297/18670 | 0.00475505 | 0.04522225 | 0.03348612 | CHGA/FGA/FGG/LEP | 4 |
|  | BP | GO:0002792 | negative regulation of peptide secretion | 3/43 | 148/18670 | 0.00477666 | 0.04522225 | 0.03348612 | CHGA/LEP/CARTPT | 3 |
|  | BP | GO:0061041 | regulation of wound healing | 3/43 | 148/18670 | 0.00477666 | 0.04522225 | 0.03348612 | FGA/FGG/KRT1 | 3 |
|  | BP | GO:0002758 | innate immune response-activating signal transduction | 4/43 | 298/18670 | 0.00481177 | 0.04522225 | 0.03348612 | FGA/FGG/LBP/MUC2 | 4 |
|  | BP | GO:0050913 | sensory perception of bitter taste | 2/43 | 45/18670 | 0.00481734 | 0.04522225 | 0.03348612 | RTP3/GNAT3 | 2 |
|  | BP | GO:0050706 | regulation of interleukin-1 beta secretion | 2/43 | 46/18670 | 0.00502898 | 0.04678751 | 0.03464516 | ORM1/ORM2 | 2 |
|  | BP | GO:0043303 | mast cell degranulation | 2/43 | 47/18670 | 0.00524485 | 0.04793979 | 0.0354984 | CHGA/CPLX2 | 2 |
|  | BP | GO:0050912 | detection of chemical stimulus involved in sensory perception of taste | 2/43 | 47/18670 | 0.00524485 | 0.04793979 | 0.0354984 | RTP3/GNAT3 | 2 |
|  | BP | GO:0002279 | mast cell activation involved in immune response | 2/43 | 48/18670 | 0.00546493 | 0.04867056 | 0.03603952 | CHGA/CPLX2 | 2 |
|  | BP | GO:0007595 | lactation | 2/43 | 48/18670 | 0.00546493 | 0.04867056 | 0.03603952 | CSN2/CSN3 | 2 |
|  | BP | GO:0046850 | regulation of bone remodeling | 2/43 | 48/18670 | 0.00546493 | 0.04867056 | 0.03603952 | LEP/CARTPT | 2 |
|  | BP | GO:0002448 | mast cell mediated immunity | 2/43 | 49/18670 | 0.00568919 | 0.05023846 | 0.03720051 | CHGA/CPLX2 | 2 |
|  | BP | GO:0009409 | response to cold | 2/43 | 50/18670 | 0.00591762 | 0.0518165 | 0.03836902 | TRH/UCP1 | 2 |
|  | BP | GO:0032635 | interleukin-6 production | 3/43 | 161/18670 | 0.00603322 | 0.05238844 | 0.03879253 | LBP/LEP/ORM1 | 3 |
|  | BP | GO:0002218 | activation of innate immune response | 4/43 | 319/18670 | 0.00611039 | 0.05262007 | 0.03896405 | FGA/FGG/LBP/MUC2 | 4 |
|  | BP | GO:0032731 | positive regulation of interleukin-1 beta production | 2/43 | 52/18670 | 0.00638691 | 0.05410702 | 0.04006511 | ORM1/ORM2 | 2 |
|  | BP | GO:1900024 | regulation of substrate adhesion-dependent cell spreading | 2/43 | 52/18670 | 0.00638691 | 0.05410702 | 0.04006511 | FGA/FGG | 2 |
|  | BP | GO:0031638 | zymogen activation | 2/43 | 53/18670 | 0.00662773 | 0.0548103 | 0.04058587 | FGA/FGG | 2 |
|  | BP | GO:0050702 | interleukin-1 beta secretion | 2/43 | 53/18670 | 0.00662773 | 0.0548103 | 0.04058587 | ORM1/ORM2 | 2 |
|  | BP | GO:0050704 | regulation of interleukin-1 secretion | 2/43 | 53/18670 | 0.00662773 | 0.0548103 | 0.04058587 | ORM1/ORM2 | 2 |
|  | BP | GO:0016485 | protein processing | 4/43 | 328/18670 | 0.00673216 | 0.05523548 | 0.04090071 | FGA/FGG/MAGEA3/PCSK1 | 4 |
|  | BP | GO:0042742 | defense response to bacterium | 4/43 | 330/18670 | 0.00687583 | 0.05597351 | 0.0414472 | CHGA/FGA/LALBA/LBP | 4 |
|  | BP | GO:1902041 | regulation of extrinsic apoptotic signaling pathway via death domain receptors | 2/43 | 58/18670 | 0.00789281 | 0.06343666 | 0.04697351 | FGA/FGG | 2 |
|  | BP | GO:1903034 | regulation of response to wounding | 3/43 | 179/18670 | 0.0080728 | 0.06343666 | 0.04697351 | FGA/FGG/KRT1 | 3 |
|  | BP | GO:0032732 | positive regulation of interleukin-1 production | 2/43 | 59/18670 | 0.00815788 | 0.06343666 | 0.04697351 | ORM1/ORM2 | 2 |
|  | BP | GO:0032890 | regulation of organic acid transport | 2/43 | 59/18670 | 0.00815788 | 0.06343666 | 0.04697351 | LEP/TRH | 2 |
|  | BP | GO:0070527 | platelet aggregation | 2/43 | 59/18670 | 0.00815788 | 0.06343666 | 0.04697351 | FGA/FGG | 2 |
|  | BP | GO:2000351 | regulation of endothelial cell apoptotic process | 2/43 | 59/18670 | 0.00815788 | 0.06343666 | 0.04697351 | FGA/FGG | 2 |
|  | BP | GO:0045576 | mast cell activation | 2/43 | 60/18670 | 0.00842693 | 0.06472312 | 0.0479261 | CHGA/CPLX2 | 2 |
|  | BP | GO:0008217 | regulation of blood pressure | 3/43 | 182/18670 | 0.00844755 | 0.06472312 | 0.0479261 | CHGA/LEP/CARTPT | 3 |
|  | BP | GO:0034113 | heterotypic cell-cell adhesion | 2/43 | 61/18670 | 0.00869994 | 0.06569088 | 0.04864271 | FGA/FGG | 2 |
|  | BP | GO:0050701 | interleukin-1 secretion | 2/43 | 61/18670 | 0.00869994 | 0.06569088 | 0.04864271 | ORM1/ORM2 | 2 |
|  | BP | GO:0050433 | regulation of catecholamine secretion | 2/43 | 62/18670 | 0.00897689 | 0.0672944 | 0.04983008 | CHGA/CARTPT | 2 |
|  | BP | GO:0050432 | catecholamine secretion | 2/43 | 64/18670 | 0.00954253 | 0.07055901 | 0.05224745 | CHGA/CARTPT | 2 |
|  | BP | GO:0045861 | negative regulation of proteolysis | 4/43 | 363/18670 | 0.00954781 | 0.07055901 | 0.05224745 | CSN2/MAGEA3/SMR3B/SMR3A | 4 |
|  | BP | GO:0072577 | endothelial cell apoptotic process | 2/43 | 65/18670 | 0.00983118 | 0.07214149 | 0.05341925 | FGA/FGG | 2 |
|  | BP | GO:0050909 | sensory perception of taste | 2/43 | 67/18670 | 0.01042006 | 0.07579295 | 0.05612307 | RTP3/GNAT3 | 2 |
|  | BP | GO:0002221 | pattern recognition receptor signaling pathway | 3/43 | 197/18670 | 0.01047427 | 0.07579295 | 0.05612307 | FGA/FGG/LBP | 3 |
|  | BP | GO:0072376 | protein activation cascade | 3/43 | 198/18670 | 0.01061855 | 0.07630708 | 0.05650378 | FGA/FGG/KRT1 | 3 |
|  | BP | GO:0045089 | positive regulation of innate immune response | 4/43 | 381/18670 | 0.01125632 | 0.08032641 | 0.05948001 | FGA/FGG/LBP/MUC2 | 4 |
|  | BP | GO:0032720 | negative regulation of tumor necrosis factor production | 2/43 | 70/18670 | 0.01133204 | 0.08032641 | 0.05948001 | LBP/ORM1 | 2 |
|  | BP | GO:0002532 | production of molecular mediator involved in inflammatory response | 2/43 | 72/18670 | 0.01195891 | 0.08314705 | 0.06156863 | LBP/LEP | 2 |
|  | BP | GO:1903556 | negative regulation of tumor necrosis factor superfamily cytokine production | 2/43 | 72/18670 | 0.01195891 | 0.08314705 | 0.06156863 | LBP/ORM1 | 2 |
|  | BP | GO:0034284 | response to monosaccharide | 3/43 | 207/18670 | 0.01196934 | 0.08314705 | 0.06156863 | LEP/NEUROD1/TRH | 3 |
|  | BP | GO:0032418 | lysosome localization | 2/43 | 74/18670 | 0.01260073 | 0.08695341 | 0.06438716 | CHGA/CPLX2 | 2 |
|  | BP | GO:0051604 | protein maturation | 4/43 | 397/18670 | 0.01293176 | 0.08865063 | 0.06564391 | FGA/FGG/MAGEA3/PCSK1 | 4 |
|  | BP | GO:0017157 | regulation of exocytosis | 3/43 | 217/18670 | 0.01358153 | 0.09136776 | 0.06765589 | FGA/FGG/CPLX2 | 3 |
|  | BP | GO:0008344 | adult locomotory behavior | 2/43 | 77/18670 | 0.01359117 | 0.09136776 | 0.06765589 | TRH/SEZ6 | 2 |
|  | BP | GO:0051937 | catecholamine transport | 2/43 | 77/18670 | 0.01359117 | 0.09136776 | 0.06765589 | CHGA/CARTPT | 2 |
|  | BP | GO:0002526 | acute inflammatory response | 3/43 | 220/18670 | 0.01408822 | 0.09410211 | 0.06968062 | LBP/ORM1/ORM2 | 3 |
|  | BP | GO:0034109 | homotypic cell-cell adhesion | 2/43 | 81/18670 | 0.01496273 | 0.09930676 | 0.07353455 | FGA/FGG | 2 |
|  | CC | GO:0031093 | platelet alpha granule lumen | 4/46 | 67/19717 | 1.7846E-05 | 0.00151016 | 0.00133482 | FGA/FGG/ORM1/ORM2 | 4 |
|  | CC | GO:0072562 | blood microparticle | 5/46 | 147/19717 | 2.3056E-05 | 0.00151016 | 0.00133482 | FGA/FGG/KRT1/ORM1/ORM2 | 5 |
|  | CC | GO:0031091 | platelet alpha granule | 4/46 | 91/19717 | 5.9736E-05 | 0.00260845 | 0.00230558 | FGA/FGG/ORM1/ORM2 | 4 |
|  | CC | GO:0062023 | collagen-containing extracellular matrix | 6/46 | 406/19717 | 0.00034287 | 0.01122914 | 0.00992531 | FGA/FGG/KRT1/MUC2/ORM1/ORM2 | 6 |
|  | CC | GO:0034774 | secretory granule lumen | 5/46 | 321/19717 | 0.00087935 | 0.01955191 | 0.01728172 | FGA/FGG/ORM1/ORM2/PCSK1 | 5 |
|  | CC | GO:0060205 | cytoplasmic vesicle lumen | 5/46 | 338/19717 | 0.00110698 | 0.01955191 | 0.01728172 | FGA/FGG/ORM1/ORM2/PCSK1 | 5 |
|  | CC | GO:0031983 | vesicle lumen | 5/46 | 339/19717 | 0.00112161 | 0.01955191 | 0.01728172 | FGA/FGG/ORM1/ORM2/PCSK1 | 5 |
|  | CC | GO:0042629 | mast cell granule | 2/46 | 22/19717 | 0.00119401 | 0.01955191 | 0.01728172 | CHGA/CPLX2 | 2 |
|  | MF | GO:0005179 | hormone activity | 5/34 | 122/17697 | 3.3998E-06 | 0.00033658 | 0.00026483 | CHGB/LEP/NTS/TRH/CARTPT | 5 |
|  | MF | GO:0005184 | neuropeptide hormone activity | 2/34 | 28/17697 | 0.00131252 | 0.06496988 | 0.05111931 | NTS/CARTPT | 2 |
|  | MF | GO:0048018 | receptor ligand activity | 5/34 | 482/17697 | 0.00212703 | 0.070192 | 0.05522816 | CHGB/LEP/NTS/TRH/CARTPT | 5 |
|  | MF | GO:0070888 | E-box binding | 2/34 | 50/17697 | 0.00414258 | 0.071634 | 0.05636274 | MYF6/NEUROD1 | 2 |
|  | MF | GO:0004866 | endopeptidase inhibitor activity | 3/34 | 175/17697 | 0.00454107 | 0.071634 | 0.05636274 | CSN2/SMR3B/SMR3A | 3 |
|  | MF | GO:0030414 | peptidase inhibitor activity | 3/34 | 182/17697 | 0.00506503 | 0.071634 | 0.05636274 | CSN2/SMR3B/SMR3A | 3 |
|  | MF | GO:0061135 | endopeptidase regulator activity | 3/34 | 182/17697 | 0.00506503 | 0.071634 | 0.05636274 | CSN2/SMR3B/SMR3A | 3 |
|  |  |  |  |  |  |  |  |  |  |  |
| DLBC | BP | GO:0034472 | snRNA 3'-end processing | 3/25 | 30/18670 | 8.4077E-06 | 0.00552273 | 0.00388592 | CT45A3/CT45A1/CT45A10 | 3 |
|  | BP | GO:0016180 | snRNA processing | 3/25 | 36/18670 | 1.4708E-05 | 0.00552273 | 0.00388592 | CT45A3/CT45A1/CT45A10 | 3 |
|  | BP | GO:0043628 | ncRNA 3'-end processing | 3/25 | 48/18670 | 3.5252E-05 | 0.00882479 | 0.00620933 | CT45A3/CT45A1/CT45A10 | 3 |
|  | BP | GO:0016073 | snRNA metabolic process | 3/25 | 101/18670 | 0.00032413 | 0.06085484 | 0.04281888 | CT45A3/CT45A1/CT45A10 | 3 |
|  | CC | GO:0032039 | integrator complex | 3/27 | 28/19717 | 7.3325E-06 | 0.00032263 | 0.00023927 | CT45A3/CT45A1/CT45A10 | 3 |
|  | CC | GO:0042571 | immunoglobulin complex, circulating | 2/27 | 72/19717 | 0.00435099 | 0.0957218 | 0.07098985 | IGLC6/IGLC7 | 2 |
|  |  |  |  |  |  |  |  |  |  |  |
| ESCA | BP | GO:0007586 | digestion | 5/41 | 139/18670 | 1.2856E-05 | 0.01045191 | 0.00860674 | PGA5/PGC/CHIA/GKN1/PGA3 | 5 |
|  | BP | GO:0071320 | cellular response to cAMP | 3/41 | 53/18670 | 0.00021336 | 0.05627217 | 0.04633793 | CPS1/SLC26A3/PCK1 | 3 |
|  | BP | GO:0030007 | cellular potassium ion homeostasis | 2/41 | 13/18670 | 0.00036143 | 0.05627217 | 0.04633793 | ATP4A/ATP4B | 2 |
|  | BP | GO:0010248 | establishment or maintenance of transmembrane electrochemical gradient | 2/41 | 14/18670 | 0.00042108 | 0.05627217 | 0.04633793 | ATP4A/ATP4B | 2 |
|  | BP | GO:0036376 | sodium ion export across plasma membrane | 2/41 | 14/18670 | 0.00042108 | 0.05627217 | 0.04633793 | ATP4A/ATP4B | 2 |
|  | BP | GO:0009410 | response to xenobiotic stimulus | 5/41 | 292/18670 | 0.00042732 | 0.05627217 | 0.04633793 | CPS1/PCK1/S100A12/UGT1A8/PPP1R1B | 5 |
|  | BP | GO:0070365 | hepatocyte differentiation | 2/41 | 15/18670 | 0.00048518 | 0.05627217 | 0.04633793 | CPS1/PCK1 | 2 |
|  | BP | GO:0007158 | neuron cell-cell adhesion | 2/41 | 16/18670 | 0.00055372 | 0.05627217 | 0.04633793 | ASTN1/NRXN1 | 2 |
|  | BP | GO:0006883 | cellular sodium ion homeostasis | 2/41 | 17/18670 | 0.00062668 | 0.05661019 | 0.04661628 | ATP4A/ATP4B | 2 |
|  | CC | GO:0031904 | endosome lumen | 3/44 | 34/19717 | 5.9117E-05 | 0.00656375 | 0.00484759 | GH2/PGA5/PGA3 | 3 |
|  | CC | GO:0043025 | neuronal cell body | 7/44 | 497/19717 | 0.00010587 | 0.00656375 | 0.00484759 | ASTN1/GABRA5/GHRH/GRIK3/NRXN1/PPP1R1B/BRINP3 | 7 |
|  | CC | GO:0042734 | presynaptic membrane | 4/44 | 161/19717 | 0.00045079 | 0.01863269 | 0.01376098 | GABRA5/GRIK3/NRXN1/KCTD8 | 4 |
|  | CC | GO:0043204 | perikaryon | 3/44 | 134/19717 | 0.00331637 | 0.09671064 | 0.07142467 | ASTN1/GHRH/GRIK3 | 3 |
|  | CC | GO:0098793 | presynapse | 5/44 | 491/19717 | 0.00457872 | 0.09671064 | 0.07142467 | GABRA5/GHRH/GRIK3/NRXN1/KCTD8 | 5 |
|  | CC | GO:0005771 | multivesicular body | 2/44 | 51/19717 | 0.00578934 | 0.09671064 | 0.07142467 | PGA5/PGA3 | 2 |
|  | CC | GO:0043195 | terminal bouton | 2/44 | 51/19717 | 0.00578934 | 0.09671064 | 0.07142467 | GHRH/GRIK3 | 2 |
|  | CC | GO:0031526 | brush border membrane | 2/44 | 53/19717 | 0.0062394 | 0.09671064 | 0.07142467 | SLC26A3/ITLN1 | 2 |
|  | MF | GO:0004190 | aspartic-type endopeptidase activity | 3/37 | 25/17697 | 1.8746E-05 | 0.00192559 | 0.00128076 | PGA5/PGC/PGA3 | 3 |
|  | MF | GO:0070001 | aspartic-type peptidase activity | 3/37 | 26/17697 | 2.116E-05 | 0.00192559 | 0.00128076 | PGA5/PGC/PGA3 | 3 |
|  | MF | GO:0005391 | sodium:potassium-exchanging ATPase activity | 2/37 | 11/17697 | 0.00023117 | 0.01051837 | 0.00699603 | ATP4A/ATP4B | 2 |
|  | MF | GO:0008556 | potassium-transporting ATPase activity | 2/37 | 11/17697 | 0.00023117 | 0.01051837 | 0.00699603 | ATP4A/ATP4B | 2 |
|  | MF | GO:0015662 | ATPase activity, coupled to transmembrane movement of ions, phosphorylative mechanism | 2/37 | 33/17697 | 0.00215591 | 0.07350097 | 0.04888729 | ATP4A/ATP4B | 2 |
|  | MF | GO:0016831 | carboxy-lyase activity | 2/37 | 35/17697 | 0.00242311 | 0.07350097 | 0.04888729 | PCK1/GADL1 | 2 |
|  | MF | GO:0052689 | carboxylic ester hydrolase activity | 3/37 | 136/17697 | 0.00284946 | 0.07393247 | 0.04917429 | ACHE/LIPF/AADACL2 | 3 |
|  | MF | GO:0015081 | sodium ion transmembrane transporter activity | 3/37 | 149/17697 | 0.00368527 | 0.07393247 | 0.04917429 | ATP4A/ATP4B/GRIK3 | 3 |
|  | MF | GO:1904315 | transmitter-gated ion channel activity involved in regulation of postsynaptic membrane potential | 2/37 | 47/17697 | 0.00433349 | 0.07393247 | 0.04917429 | GABRA5/GRIK3 | 2 |
|  | MF | GO:0015079 | potassium ion transmembrane transporter activity | 3/37 | 159/17697 | 0.00442024 | 0.07393247 | 0.04917429 | ATP4A/ATP4B/GRIK3 | 3 |
|  | MF | GO:0099529 | neurotransmitter receptor activity involved in regulation of postsynaptic membrane potential | 2/37 | 50/17697 | 0.00489147 | 0.07393247 | 0.04917429 | GABRA5/GRIK3 | 2 |
|  | MF | GO:0016830 | carbon-carbon lyase activity | 2/37 | 51/17697 | 0.00508444 | 0.07393247 | 0.04917429 | PCK1/GADL1 | 2 |
|  | MF | GO:0098960 | postsynaptic neurotransmitter receptor activity | 2/37 | 52/17697 | 0.00528089 | 0.07393247 | 0.04917429 | GABRA5/GRIK3 | 2 |
|  | MF | GO:0022824 | transmitter-gated ion channel activity | 2/37 | 61/17697 | 0.00720264 | 0.081075 | 0.05392496 | GABRA5/GRIK3 | 2 |
|  | MF | GO:0022835 | transmitter-gated channel activity | 2/37 | 61/17697 | 0.00720264 | 0.081075 | 0.05392496 | GABRA5/GRIK3 | 2 |
|  | MF | GO:0031406 | carboxylic acid binding | 3/37 | 193/17697 | 0.00755599 | 0.081075 | 0.05392496 | CPS1/PCK1/UGT1A8 | 3 |
|  | MF | GO:0019829 | cation-transporting ATPase activity | 2/37 | 66/17697 | 0.00838735 | 0.081075 | 0.05392496 | ATP4A/ATP4B | 2 |
|  | MF | GO:0022853 | active ion transmembrane transporter activity | 2/37 | 67/17697 | 0.00863411 | 0.081075 | 0.05392496 | ATP4A/ATP4B | 2 |
|  | MF | GO:0042625 | ATPase coupled ion transmembrane transporter activity | 2/37 | 67/17697 | 0.00863411 | 0.081075 | 0.05392496 | ATP4A/ATP4B | 2 |
|  | MF | GO:0043177 | organic acid binding | 3/37 | 205/17697 | 0.00890934 | 0.081075 | 0.05392496 | CPS1/PCK1/UGT1A8 | 3 |
|  | MF | GO:0005230 | extracellular ligand-gated ion channel activity | 2/37 | 75/17697 | 0.01072375 | 0.09293921 | 0.06181613 | GABRA5/GRIK3 | 2 |
|  | MF | GO:0004175 | endopeptidase activity | 4/37 | 427/17697 | 0.01177076 | 0.09737631 | 0.06476736 | CPS1/PGA5/PGC/PGA3 | 4 |
|  |  |  |  |  |  |  |  |  |  |  |
| HNSC | BP | GO:0031424 | keratinization | 8/30 | 224/18670 | 1.767E-09 | 5.7073E-07 | 4.3523E-07 | KRT2/TGM3/KRT37/KRT24/LCE2D/LCE3B/HRNR/SPINK6 | 8 |
|  | BP | GO:0043588 | skin development | 9/30 | 419/18670 | 1.2491E-08 | 2.0173E-06 | 1.5384E-06 | KRT2/TGM3/KRT37/KRT24/LCE2D/LCE3B/HRNR/FLG2/SPINK6 | 9 |
|  | BP | GO:0030216 | keratinocyte differentiation | 8/30 | 305/18670 | 1.9821E-08 | 2.1341E-06 | 1.6274E-06 | KRT2/TGM3/KRT37/KRT24/LCE2D/LCE3B/HRNR/SPINK6 | 8 |
|  | BP | GO:0008544 | epidermis development | 9/30 | 464/18670 | 3.0121E-08 | 2.4322E-06 | 1.8548E-06 | KRT2/TGM3/KRT37/KRT24/LCE2D/LCE3B/HRNR/FLG2/SPINK6 | 9 |
|  | BP | GO:0009913 | epidermal cell differentiation | 8/30 | 358/18670 | 6.8452E-08 | 4.422E-06 | 3.3722E-06 | KRT2/TGM3/KRT37/KRT24/LCE2D/LCE3B/HRNR/SPINK6 | 8 |
|  | BP | GO:0018149 | peptide cross-linking | 4/30 | 60/18670 | 2.4808E-06 | 0.00013355 | 0.00010184 | KRT2/TGM3/LCE2D/LCE3B | 4 |
|  | BP | GO:0070268 | cornification | 4/30 | 112/18670 | 2.9817E-05 | 0.00137584 | 0.0010492 | KRT2/KRT37/KRT24/SPINK6 | 4 |
|  | BP | GO:0031640 | killing of cells of other organism | 3/30 | 62/18670 | 0.00013282 | 0.0047666 | 0.00363495 | DEFB4A/LCE3B/DEFB103A | 3 |
|  | BP | GO:0044364 | disruption of cells of other organism | 3/30 | 62/18670 | 0.00013282 | 0.0047666 | 0.00363495 | DEFB4A/LCE3B/DEFB103A | 3 |
|  | BP | GO:0050829 | defense response to Gram-negative bacterium | 3/30 | 88/18670 | 0.00037467 | 0.01210179 | 0.00922868 | DEFB4A/LCE3B/DEFB103A | 3 |
|  | BP | GO:0050830 | defense response to Gram-positive bacterium | 3/30 | 101/18670 | 0.00056104 | 0.01536446 | 0.01171675 | DEFB4A/LCE3B/DEFB103A | 3 |
|  | BP | GO:0016338 | calcium-independent cell-cell adhesion via plasma membrane cell-adhesion molecules | 2/30 | 23/18670 | 0.00061838 | 0.01536446 | 0.01171675 | CLDN17/CLDN19 | 2 |
|  | BP | GO:0061436 | establishment of skin barrier | 2/30 | 23/18670 | 0.00061838 | 0.01536446 | 0.01171675 | HRNR/FLG2 | 2 |
|  | BP | GO:0033561 | regulation of water loss via skin | 2/30 | 26/18670 | 0.00079199 | 0.01827235 | 0.01393427 | HRNR/FLG2 | 2 |
|  | BP | GO:0048730 | epidermis morphogenesis | 2/30 | 29/18670 | 0.00098642 | 0.02124091 | 0.01619806 | TGM3/FLG2 | 2 |
|  | BP | GO:0072595 | maintenance of protein localization in organelle | 2/30 | 39/18670 | 0.00178247 | 0.03598352 | 0.02744058 | NR5A1/PDIA2 | 2 |
|  | BP | GO:0043616 | keratinocyte proliferation | 2/30 | 43/18670 | 0.00216351 | 0.04073206 | 0.03106176 | KRT2/SLURP1 | 2 |
|  | BP | GO:0035821 | modification of morphology or physiology of other organism | 3/30 | 164/18670 | 0.0022699 | 0.04073206 | 0.03106176 | DEFB4A/LCE3B/DEFB103A | 3 |
|  | BP | GO:0001906 | cell killing | 3/30 | 168/18670 | 0.00243063 | 0.04132063 | 0.0315106 | DEFB4A/LCE3B/DEFB103A | 3 |
|  | BP | GO:0046503 | glycerolipid catabolic process | 2/30 | 64/18670 | 0.00473017 | 0.07639232 | 0.05825584 | FABP4/PLA2G4D | 2 |
|  | BP | GO:0050891 | multicellular organismal water homeostasis | 2/30 | 68/18670 | 0.00532368 | 0.07816131 | 0.05960485 | HRNR/FLG2 | 2 |
|  | BP | GO:0050918 | positive chemotaxis | 2/30 | 68/18670 | 0.00532368 | 0.07816131 | 0.05960485 | DEFB4A/DEFB103A | 2 |
|  | BP | GO:0030104 | water homeostasis | 2/30 | 74/18670 | 0.00627468 | 0.08444671 | 0.06439801 | HRNR/FLG2 | 2 |
|  | BP | GO:0032507 | maintenance of protein location in cell | 2/30 | 74/18670 | 0.00627468 | 0.08444671 | 0.06439801 | NR5A1/PDIA2 | 2 |
|  | BP | GO:0010951 | negative regulation of endopeptidase activity | 3/30 | 250/18670 | 0.00737533 | 0.09528927 | 0.07266642 | SPINK7/SERPINB12/SPINK6 | 3 |
|  | CC | GO:0001533 | cornified envelope | 5/32 | 65/19717 | 6.2576E-08 | 3.0037E-06 | 2.2396E-06 | KRT2/LCE2D/LCE3B/HRNR/FLG2 | 5 |
|  | MF | GO:0030280 | structural constituent of epidermis | 3/35 | 16/17697 | 3.8991E-06 | 0.00033922 | 0.00025447 | KRT2/HRNR/FLG2 | 3 |
|  | MF | GO:0004867 | serine-type endopeptidase inhibitor activity | 3/35 | 94/17697 | 0.00083976 | 0.03652942 | 0.02740259 | SPINK7/SERPINB12/SPINK6 | 3 |
|  | MF | GO:0042056 | chemoattractant activity | 2/35 | 38/17697 | 0.00255456 | 0.06833811 | 0.05126392 | DEFB4A/DEFB103A | 2 |
|  | MF | GO:0048020 | CCR chemokine receptor binding | 2/35 | 43/17697 | 0.00326106 | 0.06833811 | 0.05126392 | DEFB4A/DEFB103A | 2 |
|  | MF | GO:0004866 | endopeptidase inhibitor activity | 3/35 | 175/17697 | 0.0049311 | 0.06833811 | 0.05126392 | SPINK7/SERPINB12/SPINK6 | 3 |
|  | MF | GO:0030414 | peptidase inhibitor activity | 3/35 | 182/17697 | 0.00549847 | 0.06833811 | 0.05126392 | SPINK7/SERPINB12/SPINK6 | 3 |
|  | MF | GO:0061135 | endopeptidase regulator activity | 3/35 | 182/17697 | 0.00549847 | 0.06833811 | 0.05126392 | SPINK7/SERPINB12/SPINK6 | 3 |
|  | MF | GO:0042379 | chemokine receptor binding | 2/35 | 66/17697 | 0.00752915 | 0.0818795 | 0.061422 | DEFB4A/DEFB103A | 2 |
|  | MF | GO:0061134 | peptidase regulator activity | 3/35 | 219/17697 | 0.00914077 | 0.0883608 | 0.06628396 | SPINK7/SERPINB12/SPINK6 | 3 |
|  | KEGG | hsa05150 | Staphylococcus aureus infection | 4/13 | 96/8076 | 1.2351E-05 | 0.00049402 | 0.00039002 | DEFB4A/KRT37/KRT24/DEFB103A | 4 |
|  |  |  |  |  |  |  |  |  |  |  |
| KIRC | BP | GO:0042475 | odontogenesis of dentin-containing tooth | 4/34 | 90/18670 | 2.0963E-05 | 0.01163455 | 0.00944445 | SLC34A1/KLK4/ODAM/NKX2-3 | 4 |
|  | BP | GO:0042476 | odontogenesis | 4/34 | 132/18670 | 9.392E-05 | 0.02606281 | 0.02115673 | SLC34A1/KLK4/ODAM/NKX2-3 | 4 |
|  | CC | GO:0016323 | basolateral plasma membrane | 5/36 | 217/19717 | 4.4024E-05 | 0.00427033 | 0.0033829 | UMOD/CLDN8/SLC22A8/RHCG/SLC22A9 | 5 |
|  | CC | GO:0045177 | apical part of cell | 5/36 | 384/19717 | 0.00062608 | 0.03036499 | 0.02405474 | REN/SLC34A1/UMOD/RHCG/SLC6A19 | 5 |
|  | CC | GO:0016324 | apical plasma membrane | 4/36 | 318/19717 | 0.00260374 | 0.08418748 | 0.0666922 | SLC34A1/UMOD/RHCG/SLC6A19 | 4 |
|  | MF | GO:0004252 | serine-type endopeptidase activity | 6/32 | 160/17697 | 3.7091E-07 | 2.6933E-05 | 1.7955E-05 | KLK1/LPA/PLG/KLK4/TMPRSS11E/KLK15 | 6 |
|  | MF | GO:0004175 | endopeptidase activity | 8/32 | 427/17697 | 6.8076E-07 | 2.6933E-05 | 1.7955E-05 | KLK1/LPA/PLG/REN/KLK4/CASP14/TMPRSS11E/KLK15 | 8 |
|  | MF | GO:0008236 | serine-type peptidase activity | 6/32 | 182/17697 | 7.9053E-07 | 2.6933E-05 | 1.7955E-05 | KLK1/LPA/PLG/KLK4/TMPRSS11E/KLK15 | 6 |
|  | MF | GO:0017171 | serine hydrolase activity | 6/32 | 186/17697 | 8.9775E-07 | 2.6933E-05 | 1.7955E-05 | KLK1/LPA/PLG/KLK4/TMPRSS11E/KLK15 | 6 |
|  | MF | GO:0022804 | active transmembrane transporter activity | 6/32 | 362/17697 | 4.0616E-05 | 0.00097479 | 0.00064986 | SLC34A1/SLC13A2/SLC22A8/SLC22A9/ATP6V1G3/SLC6A19 | 6 |
|  | MF | GO:0015291 | secondary active transmembrane transporter activity | 5/32 | 237/17697 | 6.1897E-05 | 0.00123795 | 0.0008253 | SLC34A1/SLC13A2/SLC22A8/SLC22A9/SLC6A19 | 5 |
|  | MF | GO:0008509 | anion transmembrane transporter activity | 5/32 | 327/17697 | 0.00027918 | 0.00441393 | 0.00294262 | SLC34A1/SLC13A2/SLC22A8/SLC22A9/SLC6A19 | 5 |
|  | MF | GO:0015370 | solute:sodium symporter activity | 3/32 | 72/17697 | 0.00029426 | 0.00441393 | 0.00294262 | SLC34A1/SLC13A2/SLC6A19 | 3 |
|  | MF | GO:0034185 | apolipoprotein binding | 2/32 | 17/17697 | 0.00042356 | 0.00564745 | 0.00376497 | LPA/PLG | 2 |
|  | MF | GO:0015294 | solute:cation symporter activity | 3/32 | 102/17697 | 0.00081657 | 0.00979883 | 0.00653256 | SLC34A1/SLC13A2/SLC6A19 | 3 |
|  | MF | GO:0015293 | symporter activity | 3/32 | 144/17697 | 0.00220155 | 0.02241435 | 0.0149429 | SLC34A1/SLC13A2/SLC6A19 | 3 |
|  | MF | GO:0015081 | sodium ion transmembrane transporter activity | 3/32 | 149/17697 | 0.00242578 | 0.02241435 | 0.0149429 | SLC34A1/SLC13A2/SLC6A19 | 3 |
|  | MF | GO:0005342 | organic acid transmembrane transporter activity | 3/32 | 153/17697 | 0.00261501 | 0.02241435 | 0.0149429 | SLC13A2/SLC22A9/SLC6A19 | 3 |
|  | MF | GO:0046943 | carboxylic acid transmembrane transporter activity | 3/32 | 153/17697 | 0.00261501 | 0.02241435 | 0.0149429 | SLC13A2/SLC22A9/SLC6A19 | 3 |
|  | MF | GO:0015077 | monovalent inorganic cation transmembrane transporter activity | 4/32 | 382/17697 | 0.00476598 | 0.0381278 | 0.02541853 | SLC34A1/SLC13A2/ATP6V1G3/SLC6A19 | 4 |
|  | MF | GO:0008514 | organic anion transmembrane transporter activity | 3/32 | 211/17697 | 0.00642465 | 0.04818485 | 0.03212324 | SLC13A2/SLC22A9/SLC6A19 | 3 |
|  | MF | GO:0051117 | ATPase binding | 2/32 | 80/17697 | 0.00916776 | 0.06471357 | 0.04314238 | ALDOB/ATP6V1G3 | 2 |
|  | KEGG | hsa04614 | Renin-angiotensin system | 2/15 | 23/8076 | 0.00079654 | 0.04062378 | 0.03773107 | KLK1/REN | 2 |
|  |  |  |  |  |  |  |  |  |  |  |
| KIRP | CC | GO:0031528 | microvillus membrane | 2/39 | 23/19717 | 0.00093952 | 0.07422185 | 0.05834896 | DPEP1/PROM2 | 2 |
|  | MF | GO:0004252 | serine-type endopeptidase activity | 4/39 | 160/17697 | 0.00041365 | 0.03377454 | 0.02506554 | KLK3/HP/KLK6/TMPRSS4 | 4 |
|  | MF | GO:0008236 | serine-type peptidase activity | 4/39 | 182/17697 | 0.00067198 | 0.03377454 | 0.02506554 | KLK3/HP/KLK6/TMPRSS4 | 4 |
|  | MF | GO:0017171 | serine hydrolase activity | 4/39 | 186/17697 | 0.00072895 | 0.03377454 | 0.02506554 | KLK3/HP/KLK6/TMPRSS4 | 4 |
|  | MF | GO:0031490 | chromatin DNA binding | 3/39 | 119/17697 | 0.00227099 | 0.06505188 | 0.0482778 | ZIC2/PRDM14/GRHL2 | 3 |
|  | MF | GO:0004175 | endopeptidase activity | 5/39 | 427/17697 | 0.00234 | 0.06505188 | 0.0482778 | KLK3/HP/KLK6/CASP14/TMPRSS4 | 5 |
|  |  |  |  |  |  |  |  |  |  |  |
| LUAD | BP | GO:0070268 | cornification | 3/25 | 112/18670 | 0.00043901 | 0.0820159 | 0.06645528 | KLK13/KLK14/KLK12 | 3 |
|  | BP | GO:0019730 | antimicrobial humoral response | 3/25 | 122/18670 | 0.00056368 | 0.0820159 | 0.06645528 | FGB/SPAG11B/BPIFA1 | 3 |
|  | MF | GO:0005179 | hormone activity | 4/25 | 122/17697 | 2.4311E-05 | 0.00102108 | 0.00066536 | CALCA/CGA/NTS/SST | 4 |
|  | MF | GO:0004252 | serine-type endopeptidase activity | 3/25 | 160/17697 | 0.0014413 | 0.02326865 | 0.01516253 | KLK13/KLK14/KLK12 | 3 |
|  | MF | GO:0008236 | serine-type peptidase activity | 3/25 | 182/17697 | 0.00208313 | 0.02326865 | 0.01516253 | KLK13/KLK14/KLK12 | 3 |
|  | MF | GO:0017171 | serine hydrolase activity | 3/25 | 186/17697 | 0.00221606 | 0.02326865 | 0.01516253 | KLK13/KLK14/KLK12 | 3 |
|  | MF | GO:0048018 | receptor ligand activity | 4/25 | 482/17697 | 0.00436466 | 0.03666311 | 0.02389075 | CALCA/CGA/NTS/SST | 4 |
|  | MF | GO:0008190 | eukaryotic initiation factor 4E binding | 1/25 | 10/17697 | 0.01404077 | 0.09260635 | 0.06034499 | OTX2 | 1 |
|  | MF | GO:0045236 | CXCR chemokine receptor binding | 1/25 | 11/17697 | 0.01543439 | 0.09260635 | 0.06034499 | TFF2 | 1 |
|  | KEGG | hsa04080 | Neuroactive ligand-receptor interaction | 4/8 | 341/8076 | 0.00019103 | 0.01012443 | 0.00924972 | CALCA/CGA/NTS/SST | 4 |
|  | KEGG | hsa04024 | cAMP signaling pathway | 3/8 | 216/8076 | 0.0009567 | 0.02535257 | 0.02316222 | CGA/SST/CALML5 | 3 |
|  | KEGG | hsa04971 | Gastric acid secretion | 2/8 | 76/8076 | 0.00235912 | 0.04167785 | 0.03807708 | SST/CALML5 | 2 |
|  | KEGG | hsa04912 | GnRH signaling pathway | 2/8 | 93/8076 | 0.00351139 | 0.04652591 | 0.04250629 | CGA/CALML5 | 2 |
|  | KEGG | hsa04270 | Vascular smooth muscle contraction | 2/8 | 135/8076 | 0.00727072 | 0.07706967 | 0.07041122 | CALCA/CALML5 | 2 |
|  |  |  |  |  |  |  |  |  |  |  |
| PAAD | BP | GO:0007586 | digestion | 6/45 | 139/18670 | 9.8031E-07 | 0.00049898 | 0.00044269 | AMY2A/CEL/CLPS/PNLIP/PRSS1/CTRB2 | 6 |
|  | BP | GO:0044241 | lipid digestion | 3/45 | 19/18670 | 1.2341E-05 | 0.00314089 | 0.00278656 | CEL/CLPS/PNLIP | 3 |
|  | BP | GO:0009235 | cobalamin metabolic process | 3/45 | 22/18670 | 1.9515E-05 | 0.00331101 | 0.00293749 | PRSS1/CTRC/CTRB2 | 3 |
|  | BP | GO:0033013 | tetrapyrrole metabolic process | 3/45 | 60/18670 | 0.00040675 | 0.05175868 | 0.04591971 | PRSS1/CTRC/CTRB2 | 3 |
|  | BP | GO:0030299 | intestinal cholesterol absorption | 2/45 | 15/18670 | 0.00058468 | 0.05952075 | 0.05280613 | CEL/PNLIP | 2 |
|  | BP | GO:0098856 | intestinal lipid absorption | 2/45 | 17/18670 | 0.00075498 | 0.06404787 | 0.05682253 | CEL/PNLIP | 2 |
|  | BP | GO:0006767 | water-soluble vitamin metabolic process | 3/45 | 88/18670 | 0.0012444 | 0.09048599 | 0.08027813 | PRSS1/CTRC/CTRB2 | 3 |
|  | BP | GO:0002227 | innate immune response in mucosa | 2/45 | 24/18670 | 0.00151583 | 0.09644441 | 0.08556438 | DEFA6/PLA2G1B | 2 |
|  | MF | GO:0004252 | serine-type endopeptidase activity | 7/39 | 160/17697 | 5.2215E-08 | 4.8825E-06 | 3.7004E-06 | CTRL/PRSS1/CELA3A/CTRC/CELA3B/CELA2A/CTRB2 | 7 |
|  | MF | GO:0008236 | serine-type peptidase activity | 7/39 | 182/17697 | 1.2628E-07 | 4.8825E-06 | 3.7004E-06 | CTRL/PRSS1/CELA3A/CTRC/CELA3B/CELA2A/CTRB2 | 7 |
|  | MF | GO:0017171 | serine hydrolase activity | 7/39 | 186/17697 | 1.4648E-07 | 4.8825E-06 | 3.7004E-06 | CTRL/PRSS1/CELA3A/CTRC/CELA3B/CELA2A/CTRB2 | 7 |
|  | MF | GO:0004181 | metallocarboxypeptidase activity | 3/39 | 29/17697 | 3.4751E-05 | 0.00071512 | 0.00054198 | CPA1/CPA2/CPB1 | 3 |
|  | MF | GO:0004175 | endopeptidase activity | 7/39 | 427/17697 | 3.5756E-05 | 0.00071512 | 0.00054198 | CTRL/PRSS1/CELA3A/CTRC/CELA3B/CELA2A/CTRB2 | 7 |
|  | MF | GO:0004180 | carboxypeptidase activity | 3/39 | 45/17697 | 0.0001317 | 0.002195 | 0.00166358 | CPA1/CPA2/CPB1 | 3 |
|  | MF | GO:0008235 | metalloexopeptidase activity | 3/39 | 62/17697 | 0.00034204 | 0.00488634 | 0.00370333 | CPA1/CPA2/CPB1 | 3 |
|  | MF | GO:0004806 | triglyceride lipase activity | 2/39 | 21/17697 | 0.00096782 | 0.01209778 | 0.00916885 | CEL/PNLIP | 2 |
|  | MF | GO:0008238 | exopeptidase activity | 3/39 | 113/17697 | 0.00195962 | 0.02177359 | 0.01650209 | CPA1/CPA2/CPB1 | 3 |
|  | MF | GO:0005179 | hormone activity | 3/39 | 122/17697 | 0.00243754 | 0.02437538 | 0.01847397 | GAST/INSL4/GPHA2 | 3 |
|  | MF | GO:0016298 | lipase activity | 3/39 | 127/17697 | 0.0027316 | 0.02483273 | 0.0188206 | CEL/PLA2G1B/PNLIP | 3 |
|  | MF | GO:0052689 | carboxylic ester hydrolase activity | 3/39 | 136/17697 | 0.00331427 | 0.02761889 | 0.02093221 | CEL/PLA2G1B/PNLIP | 3 |
|  | MF | GO:0008237 | metallopeptidase activity | 3/39 | 181/17697 | 0.00733924 | 0.05645573 | 0.0427875 | CPA1/CPA2/CPB1 | 3 |
|  | KEGG | hsa04972 | Pancreatic secretion | 14/28 | 102/8076 | 3.6292E-20 | 2.5404E-18 | 2.2539E-18 | AMY1B/AMY2A/CEL/CPA1/CPA2/CPB1/CTRL/PLA2G1B/PNLIP/PRSS1/CELA3A/CELA3B/CELA2A/CTRB2 | 14 |
|  | KEGG | hsa04974 | Protein digestion and absorption | 9/28 | 103/8076 | 3.5387E-11 | 1.2385E-09 | 1.0989E-09 | CPA1/CPA2/CPB1/CTRL/PRSS1/CELA3A/CELA3B/CELA2A/CTRB2 | 9 |
|  | KEGG | hsa04975 | Fat digestion and absorption | 4/28 | 43/8076 | 1.3001E-05 | 0.00030336 | 0.00026915 | CEL/CLPS/PLA2G1B/PNLIP | 4 |
|  | KEGG | hsa04970 | Salivary secretion | 3/28 | 93/8076 | 0.00393175 | 0.0580216 | 0.05147781 | AMY1B/AMY2A/CALML3 | 3 |
|  | KEGG | hsa04744 | Phototransduction | 2/28 | 28/8076 | 0.0041444 | 0.0580216 | 0.05147781 | CALML3/GUCA1C | 2 |
|  | KEGG | hsa00500 | Starch and sucrose metabolism | 2/28 | 36/8076 | 0.00679021 | 0.07921911 | 0.07028462 | AMY1B/AMY2A | 2 |
|  |  |  |  |  |  |  |  |  |  |  |
| SARC | BP | GO:0090185 | negative regulation of kidney development | 2/29 | 17/18670 | 0.00031228 | 0.09761898 | 0.07132169 | SHH/ADIPOQ | 2 |
|  | BP | GO:0050804 | modulation of chemical synaptic transmission | 5/29 | 436/18670 | 0.00050737 | 0.09761898 | 0.07132169 | CBLN1/GRIK1/ADIPOQ/CACNG5/CNTNAP4 | 5 |
|  | BP | GO:0099177 | regulation of trans-synaptic signaling | 5/29 | 437/18670 | 0.00051269 | 0.09761898 | 0.07132169 | CBLN1/GRIK1/ADIPOQ/CACNG5/CNTNAP4 | 5 |
|  | BP | GO:0010226 | response to lithium ion | 2/29 | 22/18670 | 0.00052787 | 0.09761898 | 0.07132169 | ACTA1/SHH | 2 |
|  | BP | GO:0021513 | spinal cord dorsal/ventral patterning | 2/29 | 22/18670 | 0.00052787 | 0.09761898 | 0.07132169 | SHH/LHX3 | 2 |
|  | BP | GO:0021511 | spinal cord patterning | 2/29 | 24/18670 | 0.00062949 | 0.09761898 | 0.07132169 | SHH/LHX3 | 2 |
|  | BP | GO:0014902 | myotube differentiation | 3/29 | 112/18670 | 0.00068539 | 0.09761898 | 0.07132169 | ACTA1/SHH/SMYD1 | 3 |
|  | CC | GO:0042599 | lamellar body | 2/31 | 17/19717 | 0.00032061 | 0.02180135 | 0.01586167 | SFTPB/SFTPA1 | 2 |
|  | CC | GO:0005771 | multivesicular body | 2/31 | 51/19717 | 0.00290735 | 0.05073622 | 0.03691335 | SFTPB/SFTPA1 | 2 |
|  | CC | GO:0008328 | ionotropic glutamate receptor complex | 2/31 | 51/19717 | 0.00290735 | 0.05073622 | 0.03691335 | GRIK1/CACNG5 | 2 |
|  | CC | GO:0098878 | neurotransmitter receptor complex | 2/31 | 53/19717 | 0.00313609 | 0.05073622 | 0.03691335 | GRIK1/CACNG5 | 2 |
|  | CC | GO:0045334 | clathrin-coated endocytic vesicle | 2/31 | 63/19717 | 0.0044015 | 0.05073622 | 0.03691335 | SFTPB/SFTPA1 | 2 |
|  | CC | GO:0097060 | synaptic membrane | 4/31 | 432/19717 | 0.00447673 | 0.05073622 | 0.03691335 | CBLN1/GRIK1/CACNG5/CNTNAP4 | 4 |
|  | CC | GO:0005581 | collagen trimer | 2/31 | 87/19717 | 0.00823614 | 0.08000819 | 0.05821029 | ADIPOQ/SFTPA1 | 2 |
|  |  |  |  |  |  |  |  |  |  |  |
| STAD | BP | GO:0070268 | cornification | 12/49 | 112/18670 | 9.09E-17 | 7.1811E-14 | 5.9037E-14 | IVL/KRT4/KRT13/KRT14/SPRR2B/SPRR2D/SPRR2E/SPRR3/LCE3D/KRT24/KRT78/KRT6C | 12 |
|  | BP | GO:0030216 | keratinocyte differentiation | 15/49 | 305/18670 | 1.0679E-15 | 4.2182E-13 | 3.4678E-13 | IVL/KRT4/KRT13/KRT14/S100A7/SPRR2B/SPRR2D/SPRR2E/SPRR3/LCE3D/REG3G/KRT24/KRT78/KRT6C/LCE3E | 15 |
|  | BP | GO:0009913 | epidermal cell differentiation | 15/49 | 358/18670 | 1.1355E-14 | 2.666E-12 | 2.1917E-12 | IVL/KRT4/KRT13/KRT14/S100A7/SPRR2B/SPRR2D/SPRR2E/SPRR3/LCE3D/REG3G/KRT24/KRT78/KRT6C/LCE3E | 15 |
|  | BP | GO:0031424 | keratinization | 13/49 | 224/18670 | 1.3499E-14 | 2.666E-12 | 2.1917E-12 | IVL/KRT4/KRT13/KRT14/SPRR2B/SPRR2D/SPRR2E/SPRR3/LCE3D/KRT24/KRT78/KRT6C/LCE3E | 13 |
|  | BP | GO:0008544 | epidermis development | 16/49 | 464/18670 | 2.5851E-14 | 4.0845E-12 | 3.358E-12 | IVL/KRT4/KRT13/KRT14/S100A7/SPRR2B/SPRR2D/SPRR2E/SPRR3/LCE3D/REG3G/KRT24/KRT78/KRT6C/LCE3E/KRTDAP | 16 |
|  | BP | GO:0043588 | skin development | 15/49 | 419/18670 | 1.1301E-13 | 1.4879E-11 | 1.2233E-11 | IVL/KRT4/KRT13/KRT14/S100A7/SPRR2B/SPRR2D/SPRR2E/SPRR3/LCE3D/REG3G/KRT24/KRT78/KRT6C/LCE3E | 15 |
|  | BP | GO:0018149 | peptide cross-linking | 7/49 | 60/18670 | 1.9069E-10 | 2.1521E-08 | 1.7693E-08 | IVL/SPRR2B/SPRR2D/SPRR2E/SPRR3/LCE3D/LCE3E | 7 |
|  | BP | GO:0003012 | muscle system process | 11/49 | 465/18670 | 2.5229E-08 | 2.3188E-06 | 1.9063E-06 | ACTG2/ACTN2/ATP1A2/CNN1/DES/KCNA1/MYH11/TACR2/HAND2/SYNM/HSPB6 | 11 |
|  | BP | GO:0006936 | muscle contraction | 10/49 | 360/18670 | 2.6416E-08 | 2.3188E-06 | 1.9063E-06 | ACTG2/ACTN2/ATP1A2/CNN1/DES/KCNA1/MYH11/TACR2/SYNM/HSPB6 | 10 |
|  | BP | GO:0030007 | cellular potassium ion homeostasis | 3/49 | 13/18670 | 4.7699E-06 | 0.00037682 | 0.00030979 | ATP1A2/ATP4A/ATP4B | 3 |
|  | BP | GO:0010248 | establishment or maintenance of transmembrane electrochemical gradient | 3/49 | 14/18670 | 6.0595E-06 | 0.00039892 | 0.00032796 | ATP1A2/ATP4A/ATP4B | 3 |
|  | BP | GO:0036376 | sodium ion export across plasma membrane | 3/49 | 14/18670 | 6.0595E-06 | 0.00039892 | 0.00032796 | ATP1A2/ATP4A/ATP4B | 3 |
|  | BP | GO:0045104 | intermediate filament cytoskeleton organization | 4/49 | 50/18670 | 8.823E-06 | 0.00053616 | 0.00044079 | DES/KRT14/SYNM/KRT6C | 4 |
|  | BP | GO:0045103 | intermediate filament-based process | 4/49 | 51/18670 | 9.5554E-06 | 0.0005392 | 0.00044329 | DES/KRT14/SYNM/KRT6C | 4 |
|  | BP | GO:0006883 | cellular sodium ion homeostasis | 3/49 | 17/18670 | 1.1257E-05 | 0.00059289 | 0.00048743 | ATP1A2/ATP4A/ATP4B | 3 |
|  | BP | GO:0007586 | digestion | 5/49 | 139/18670 | 3.1187E-05 | 0.00153987 | 0.00126596 | CTRB1/PGA5/PNLIP/PGA3/PGA4 | 5 |
|  | BP | GO:0090257 | regulation of muscle system process | 6/49 | 259/18670 | 5.7053E-05 | 0.00259674 | 0.00213483 | ATP1A2/CNN1/KCNA1/TACR2/HAND2/HSPB6 | 6 |
|  | BP | GO:0055075 | potassium ion homeostasis | 3/49 | 29/18670 | 5.9166E-05 | 0.00259674 | 0.00213483 | ATP1A2/ATP4A/ATP4B | 3 |
|  | BP | GO:0006937 | regulation of muscle contraction | 5/49 | 171/18670 | 8.367E-05 | 0.00347892 | 0.00286009 | ATP1A2/CNN1/KCNA1/TACR2/HSPB6 | 5 |
|  | BP | GO:0140115 | export across plasma membrane | 3/49 | 34/18670 | 9.6004E-05 | 0.00379215 | 0.0031176 | ATP1A2/ATP4A/ATP4B | 3 |
|  | BP | GO:1990573 | potassium ion import across plasma membrane | 3/49 | 43/18670 | 0.00019473 | 0.00707587 | 0.0058172 | ATP1A2/ATP4A/ATP4B | 3 |
|  | BP | GO:0006939 | smooth muscle contraction | 4/49 | 110/18670 | 0.00019705 | 0.00707587 | 0.0058172 | ATP1A2/CNN1/MYH11/TACR2 | 4 |
|  | BP | GO:0071804 | cellular potassium ion transport | 5/49 | 217/18670 | 0.00025479 | 0.00838669 | 0.00689486 | ACTN2/ATP1A2/ATP4A/ATP4B/KCNA1 | 5 |
|  | BP | GO:0071805 | potassium ion transmembrane transport | 5/49 | 217/18670 | 0.00025479 | 0.00838669 | 0.00689486 | ACTN2/ATP1A2/ATP4A/ATP4B/KCNA1 | 5 |
|  | BP | GO:0010107 | potassium ion import | 3/49 | 48/18670 | 0.00027041 | 0.00854509 | 0.00702508 | ATP1A2/ATP4A/ATP4B | 3 |
|  | BP | GO:0055078 | sodium ion homeostasis | 3/49 | 52/18670 | 0.00034298 | 0.01042147 | 0.00856768 | ATP1A2/ATP4A/ATP4B | 3 |
|  | BP | GO:0006813 | potassium ion transport | 5/49 | 240/18670 | 0.00040485 | 0.01184565 | 0.00973853 | ACTN2/ATP1A2/ATP4A/ATP4B/KCNA1 | 5 |
|  | BP | GO:0006940 | regulation of smooth muscle contraction | 3/49 | 65/18670 | 0.00066186 | 0.01867402 | 0.01535226 | ATP1A2/CNN1/TACR2 | 3 |
|  | BP | GO:0030239 | myofibril assembly | 3/49 | 73/18670 | 0.00092866 | 0.02529796 | 0.02079792 | ACTN2/MYH11/LDB3 | 3 |
|  | BP | GO:0051146 | striated muscle cell differentiation | 5/49 | 293/18670 | 0.00099724 | 0.02626067 | 0.02158938 | ACTN2/FLNC/MYH11/LDB3/SMYD1 | 5 |
|  | BP | GO:0055002 | striated muscle cell development | 4/49 | 173/18670 | 0.00109002 | 0.02777783 | 0.02283667 | ACTN2/FLNC/MYH11/LDB3 | 4 |
|  | BP | GO:0098659 | inorganic cation import across plasma membrane | 3/49 | 83/18670 | 0.0013469 | 0.03224399 | 0.02650838 | ATP1A2/ATP4A/ATP4B | 3 |
|  | BP | GO:0099587 | inorganic ion import across plasma membrane | 3/49 | 83/18670 | 0.0013469 | 0.03224399 | 0.02650838 | ATP1A2/ATP4A/ATP4B | 3 |
|  | BP | GO:0055001 | muscle cell development | 4/49 | 186/18670 | 0.00142411 | 0.03308967 | 0.02720363 | ACTN2/FLNC/MYH11/LDB3 | 4 |
|  | BP | GO:0045109 | intermediate filament organization | 2/49 | 23/18670 | 0.00164815 | 0.03720121 | 0.0305838 | DES/KRT14 | 2 |
|  | BP | GO:0031032 | actomyosin structure organization | 4/49 | 199/18670 | 0.00182396 | 0.04002576 | 0.03290592 | ACTN2/CNN1/MYH11/LDB3 | 4 |
|  | BP | GO:0051952 | regulation of amine transport | 3/49 | 95/18670 | 0.00198482 | 0.04237861 | 0.03484025 | ATP1A2/TACR2/CARTPT | 3 |
|  | BP | GO:1901018 | positive regulation of potassium ion transmembrane transporter activity | 2/49 | 26/18670 | 0.00210659 | 0.04379494 | 0.03600463 | ACTN2/KCNA1 | 2 |
|  | BP | GO:0015837 | amine transport | 3/49 | 102/18670 | 0.00243069 | 0.04923707 | 0.04047871 | ATP1A2/TACR2/CARTPT | 3 |
|  | BP | GO:0010644 | cell communication by electrical coupling | 2/49 | 29/18670 | 0.00261845 | 0.05171433 | 0.04251531 | ATP1A2/KCNA1 | 2 |
|  | BP | GO:0030004 | cellular monovalent inorganic cation homeostasis | 3/49 | 108/18670 | 0.00285853 | 0.05507896 | 0.04528144 | ATP1A2/ATP4A/ATP4B | 3 |
|  | BP | GO:0098739 | import across plasma membrane | 3/49 | 110/18670 | 0.00301078 | 0.05663141 | 0.04655773 | ATP1A2/ATP4A/ATP4B | 3 |
|  | BP | GO:0042692 | muscle cell differentiation | 5/49 | 385/18670 | 0.00328996 | 0.06044347 | 0.0496917 | ACTN2/FLNC/MYH11/LDB3/SMYD1 | 5 |
|  | BP | GO:0010927 | cellular component assembly involved in morphogenesis | 3/49 | 115/18670 | 0.00341301 | 0.06127909 | 0.05037867 | ACTN2/MYH11/LDB3 | 3 |
|  | BP | GO:0070252 | actin-mediated cell contraction | 3/49 | 116/18670 | 0.00349721 | 0.06139545 | 0.05047434 | ACTN2/ATP1A2/DES | 3 |
|  | BP | GO:0051954 | positive regulation of amine transport | 2/49 | 35/18670 | 0.00379909 | 0.06524524 | 0.05363933 | TACR2/CARTPT | 2 |
|  | BP | GO:0010667 | negative regulation of cardiac muscle cell apoptotic process | 2/49 | 38/18670 | 0.00446624 | 0.07507084 | 0.06171714 | HAND2/HSPB6 | 2 |
|  | BP | GO:0030049 | muscle filament sliding | 2/49 | 39/18670 | 0.00469981 | 0.07577238 | 0.06229388 | ACTN2/DES | 2 |
|  | BP | GO:0033275 | actin-myosin filament sliding | 2/49 | 39/18670 | 0.00469981 | 0.07577238 | 0.06229388 | ACTN2/DES | 2 |
|  | BP | GO:0010664 | negative regulation of striated muscle cell apoptotic process | 2/49 | 41/18670 | 0.00518353 | 0.08029394 | 0.06601114 | HAND2/HSPB6 | 2 |
|  | BP | GO:1901381 | positive regulation of potassium ion transmembrane transport | 2/49 | 41/18670 | 0.00518353 | 0.08029394 | 0.06601114 | ACTN2/KCNA1 | 2 |
|  | BP | GO:0030048 | actin filament-based movement | 3/49 | 138/18670 | 0.00567951 | 0.08628486 | 0.07093639 | ACTN2/ATP1A2/DES | 3 |
|  | BP | GO:0035725 | sodium ion transmembrane transport | 3/49 | 140/18670 | 0.00591025 | 0.08809623 | 0.07242555 | ATP1A2/ATP4A/ATP4B | 3 |
|  | CC | GO:0001533 | cornified envelope | 7/51 | 65/19717 | 3.1339E-10 | 3.7606E-08 | 2.6061E-08 | IVL/SPRR2B/SPRR2D/SPRR2E/SPRR3/LCE3D/LCE3E | 7 |
|  | CC | GO:0005882 | intermediate filament | 8/51 | 214/19717 | 7.2051E-08 | 4.3231E-06 | 2.9958E-06 | DES/KRT4/KRT13/KRT14/SYNM/KRT24/KRT78/KRT6C | 8 |
|  | CC | GO:0045111 | intermediate filament cytoskeleton | 8/51 | 251/19717 | 2.4488E-07 | 9.7951E-06 | 6.7878E-06 | DES/KRT4/KRT13/KRT14/SYNM/KRT24/KRT78/KRT6C | 8 |
|  | CC | GO:0044449 | contractile fiber part | 7/51 | 221/19717 | 1.5392E-06 | 4.6175E-05 | 3.1999E-05 | ACTN2/DES/FLNC/MYH11/LDB3/SYNM/SYNPO2 | 7 |
|  | CC | GO:0043292 | contractile fiber | 7/51 | 234/19717 | 2.2507E-06 | 5.4018E-05 | 3.7433E-05 | ACTN2/DES/FLNC/MYH11/LDB3/SYNM/SYNPO2 | 7 |
|  | CC | GO:0045095 | keratin filament | 5/51 | 95/19717 | 4.6029E-06 | 9.2059E-05 | 6.3795E-05 | KRT4/KRT13/KRT14/KRT78/KRT6C | 5 |
|  | CC | GO:0030018 | Z disc | 5/51 | 132/19717 | 2.2868E-05 | 0.00035445 | 0.00024563 | ACTN2/DES/FLNC/LDB3/SYNPO2 | 5 |
|  | CC | GO:0030016 | myofibril | 6/51 | 224/19717 | 2.363E-05 | 0.00035445 | 0.00024563 | ACTN2/DES/FLNC/LDB3/SYNM/SYNPO2 | 6 |
|  | CC | GO:0031674 | I band | 5/51 | 143/19717 | 3.36E-05 | 0.000448 | 0.00031046 | ACTN2/DES/FLNC/LDB3/SYNPO2 | 5 |
|  | CC | GO:0031904 | endosome lumen | 3/51 | 34/19717 | 9.2192E-05 | 0.00110631 | 0.00076665 | PGA5/PGA3/PGA4 | 3 |
|  | CC | GO:0030017 | sarcomere | 5/51 | 204/19717 | 0.00018014 | 0.00196521 | 0.00136186 | ACTN2/DES/FLNC/LDB3/SYNPO2 | 5 |
|  | CC | GO:0005771 | multivesicular body | 3/51 | 51/19717 | 0.00031105 | 0.00311055 | 0.00215555 | PGA5/PGA3/PGA4 | 3 |
|  | CC | GO:0042383 | sarcolemma | 4/51 | 136/19717 | 0.00042092 | 0.0036499 | 0.00252932 | ATP1A2/DES/FLNC/SYNM | 4 |
|  | CC | GO:0005890 | sodium:potassium-exchanging ATPase complex | 2/51 | 12/19717 | 0.00042582 | 0.0036499 | 0.00252932 | ATP1A2/ATP4B | 2 |
|  | CC | GO:0090533 | cation-transporting ATPase complex | 2/51 | 15/19717 | 0.00067409 | 0.00539271 | 0.00373705 | ATP1A2/ATP4B | 2 |
|  | CC | GO:0031143 | pseudopodium | 2/51 | 17/19717 | 0.00087022 | 0.00652665 | 0.00452286 | ACTN2/LDB3 | 2 |
|  | CC | GO:0043034 | costamere | 2/51 | 19/19717 | 0.00109056 | 0.00769808 | 0.00533463 | FLNC/SYNM | 2 |
|  | CC | GO:0098533 | ATPase dependent transmembrane transport complex | 2/51 | 21/19717 | 0.00133486 | 0.00889909 | 0.00616692 | ATP1A2/ATP4B | 2 |
|  | CC | GO:0032982 | myosin filament | 2/51 | 22/19717 | 0.00146593 | 0.00925848 | 0.00641596 | ACTG2/MYH11 | 2 |
|  | CC | GO:0005925 | focal adhesion | 5/51 | 405/19717 | 0.00385819 | 0.02262893 | 0.01568145 | ACTN2/CNN1/FLNC/S100A7/SYNPO2 | 5 |
|  | CC | GO:0005924 | cell-substrate adherens junction | 5/51 | 408/19717 | 0.00398081 | 0.02262893 | 0.01568145 | ACTN2/CNN1/FLNC/S100A7/SYNPO2 | 5 |
|  | CC | GO:0030055 | cell-substrate junction | 5/51 | 412/19717 | 0.00414864 | 0.02262893 | 0.01568145 | ACTN2/CNN1/FLNC/S100A7/SYNPO2 | 5 |
|  | CC | GO:0014704 | intercalated disc | 2/51 | 50/19717 | 0.00742311 | 0.03872929 | 0.02683872 | ATP1A2/DES | 2 |
|  | CC | GO:0016459 | myosin complex | 2/51 | 65/19717 | 0.01229739 | 0.0601342 | 0.04167195 | ACTG2/MYH11 | 2 |
|  | CC | GO:0001725 | stress fiber | 2/51 | 67/19717 | 0.01302908 | 0.0601342 | 0.04167195 | LDB3/SYNPO2 | 2 |
|  | CC | GO:0097517 | contractile actin filament bundle | 2/51 | 67/19717 | 0.01302908 | 0.0601342 | 0.04167195 | LDB3/SYNPO2 | 2 |
|  | CC | GO:0044291 | cell-cell contact zone | 2/51 | 71/19717 | 0.01454801 | 0.06465783 | 0.04480674 | ATP1A2/DES | 2 |
|  | CC | GO:0032432 | actin filament bundle | 2/51 | 75/19717 | 0.01613967 | 0.06917001 | 0.0479336 | LDB3/SYNPO2 | 2 |
|  | CC | GO:0042641 | actomyosin | 2/51 | 79/19717 | 0.01780245 | 0.0736653 | 0.05104876 | LDB3/SYNPO2 | 2 |
|  | CC | GO:0005916 | fascia adherens | 1/51 | 10/19717 | 0.02557277 | 0.09899136 | 0.06859928 | DES | 1 |
|  | CC | GO:0044224 | juxtaparanode region of axon | 1/51 | 10/19717 | 0.02557277 | 0.09899136 | 0.06859928 | KCNA1 | 1 |
|  | MF | GO:0005391 | sodium:potassium-exchanging ATPase activity | 3/49 | 11/17697 | 3.2405E-06 | 0.00018633 | 0.00012792 | ATP1A2/ATP4A/ATP4B | 3 |
|  | MF | GO:0008556 | potassium-transporting ATPase activity | 3/49 | 11/17697 | 3.2405E-06 | 0.00018633 | 0.00012792 | ATP1A2/ATP4A/ATP4B | 3 |
|  | MF | GO:0004190 | aspartic-type endopeptidase activity | 3/49 | 25/17697 | 4.3955E-05 | 0.00142575 | 0.00097878 | PGA5/PGA3/PGA4 | 3 |
|  | MF | GO:0070001 | aspartic-type peptidase activity | 3/49 | 26/17697 | 4.9591E-05 | 0.00142575 | 0.00097878 | PGA5/PGA3/PGA4 | 3 |
|  | MF | GO:0015662 | ATPase activity, coupled to transmembrane movement of ions, phosphorylative mechanism | 3/49 | 33/17697 | 0.00010266 | 0.0023611 | 0.0016209 | ATP1A2/ATP4A/ATP4B | 3 |
|  | MF | GO:0004175 | endopeptidase activity | 7/49 | 427/17697 | 0.00016227 | 0.00311014 | 0.00213511 | CTRB1/PGA5/TMPRSS11D/TMPRSS11B/TMPRSS11A/PGA3/PGA4 | 7 |
|  | MF | GO:0008307 | structural constituent of muscle | 3/49 | 46/17697 | 0.00027848 | 0.00457509 | 0.0031408 | ACTN2/MYH11/SYNM | 3 |
|  | MF | GO:0019215 | intermediate filament binding | 2/49 | 15/17697 | 0.00077065 | 0.00878825 | 0.00603313 | KRT14/SYNM | 2 |
|  | MF | GO:0019829 | cation-transporting ATPase activity | 3/49 | 66/17697 | 0.00080749 | 0.00878825 | 0.00603313 | ATP1A2/ATP4A/ATP4B | 3 |
|  | MF | GO:0022853 | active ion transmembrane transporter activity | 3/49 | 67/17697 | 0.00084371 | 0.00878825 | 0.00603313 | ATP1A2/ATP4A/ATP4B | 3 |
|  | MF | GO:0042625 | ATPase coupled ion transmembrane transporter activity | 3/49 | 67/17697 | 0.00084371 | 0.00878825 | 0.00603313 | ATP1A2/ATP4A/ATP4B | 3 |
|  | MF | GO:0015079 | potassium ion transmembrane transporter activity | 4/49 | 159/17697 | 0.00097058 | 0.00878825 | 0.00603313 | ATP1A2/ATP4A/ATP4B/KCNA1 | 4 |
|  | MF | GO:0004252 | serine-type endopeptidase activity | 4/49 | 160/17697 | 0.00099345 | 0.00878825 | 0.00603313 | CTRB1/TMPRSS11D/TMPRSS11B/TMPRSS11A | 4 |
|  | MF | GO:0003779 | actin binding | 6/49 | 431/17697 | 0.00116183 | 0.00954358 | 0.00655166 | ACTN2/CNN1/FLNC/MYH11/LDB3/SYNPO2 | 6 |
|  | MF | GO:0008236 | serine-type peptidase activity | 4/49 | 182/17697 | 0.00159818 | 0.01225275 | 0.0084115 | CTRB1/TMPRSS11D/TMPRSS11B/TMPRSS11A | 4 |
|  | MF | GO:0017171 | serine hydrolase activity | 4/49 | 186/17697 | 0.00173059 | 0.01238504 | 0.00850231 | CTRB1/TMPRSS11D/TMPRSS11B/TMPRSS11A | 4 |
|  | MF | GO:0051371 | muscle alpha-actinin binding | 2/49 | 23/17697 | 0.00183083 | 0.01238504 | 0.00850231 | LDB3/SYNPO2 | 2 |
|  | MF | GO:0005200 | structural constituent of cytoskeleton | 3/49 | 102/17697 | 0.0028257 | 0.01805311 | 0.01239344 | DES/KRT14/SYNM | 3 |
|  | MF | GO:0042626 | ATPase activity, coupled to transmembrane movement of substances | 3/49 | 115/17697 | 0.00396251 | 0.02278446 | 0.0156415 | ATP1A2/ATP4A/ATP4B | 3 |
|  | MF | GO:0043492 | ATPase activity, coupled to movement of substances | 3/49 | 115/17697 | 0.00396251 | 0.02278446 | 0.0156415 | ATP1A2/ATP4A/ATP4B | 3 |
|  | MF | GO:0051393 | alpha-actinin binding | 2/49 | 37/17697 | 0.00470197 | 0.02499796 | 0.01716107 | LDB3/SYNPO2 | 2 |
|  | MF | GO:0015405 | P-P-bond-hydrolysis-driven transmembrane transporter activity | 3/49 | 123/17697 | 0.00478222 | 0.02499796 | 0.01716107 | ATP1A2/ATP4A/ATP4B | 3 |
|  | MF | GO:0015399 | primary active transmembrane transporter activity | 3/49 | 125/17697 | 0.00500195 | 0.02500974 | 0.01716916 | ATP1A2/ATP4A/ATP4B | 3 |
|  | MF | GO:0042805 | actinin binding | 2/49 | 46/17697 | 0.0071923 | 0.0344631 | 0.02365888 | LDB3/SYNPO2 | 2 |
|  | MF | GO:0015081 | sodium ion transmembrane transporter activity | 3/49 | 149/17697 | 0.00812028 | 0.03735328 | 0.02564298 | ATP1A2/ATP4A/ATP4B | 3 |
|  | MF | GO:0015077 | monovalent inorganic cation transmembrane transporter activity | 4/49 | 382/17697 | 0.02111101 | 0.09337563 | 0.06410226 | ATP1A2/ATP4A/ATP4B/KCNA1 | 4 |
|  | KEGG | hsa04974 | Protein digestion and absorption | 6/25 | 103/8076 | 5.4088E-07 | 2.3258E-05 | 1.9927E-05 | ATP1A2/CPA1/CTRB1/PGA5/PGA3/PGA4 | 6 |
|  | KEGG | hsa04972 | Pancreatic secretion | 4/25 | 102/8076 | 0.00024743 | 0.00531972 | 0.0045579 | ATP1A2/CPA1/CTRB1/PNLIP | 4 |
|  | KEGG | hsa04971 | Gastric acid secretion | 3/25 | 76/8076 | 0.00158722 | 0.02275015 | 0.01949217 | ATP1A2/ATP4A/ATP4B | 3 |
|  | KEGG | hsa04966 | Collecting duct acid secretion | 2/25 | 27/8076 | 0.00307976 | 0.02663438 | 0.02282015 | ATP4A/ATP4B | 2 |
|  | KEGG | hsa05150 | Staphylococcus aureus infection | 3/25 | 96/8076 | 0.00309702 | 0.02663438 | 0.02282015 | KRT13/KRT14/KRT24 | 3 |
|  | KEGG | hsa04915 | Estrogen signaling pathway | 3/25 | 138/8076 | 0.00852761 | 0.06111454 | 0.05236252 | KRT13/KRT14/KRT24 | 3 |
|  |  |  |  |  |  |  |  |  |  |  |
| UCEC | BP | GO:0048771 | tissue remodeling | 5/39 | 179/18670 | 3.3864E-05 | 0.02539789 | 0.02078171 | CALCA/DBH/FGF8/TPH1/WNT16 | 5 |
|  | BP | GO:0006576 | cellular biogenic amine metabolic process | 3/39 | 55/18670 | 0.00020508 | 0.05777236 | 0.04727198 | DBH/TPH1/TRH | 3 |
|  | BP | GO:0051673 | membrane disruption in other organism | 2/39 | 11/18670 | 0.00023109 | 0.05777236 | 0.04727198 | DEFA5/DEFA6 | 2 |
|  | CC | GO:0031225 | anchored component of membrane | 4/44 | 170/19717 | 0.00055337 | 0.04039599 | 0.03727964 | DPEP1/GP2/LY6G6C/LY6L | 4 |
|  | MF | GO:0004869 | cysteine-type endopeptidase inhibitor activity | 3/39 | 58/17697 | 0.00028077 | 0.02526893 | 0.02009692 | CST1/CST2/DPEP1 | 3 |
|  | MF | GO:0005104 | fibroblast growth factor receptor binding | 2/39 | 25/17697 | 0.00137493 | 0.06187192 | 0.04920808 | FGF3/FGF8 | 2 |
|  | MF | GO:0005179 | hormone activity | 3/39 | 122/17697 | 0.00243754 | 0.07312613 | 0.05815879 | CALCA/EDN3/TRH | 3 |
|  | MF | GO:0048018 | receptor ligand activity | 5/39 | 482/17697 | 0.00393769 | 0.088598 | 0.07046391 | CALCA/EDN3/FGF3/FGF8/TRH | 5 |
|  | MF | GO:0004866 | endopeptidase inhibitor activity | 3/39 | 175/17697 | 0.00668986 | 0.09579839 | 0.07619054 | CST1/CST2/DPEP1 | 3 |
|  | MF | GO:0030414 | peptidase inhibitor activity | 3/39 | 182/17697 | 0.00745099 | 0.09579839 | 0.07619054 | CST1/CST2/DPEP1 | 3 |
|  | MF | GO:0061135 | endopeptidase regulator activity | 3/39 | 182/17697 | 0.00745099 | 0.09579839 | 0.07619054 | CST1/CST2/DPEP1 | 3 |
|  | KEGG | hsa04270 | Vascular smooth muscle contraction | 4/21 | 135/8076 | 0.00035837 | 0.03038367 | 0.02460217 | CACNA1S/CALCA/CALML3/EDN3 | 4 |
|  | KEGG | hsa04924 | Renin secretion | 3/21 | 69/8076 | 0.00071106 | 0.03038367 | 0.02460217 | CACNA1S/CALML3/EDN3 | 3 |
|  | KEGG | hsa05022 | Pathways of neurodegeneration - multiple diseases | 6/21 | 475/8076 | 0.00101891 | 0.03038367 | 0.02460217 | CACNA1S/CALML3/GPX2/DKK4/WNT16/COX7B2 | 6 |
|  | KEGG | hsa05202 | Transcriptional misregulation in cancer | 4/21 | 192/8076 | 0.00134922 | 0.03038367 | 0.02460217 | DEFA5/DEFA6/WNT16/H3C14 | 4 |
|  | KEGG | hsa04970 | Salivary secretion | 3/21 | 93/8076 | 0.00169185 | 0.03038367 | 0.02460217 | CALML3/CST1/CST2 | 3 |
|  | KEGG | hsa05010 | Alzheimer disease | 5/21 | 369/8076 | 0.00214966 | 0.03038367 | 0.02460217 | CACNA1S/CALML3/DKK4/WNT16/COX7B2 | 5 |
|  | KEGG | hsa04744 | Phototransduction | 2/21 | 28/8076 | 0.00233721 | 0.03038367 | 0.02460217 | CALML3/GUCA1C | 2 |
|  | KEGG | hsa05224 | Breast cancer | 3/21 | 147/8076 | 0.00617899 | 0.06486253 | 0.05252027 | FGF3/FGF8/WNT16 | 3 |
|  | KEGG | hsa05226 | Gastric cancer | 3/21 | 149/8076 | 0.00641498 | 0.06486253 | 0.05252027 | FGF3/FGF8/WNT16 | 3 |
|  | KEGG | hsa04310 | Wnt signaling pathway | 3/21 | 160/8076 | 0.00780964 | 0.07106771 | 0.05754471 | DKK4/WNT16/NOTUM | 3 |
